# Using an agent-based model to analyze the dynamic communication network of the immune response

**DOI:** 10.1186/1742-4682-8-1

**Published:** 2011-01-19

**Authors:** Virginia A Folcik, Gordon Broderick, Shunmugam Mohan, Brian Block, Chirantan Ekbote, John Doolittle, Marc Khoury, Luke Davis, Clay B Marsh

**Affiliations:** 1 Department of Internal Medicine, Division of Pulmonary, Allergy, Critical Care and Sleep Medicine, The Ohio State University Medical Center, Davis Heart and Lung Research Institute, Columbus, OH, USA; 2 Department of Medicine, University of Alberta, Edmonton, Alberta, Canada; 3 Department of Computer Science and Engineering, The Ohio State University, Columbus, OH, USA; 4 Department of Medicine Administration, The Ohio State University Medical Center, Columbus, OH, USA

## Abstract

**Background:**

The immune system behaves like a complex, dynamic network with interacting elements including leukocytes, cytokines, and chemokines. While the immune system is broadly distributed, leukocytes must communicate effectively to respond to a pathological challenge. The Basic Immune Simulator 2010 contains agents representing leukocytes and tissue cells, signals representing cytokines, chemokines, and pathogens, and virtual spaces representing organ tissue, lymphoid tissue, and blood. Agents interact dynamically in the compartments in response to infection of the virtual tissue. Agent behavior is imposed by logical rules derived from the scientific literature. The model captured the agent-to-agent contact history, and from this the network topology and the interactions resulting in successful versus failed viral clearance were identified. This model served to integrate existing knowledge and allowed us to examine the immune response from a novel perspective directed at exploiting complex dynamics, ultimately for the design of therapeutic interventions.

**Results:**

Analyzing the evolution of agent-agent interactions at incremental time points from identical initial conditions revealed novel features of immune communication associated with successful and failed outcomes. There were fewer contacts between agents for simulations ending in viral elimination (*win*) versus persistent infection (*loss*), due to the removal of infected agents. However, early cellular interactions preceded successful clearance of infection. Specifically, more Dendritic Agent interactions with TCell and BCell Agents, and more BCell Agent interactions with TCell Agents early in the simulation were associated with the immune *win *outcome. The Dendritic Agents greatly influenced the outcome, confirming them as hub agents of the immune network. In addition, unexpectedly high frequencies of Dendritic Agent-self interactions occurred in the lymphoid compartment late in the *loss *outcomes.

**Conclusions:**

An agent-based model capturing several key aspects of complex system dynamics was used to study the emergent properties of the immune response to viral infection. Specific patterns of interactions between leukocyte agents occurring early in the response significantly improved outcome. More interactions at later stages correlated with persistent inflammation and infection. These simulation experiments highlight the importance of commonly overlooked aspects of the immune response and provide insight into these processes at a resolution level exceeding the capabilities of current laboratory technologies.

## Background

The immune system is a dynamic network of interacting cells that communicate directly and indirectly to exchange information during an immune response. Orosz gave this complex phenomenon the name "Immuno-ecology" [1], and described in detail the properties of the immune network, likening the immune response to swarming ants. The network qualities exhibited by the immune system allow such a geographically dispersed glandular system to effectively maintain homeostasis and yet swiftly react in a *de novo*, swarm-like manner when responding to a pathogen. Immune cell activity is controlled by cell-cell interaction and by environmental signals that these and other cells produce. These signals constitute broadcast signals if they enter the blood. In contrast, cell-to-cell interactions constitute direct communication. The combination of indirect and direct communication with connections changing over time gives the immune system its network topology. The immuno-ecology view of the immune network identifies immune cells as nodes and cytokines and chemokines as edges or links between the nodes. This network topology evolves over time as cells interact, change state and eventually die. An immune response most effectively protects the body when the leukocytes rapidly eliminate pathogens and then naturally diminish in numbers (via apoptosis), avoiding damaging chronic inflammation [2-5].

In real world networks such as the world-wide web [6] and the biochemistry of living organisms [7], some nodes play a more central role than others. This network topology is called "scale-free", and is characterized by many nodes having very few links and a few "hub" nodes having many links [6]. In these cases the distribution of connections among the network nodes follows a "power-law". This hub-centric architectural design provides a high level of resilience to random loss of connections, yet makes these networks susceptible to attacks directed specifically at the hubs [8]. This scale-free topology was demonstrated in simulation experiments conducted with the Basic Immune Simulator (BIS) and has been reported previously [9].

Others have also studied the network properties of the immune system [10-12] using a growing body of biochemically validated information describing cellular signaling pathways. Fuite, Vernon and Broderick [13] extended this elemental approach by identifying signaling networks using data from high-throughput molecular assays used to survey immune and neuroendocrine status. They applied novel topological analyses to identify network features that distinguished patients with chronic fatigue syndrome (CFS) from non-fatigued subjects. In a complex illness like CFS, the identification of individual biomarkers in human data is especially difficult because of the natural heterogeneity in the magnitude of cytokines and hormones normally produced [1]. Importantly, analyzing co-expression networks improved resolution and added a new dimension to molecular phenotyping [13]. Moreover, novel therapeutic strategies could prevent or enhance indirect and direct interactions between immune cells that are causing pathological inflammation or undesired immunosuppression [1].

In these examples, the immune networks were constructed with nodes representing immune cell types and the links between the nodes represented soluble mediators such as cytokines, chemokines, or hormones. Cell-cell signals mediated by direct contact were implicitly represented. In some cases, mediators of indirect communication or stigmergy [14,15] were represented explicitly as nodes. Though revealing, these are typically static representations of network interactions and describe an average state of network assembly. The dynamic, spontaneous assembly and disassembly of network components that occurs over time were not described.

**Figure 1:**
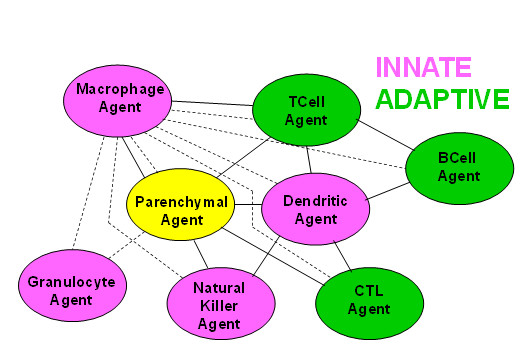
**The BIS_2010 agents representing nodes in a static representation of the immune network**. Each node in the immune network represents a category of immune cells that includes subtypes. Solid lines indicate two-way connections that involve a change in information recorded by both nodes upon contact. Dashed lines indicate interactions in which only one node, usually the Macrophage Agent, records information about the contact because the other node represents an agent that is dead. The agents representing leukocytes are pink or green, indicating their function in innate or adaptive immunity.

This study uses an agent-based model to explore the dynamics of immune network connectivity in cellular communication by direct cell-cell contact. In the static representation of the network model, discrete agents representing individual immune cells define the nodes (Figure 1). Connections between any two nodes involve direct physical contact leading to information exchange between individual immune cell agents. The agents and signals representing the various cell types and cytokines are described in additional file 1. A key advantage of using an agent-based model like the BIS_2010 (the current version of the BIS) is that it integrates experimental results from a wide range of studies, compiling them into a detailed set of known and validated interaction rules (additional file 2; [16]), and using the knowledge base in a way that allows observation and analysis of virtual cellular behavior. This agent-based approach allows a dynamic analysis of leukocyte interactions during an immune response to challenge. Though fluorescent leukocyte tagging *in vivo *continues to advance as a technology for studying cellular interaction, it is not possible to conduct analyses of immune dynamics experimentally at this level of detail and breadth, making simulation experiments highly useful.

**Figure 2:**
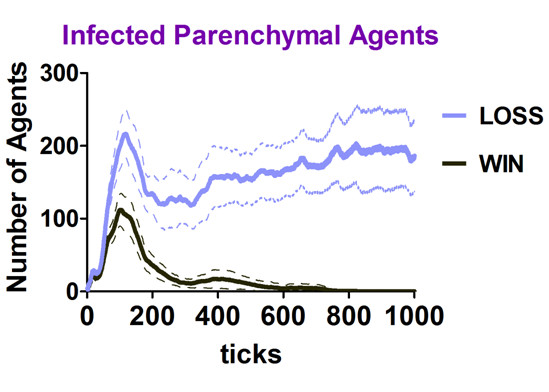
**The number of infected Parenchymal Agents for the duration of the simulation for the *win *and *loss *outcomes**. The figure shows the average number of infected Parenchymal Agents ± the 95% confidence interval (solid line and dashed lines, respectively) in Zone 1 for every tick of the simulation. The *win *outcomes (n = 100) are in black and the *loss *outcomes (n = 46) are plotted in blue.

Using this model-based approach, we identified patterns of temporally distinct network interactions that emerged from the contacts between individual agents during inflammation that led to different immunological *win *and *loss *outcomes [16]. By definition, a *win *occurred when the virtual infection of Parenchymal Agents was cleared and more than half of these agents survived or regenerated, a *loss *outcome occurred when the infection persisted in Parenchymal Agents (Figure 2), or they all died. The network interactions were also analyzed to identify characteristic features including interaction frequencies, the percent engagement of agents, and agent populations constituting functional hubs.

## Implementation

### *The Basic Immune Simulator 2010 *(BIS_2010)

The BIS and the new version, BIS_2010, were created using RepastJ [17] in Java. Its purpose is to examine the activity of the immune system during an immune response to various pathogens and injury [16]. It is an agent-based model of the immune system with representations of the cells as agents (additional file 1), these agents have specified behaviors (additional file 2), and the tissue spaces where cellular interactions take place are represented as zones (additional file 3). The adjustable parameters and their initial values are provided in additional file 4. The agents and spaces are extensions of Java classes in the RepastJ software library. The behavioral rules for the agents are described in detail in state diagrams (additional files 5, 6, 7, 8, 9, 10, 11, 12, 13, 14, 15, 16, 17, 18, 19, 20, 21, and 22). The citations for empirical demonstration of immune cell behavior are in these state diagrams describing the rules. Time is represented as discrete, sequential "ticks" that allow agent behavioral events to emulate concurrency. Space and time in the model are abstractly represented. Though duration is not strictly represented, the correct sequence of events emerges from the behavioral rules of the agents, thereby providing an event-driven chronology.

More specifically, at every tick each agent in the simulation is allowed to examine its immediate environment for signals or other agents. Each agent may react to what it detects, depending upon the rules that apply for the agent in its current state. It will only react if it detects a signal or agent relevant to its current state, and it can only change by one state, i.e. follow one edge to another state (per tick). Otherwise, it will remain in its current state until the next tick. Because many of the state changes represent behavioral events that occur within a solid tissue (as opposed to the blood), the exact quantity of time they require is unknown. Conditional control of events forces them to occur in the correct order.

One could estimate the quantity of time represented by ticks based upon the known duration of immunological events in human systems. The virus and the tissue are generic in the model and the space was based on human scale (described below), so hallmarks of the human immune response involving interactions of innate and adaptive immunity were used to estimate the time scale. The hallmarks used were the peaks of IgM and IgG antibody detection in the serum [18,19], and the peaks of virus, IgM, and IgA detection at a mucosal surface [20]. The BIS_2010 correlates were the peaks of signals Ab5 (IgM), Ab1 and Ab2 (averaged; IgG) in Zone 3 (the blood); and the peaks of the signals for Virus, Ab5, Ab1 and Ab2 (averaged; IgA) in Zone 1 (the functional tissue space), respectively. An example calculation used the peak of detection of IgM in the serum, occurring at 7-10 days [18,19]. The peak of Ab5 in Zone 3 occurred at an average of 159 ticks (simulation time increments) for the *win *outcomes (data not shown). Using 8.5 days (the average of 7 and 10 days), 159 ticks/8.5 days is 18.7 ticks/day. There are 1440 minutes/day, and (1440 minutes/day)/(18.7 ticks/day) is 77 minutes/tick, or 1.3 hours/tick. This calculation was performed using six sets of input values from above (all obtained from *win *outcomes), with two different values for the day of peak IgG detection [18,19]. The average value obtained was 64 minutes/tick (range 45-86 minutes/tick) or approximately 1 hour/tick. If this value is applied to Figure 2, the peak of infected Parenchymal Agents occurs at 4.3 days for the *win *outcome.

Space was divided into discrete compartments where relative area in the BIS_2010 Zones approximates the volume of functional human tissue (Zone 1; a representative organ, such as the lungs), the secondary lymphoid tissue (Zone 2; a group of lymph nodes and spleen), and blood (Zone 3). The volume of the lungs in an adult is estimated to be 843 ± 110 ml [21], the volume of the lymph nodes in the thorax is approximately 12 ml [22,23], and the spleen volume ranges from 180-250 ml [18]. The volume of blood in a human is approximately 5000 ml. The ratios of these volumes, roughly 1000:200:5000, were used to adjust the areas (number of [x, y] coordinates in the square) of Zones 1, 2, and 3 to 12321:2500:62500, respectively.

### Simulation Runs and Initial Conditions

**Figure 3:**
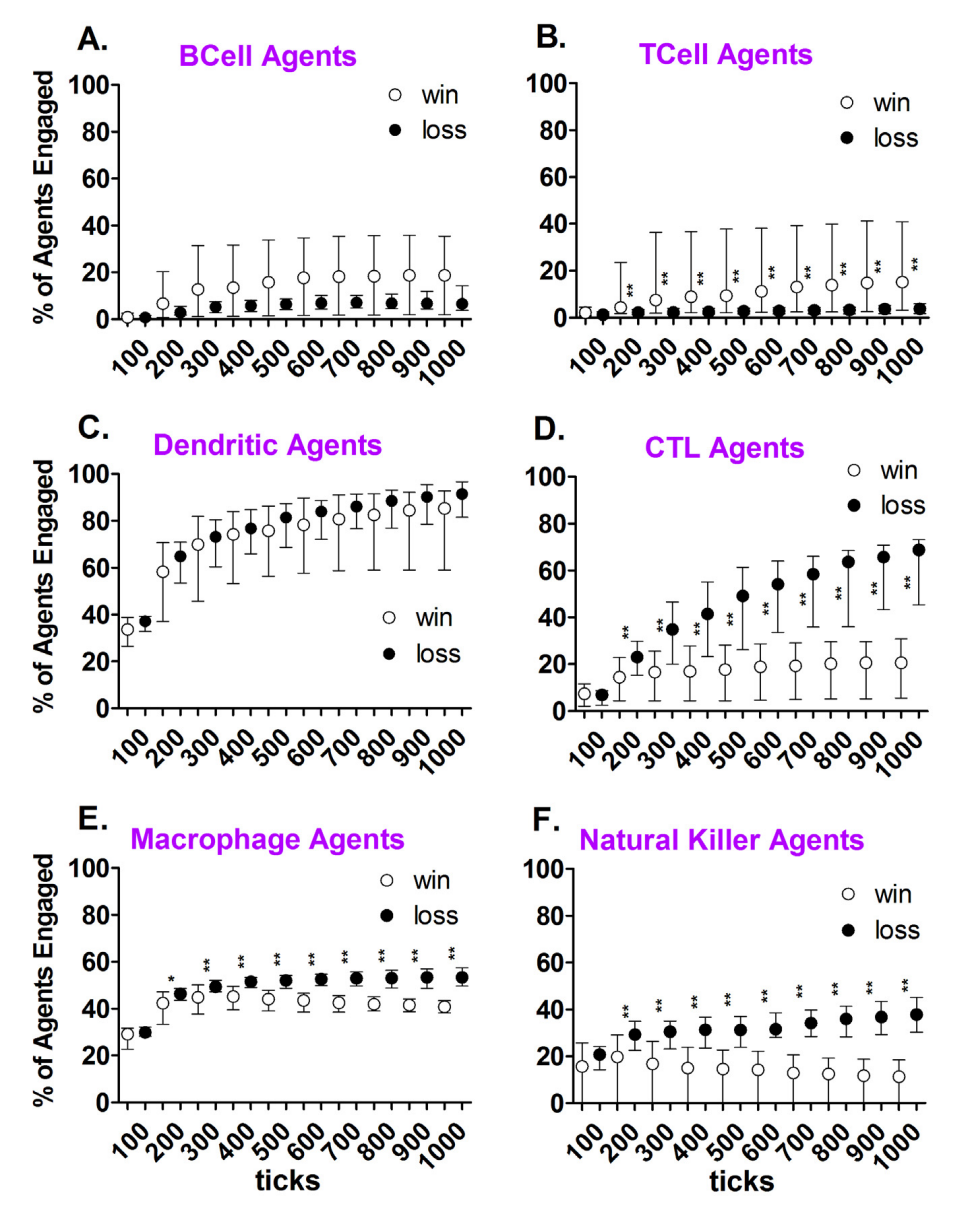
**Percentage engagement in the virtual viral immune response for each immune agent type**. The percentage of the each of the agents present in the simulation runs, in the *win *(n = 100) and the *loss *(n = 46) outcomes, that made a meaningful contact with at least one other agent were recorded cumulatively every 100 ticks. The data are expressed as the median percentage (circle) with the error bars showing the 25th and 75th percentiles. Asterisks between the *win *and *loss *results indicate significant differences at the recorded time point (**p-value < = 0.006; *p-value < = 0.012) using a two-tailed Mann-Whitney U-test with the Bonferroni correction for multiple comparisons.

**Figure 4:**
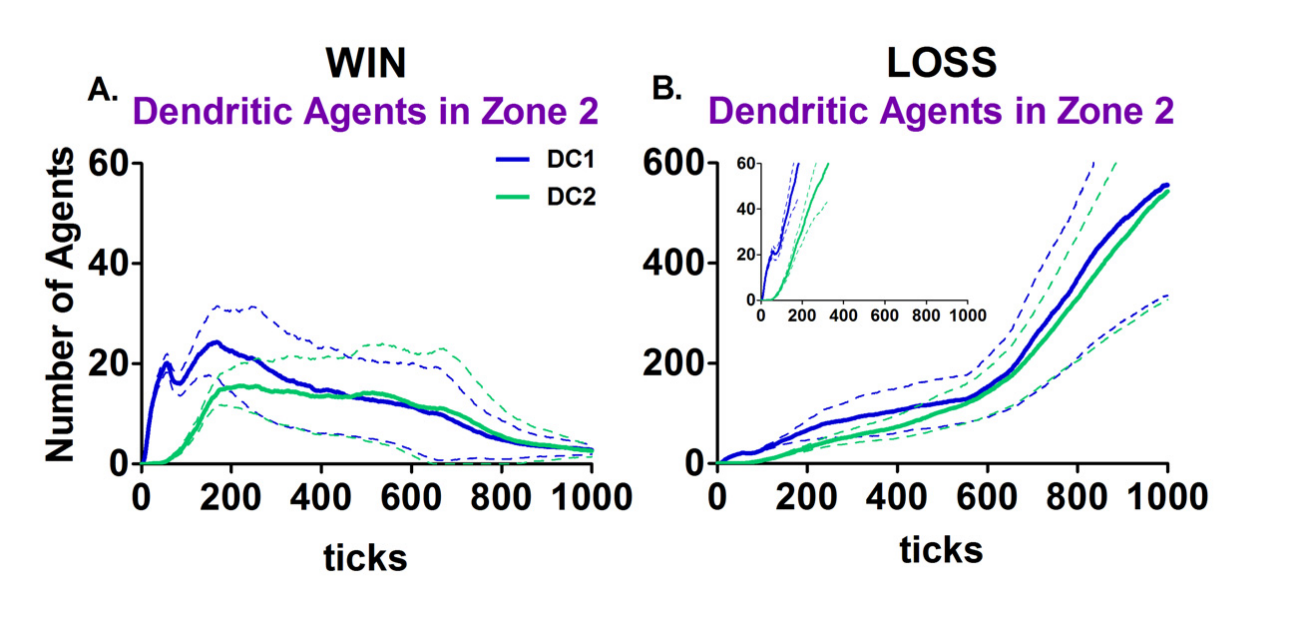
**The number of activated Dendritic Agents (DCs) in Zone 2**. A. The average number of pro-inflammatory Dendritic Agents (DC1; blue) and alternatively activated Dendritic Agents (DC2; green) ± the 95% confidence interval (solid line and dashed lines, respectively) for the *win *outcomes (n = 100). B. The average number of DC1 and DC2 ± the 95% confidence interval (solid line and dashed lines, respectively) for the *loss *outcomes (n = 46). The inset plot shows the *loss *data on the same scale as the *win *data in part A, for comparison.

**Figure 5:**
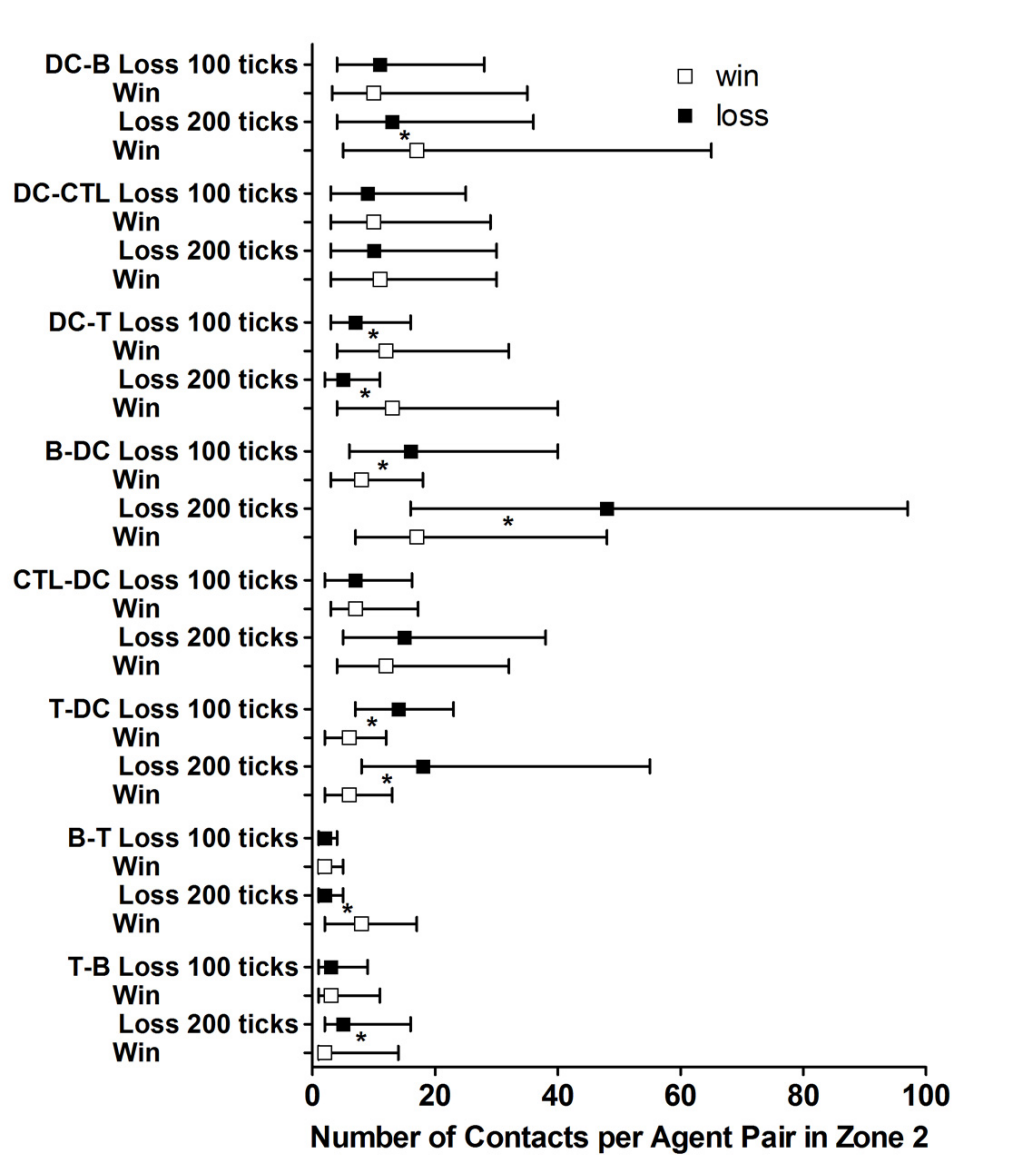
**Quantities of specific interactions between agents representing leukocytes in Zone 2**. The median (squares), 25^th ^percentile and 75^th ^percentile of number of links per node for the indicated combinations of agents and time points for the *win *(n = 100, open squares) and the *loss *(n = 46, filled squares) outcomes are shown. The first agent type listed indicates which agent recorded the contact. An asterisk between the *win *and *loss *results indicate significant differences at the recorded time point (*p-value < = 0.0016) using a two-tailed Mann-Whitney U-test with the Bonferroni correction for multiple comparisons. The abbreviations are as follows: B, BCell Agent; CTL, CTL Agent; DC, Dendritic Agent; T, TCell Agent.

**Figure 6:**
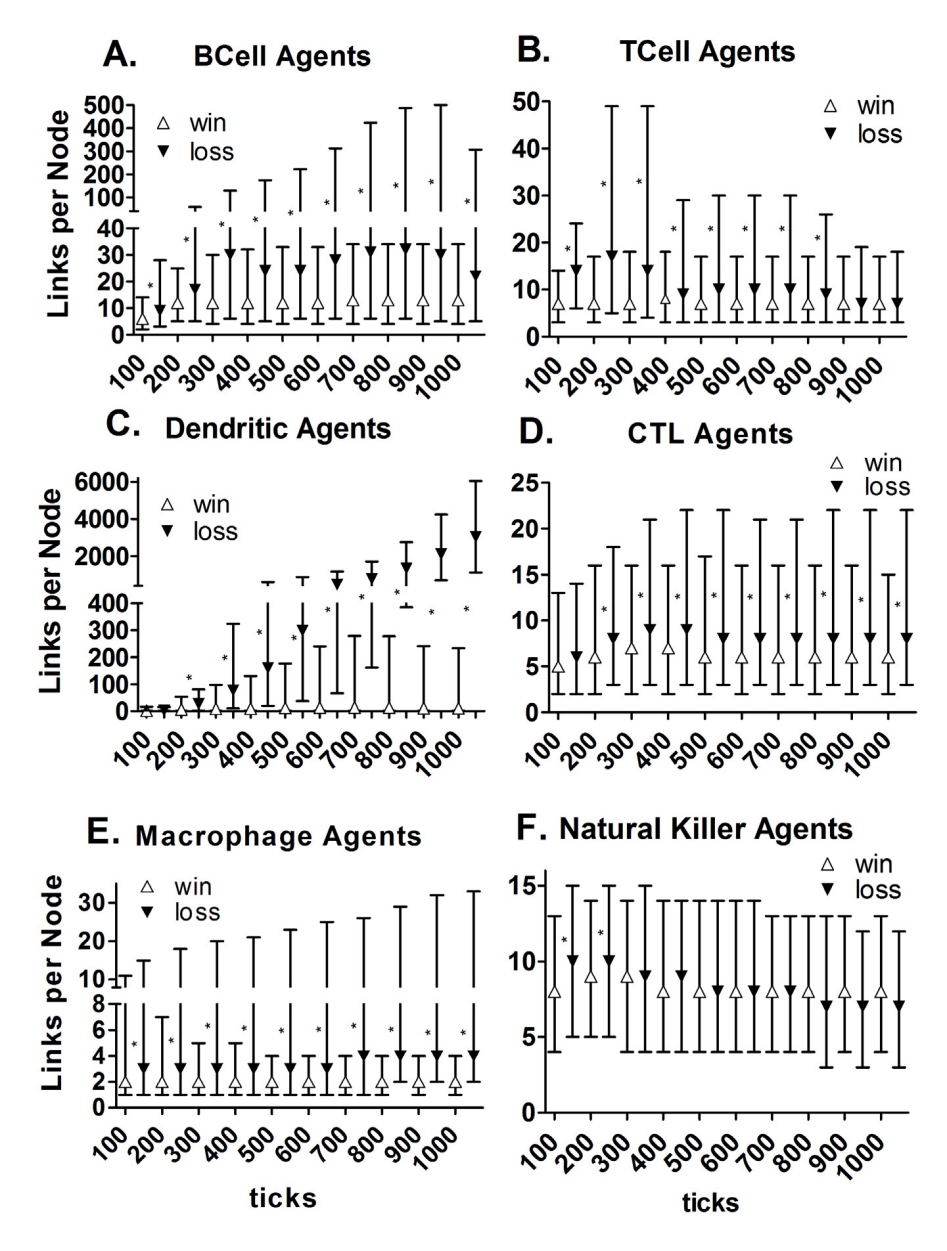
**Distributions of links per Node for each immune agent type**. The median (triangles), 25^th ^percentile and 75^th ^percentile of number of links per node for all immune agents (except Granulocyte Agents) having at least one link for the *win *(n = 100, open triangles) and the *loss *(n = 46, filled triangles) outcomes are shown. The cumulative data for links for every agent were recorded at 100 tick intervals. Asterisks between the *win *and *loss *results indicate significant differences at the recorded time point (*p-value < = 0.006) using a two-tailed Mann-Whitney U-test with the Bonferroni correction for multiple comparison.

**Figure 7:**
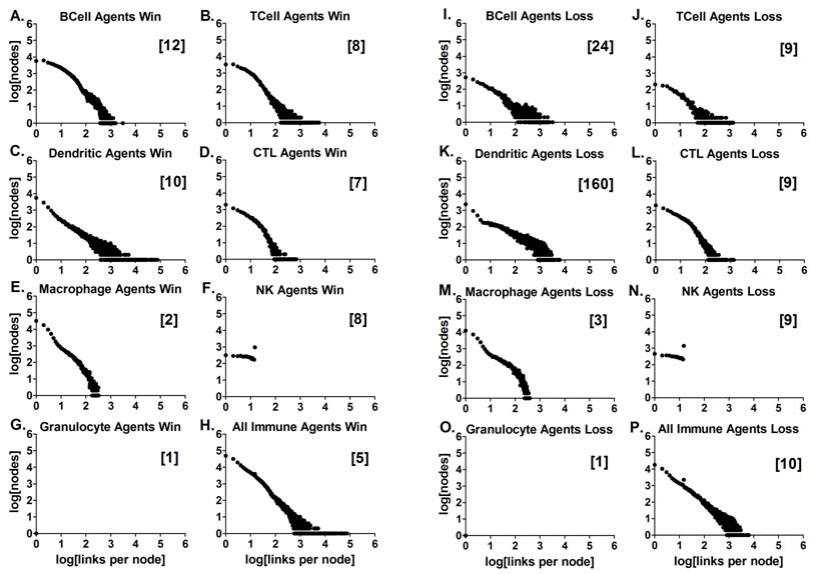
**Frequency distributions of contacts for each immune agent type at 400 ticks**. The plots show the frequency distribution of the contacts as log_10 _of the number of nodes vs. the log_10 _of the number of links per node for the first 400 ticks of the simulation for the given agent type and outcome. 7A) BCell Agent's *win *contacts, 752 points, Spearman r = -0.9418, p < 0.0001; 7I) BCell Agent's *loss *contacts, 792 points, Spearman r = -0.7857, p < 0.0001; 7B) TCell Agent's *win *contacts, 661 points, Spearman r = -0.8675, p < 0.0001; 7J) TCell Agent's *loss *contacts, 281 points, Spearman r = -0.7679, p < 0.0001; 7C) Dendritic Agent's *win *contacts, 1843 points, Spearman r = -0.8482, p < 0.0001; 7K) Dendritic Agent's *loss *contacts, 2266 points, Spearman r = -0.8936, p < 0.0001; 7D) CTL Agent's *win *contacts, 187 points, Spearman r = -0.9439, p < 0.0001; 7L) CTL Agent's *loss *contacts, 269 points, Spearman r = -0.9509, p < 0.0001; 7E) Macrophage Agent's *win *contacts, 277 points, Spearman r = -0.9586, p < 0.0001; 7M) Macrophage Agent's *loss *contacts, 317 points, Spearman r = -0.9771, p < 0.0001; 7F) Natural Killer Agent's *win *contacts, 16 points, Spearman r = -0.6676, p = 0.0047, the correlation coefficient r = -0.106 is not significant; 7N) Natural Killer Agent's *loss *contacts, 16 points, Spearman r = -0.6794, p = 0.0038, the correlation coefficient r = -0.140 is not significant; 7G, 7O) Granulocyte Agent's contacts, with only 2 points, the correlation cannot be determined; 7H) Combined immune agent's *win *contacts, 1912 points, Spearman r = -0.8954, p < 0.0001; 7P) Combined immune agent's *loss *contacts, 2274 points, Spearman r = -0.9088, p < 0.0001.

**Figure 8:**
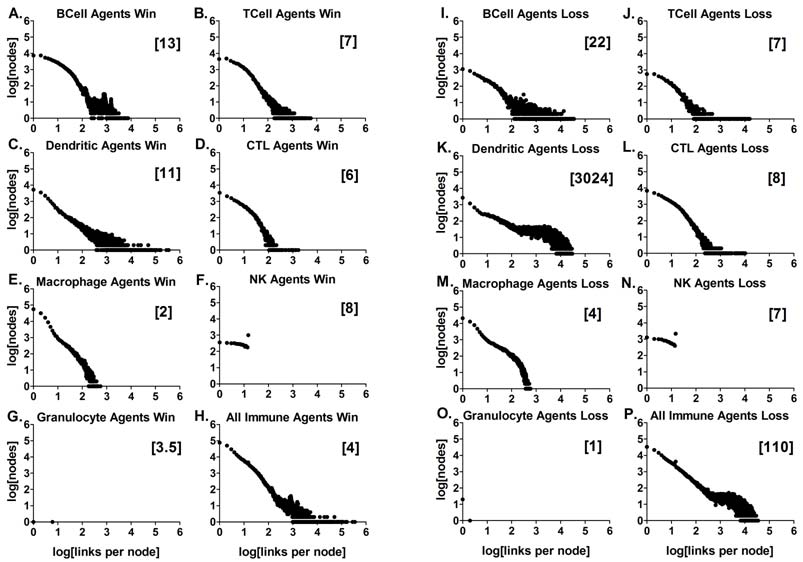
**Frequency distributions of contacts for each immune agent type at 1000 ticks**. The plots show the frequency distribution of the contacts as log_10 _of the number of nodes vs. the log_10 _of the number of links per node for all 1000 ticks of the simulation for the given agent type and outcome. 8A) BCell Agent's *win *contacts, 1732 points, Spearman r = -0.7197, p < 0.0001; 8I) BCell Agent's *loss *contacts, 2353 points, Spearman r = -0.6487, p < 0.0001; 8B) TCell Agent's *win *contacts, 756 points, Spearman r = -0.8636, p < 0.0001; 8J) TCell Agent's *loss *contacts, 368 points, Spearman r = -0.8192, p < 0.0001; 8C) Dendritic Agent's *win *contacts, 3444 points, Spearman r = -0.7669, p < 0.0001; 8K) Dendritic Agent's *loss *contacts, 17218 points, Spearman r = -0.9074, p < 0.0001; 8D) CTL Agent's *win *contacts, 216 points, Spearman r = -0.9488, p < 0.0001; 8L) CTL Agent's *loss *contacts, 580 points, Spearman r = -0.9257, p < 0.0001; 8E) Macrophage Agent's *win *contacts, 310 points, Spearman r = -0.9630, p < 0.0001; 8M) Macrophage Agent's *loss *contacts, 470 points, Spearman r = -0.9896, p < 0.0001; 8F) Natural Killer Agent's *win *contacts, 16 points, Spearman r = -0.6853, p = 0.0034, the correlation coefficient r = -0.1945 is not significant; 8N) Natural Killer Agent's *loss *contacts, 16 points, Spearman r = -0.6912, p = 0.0030, the correlation coefficient r = -0.4899 is not significant; 8G, 8O) Granulocyte Agent's contacts, too few point for correlation to be determined; 8H) Combined immune agent's *win *contacts, 3603 points, Spearman r = -0.8657, p < 0.0001; 8P) Combined immune agent's *loss *contacts, 17250 points, Spearman r = -0.9097, p < 0.0001.

A simulation run begins with all of the zones containing the numbers of agents specified in the initial conditions (additional file 4) randomly arranged (Zones 2 and 3) in whole or in part (Zone 1; additional file 3). When the BIS was first described, the initial parameters controlling the numbers of agents of different types were systematically varied and the outcomes compared [16]. Based on prior simulation runs and a parameter sweep of the number of Dendritic Agents, a (biologically) near-optimal set of experimental conditions were chosen from those producing the results shown in additional file 23 to examine the dynamics of immune network direct communication. Near-optimal was defined as the initial parameter values that resulted in a combination of a near maximal percentage of outcomes as *wins *yet enough *losses *to make comparisons of the *win *vs. *loss *data. The initial conditions chosen consisted of 200 Dendritic Agents and the other parameter values given in additional File 4. All of the data shown in Figures 2, 3, 4, 5, 6, 7, and 8, and additional files 24, 25, 26, 27, 28, and 29 came from 146 simulation runs with those initial conditions, resulting in 100 *win *outcomes and 46 *loss *outcomes for comparison.

### Agent Placement and Movement

The Parenchymal Agents representing the functional tissue cells (additional file 6) and the Portal Agents representing the entry and exit points for blood and lymphatic fluid (additional file 22) were placed in Zone 1 in the same pattern for every simulation run. Neither of these agent types moved in the tissue zone for the duration of the simulation, but they could die and be replaced depending upon the environmental conditions. The conditions for replacement included the presence of surrounding uninfected Parenchymal Agents. The same four locations (coordinates of Parenchymal Agents) were chosen for the initial sites of viral infection for all data shown. Virus and PK1 signals (additional file 1) were produced at the first tick of the simulation, and after that point every simulation run produced a unique pattern.

Randomness is an integral part of the model, and is included as described here. All random numbers were generated from a uniform distribution. The Dendritic Agents, Macrophage Agents, Granulocyte Agents, and TCell, BCell and CTL Agents were placed randomly in their zones. The few lymphocyte agents specific for the virus (and the other scenarios) were placed at randomly chosen empty coordinates among non-specific lymphocyte agents in Zone 2. Agents moved randomly unless they were attracted by signals representing chemokines. Agent movement was in increments of one [x, y] coordinate per tick. All signals diffused at each tick, using the RepastJ "diffuse" method [17], which forms concentration gradients. Random initial placement (within the appropriate zones) and random movement of the agents or non-random movement towards chemotactic concentration gradients produced by other agents generated the observed patterns. This is an accurate representation of the immune system, because the cells of the immune system are initially distributed at random in the lymphoid tissue (in the naive individual) and migrate in response to environmental cues to carry out their functions [24]. Sometimes they follow biochemical gradients (chemotaxis), and sometimes they are subject to flow forces or cellular interactions in the lymphatics and in the circulation [25,26] that may randomly change their arrival time at a new destination.

The lymphatic fluid ducts and blood vessels are represented by Portal Agents (additional file 22). When an agent leaves one zone via a Portal Agent it enters another zone at the site of a Portal Agent, under the control of environmental conditional rules at the entry site. If none of the Portal Agents in the new zone satisfy the conditions for entry (such as having the necessary chemotactic signals present proximally), the agent remains stationary and waits in a queue for the next tick. In this way, Portal Agents control the movement of agents and signals between zones. The agents or signals must be in the Portal Agent's Moore neighborhood (within the eight adjacent coordinate spaces to the Portal Agent) for this to occur. They might be considered to represent endothelial cells, which have been modeled by others for their contribution to systemic inflammation [27-29], but they are abstractly represented in the BIS_2010.

### Updates included in BIS_2010

The BIS_2010 is an updated version of the agent-based model, the BIS, that was created using RepastJ [17,30] and was previously described [16]. Because of the discovery and characterization of new types of T-helper lymphocytes including the T-helper 17 s [31-34], regulatory T cells (T-regs; [35-39]), and the T-follicular helper cells [40,41], the BIS_2010 was updated and these subtypes were added to the TCell Agent class (additional files 16, 17, and 18). Other additions included enhanced Macrophage Agent behavior (additional files 10, 11, and 12) and Granulocyte Agent behavior (additional file 21) in response to other agents in apoptotic (non- inflammatory or programmed cell death) and necrotic (inflammatory; killed by environmental factors) states. BCell Agents were updated to include more behavioral states and antibody signals (additional files 13, 14, and 15). The state diagrams for of all of the agents contain the details of their behavioral rules with literature citations, representing them as finite state automata. Agent behaviors are listed, categorized, and referenced in additional file 2. The list of references cited in the additional files is in additional file 30. Other updates to the BIS_2010 include the changes in the Zone areas described above.

### Code Verification

When changes were made to the simulation program, the code for the agents' behavior was tested to ensure that it was executing correctly before the BIS_2010 was used for experiments. Verifying the code for the behavior of the agents in the BIS_2010 is challenging because it is a program with sections of code that execute stochastically. Besides the traditional methods for verification [42], including unit testing, code walk-throughs, and observation of the visual output (additional file 3) with input parameters set to produce expected patterns, we have created a program to automate tracing of agent behavior called the AgentVerifier [43], a separate application from the BIS_2010. This is a Java application that checks state transitions for the agents and any accompanying changes in internal variable values. This process was previously done by manually reading the BIS agent behavior output files [16].

### Recording the Dynamic Network Interactions

The simulation was run and the interactions were recorded by having each agent count its contacts with other agents that either *caused one of the agents involved to change state *or *one of the agents to change a value in an internal variable*. These criteria defined "meaningful" contacts. Contacts between two agents that did not meet either of these criteria (random collisions) were not counted. Agents had to be within one coordinate space of each other (within the Moore neighborhood, radius = 1), except for Dendritic Agents, which were allowed to probe a radius of two coordinate spaces (Moore neighborhood, radius = 2). This represents the relatively large size of dendritic cells with their long dendrites [44,45]. In addition, multiple contacts between the same two agents were counted (at sequential ticks), as long as they remained in a state that recognized the contact (for example, one did not die). Thus, the BIS_2010 models processes that have been observed and recorded in living lymphoid tissue [44-53].

Each individual agent kept an ongoing record (a list of integer arrays) of their total number of meaningful contacts, including the agent types involved and the zone where the interactions took place. Both agents involved in an interaction recorded the interaction unless one of the agents was dead. Because Portal Agents represented structures and not individual cells, contacts were not recorded for these agents. Contact summaries were saved in text files with comma separated values at 100 tick intervals during each simulation run.

Signals are another major element in the BIS_2010. The signals represent cytokines and chemokines, biologically active proteins that direct migration and mediate information exchange. All of the signals that the agents produce are listed in additional file 1. Cytokines and chemokines drive cell-cell interaction by providing indirect communication or "stigmergy" [15], and have been considered to form a network of communication among the cells of the immune system [1,10,11,13]. Signals in the BIS_2010 control agent behavior by causing state transitions. The state of an agent determines whether the agent recognizes a contact with another agent or a signal, simulating the presence or absence of surface receptors on cells. Production of a signal may be common to multiple agent types, as described in additional file 1. For efficiency of execution, the BIS_2010 was not implemented in a manner that allows determination of the agent source of a signal. The impact of signals on network formation was implicitly captured in the network of direct communication events between agents.

### Statistical Analyses

Non-parametric statistical methods were used unless otherwise indicated. A very conservative Bonferroni correction was applied to correct the alpha value used when estimating significance in multiple comparisons. Thus the alpha value necessary for significance was made smaller by dividing it by the number of comparisons made for a data set. Outcomes at regular time intervals were analyzed using separate statistical comparisons to simplify interpretation. GraphPad Prism version 5.03 was used to create the plots in the figures and perform the statistical analyses.

## Results

### Simulation outcomes

The initial conditions for the simulation runs used for the network analysis were chosen (from those shown in additional file 23) to provide mostly immune *win *outcomes but enough *loss *outcomes for comparisons to be made. In the *win *outcomes, all of the infected Parenchymal Agents were eliminated, usually within the first half of the simulation run (Figure 2). In the *loss *outcomes, more Parenchymal Agents became infected by the time 100 ticks had passed, and the virtual immune response failed to eliminate all of the infected agents. The data shown in the remainder of the figures came from the same simulation runs as the data shown in Figure 2. There were many ways to present the data derived from these experiments; the results are presented from an immunologist's perspective.

### Participation of agents in mounting a typical immune response

A characteristic of the immune response demonstrated by the BIS_2010 was that most of the agent types representing leukocytes never made meaningful contacts with any other agents (Figure 3). Agents having had at least one meaningful contact (in any Zone) were considered "engaged" in the immune response, and the extent of engagement of each agent type for the response duration was determined. Zero percent of the agents were engaged at the start of a simulation run. The median percent engaged and inter-quartile ranges are plotted in Figure 3 (with 100 tick intervals) and show the cumulative history of engagement of each agent type for the duration of the simulation runs. The levels of engagement for simulation runs that ended in *win *outcomes are separated from the same data for *loss *outcomes. The results show that with the exception of the Dendritic and BCell Agents (Figure 3C and 3A), the median percent of agents engaged from the *win *and *loss *outcomes diverged significantly at 200 ticks into the simulation.

The Dendritic Agents displayed the greatest increases in engagement at the beginning (100 and 200 ticks) of the virtual immune response (Figure 3C). This initial surge corresponds to the recognition of antigen by contact with infected Parenchymal Agents in Zone 1 (Figure 2; additional file 7). The Dendritic Agents are activated by contact with the infected Parenchymal Agents and migrate to Zone 2 (Figure 4; additional file 8). Figure 4 shows the average numbers of Dendritic Agents of two different phenotypes, DC1 s representing the pro-inflammatory type and DC2 s the alternatively activated type [54-62]. Note the ten-fold difference in the scale for the *loss *outcomes, in part B, and the inset in part B for comparison to part A. The numbers of activated Dendritic Agents that travel to Zone 2 (lymph node equivalent) decreases after the infected Parenchymal Agents have been nearly eliminated (Figure 2) between 200 and 300 ticks for the *win *outcome (part A), but not in the loss outcome (part B). In Zone 2 the Dendritic Agents seek BCell, TCell and CTL Agents to make contact and present antigen (Figure 5). The lag in engaging these lymphocytic agent types (Figure 3A, B, and 3D) is due to the time required by the Dendritic Agents to encounter the few viral antigen-matched lymphocyte agents in Zone 2 [26,46,63]. The requirement for viral antigen-specificity also accounts for the low level of lymphocyte agent engagement, especially apparent for the BCell Agents and TCell Agents in Figure 3.

The Natural Killer (NK) Agents and Macrophage Agents (Figure 3E and 3F) represent cells involved in the innate immune response (additional files 9, 10, 11, and 12). A greater percentage of these agents became engaged in the *loss *than in the *win *outcome. This is because the NK and Macrophage Agents continued to be engaged by infected Parenchymal Agents in Zone 1 (Figure 2) in the *loss *outcome. Macrophage Agents had significantly more contacts with Parenchymal Agents at 100 and 200 ticks in the *loss *vs. the *win *outcome (data not shown). The drop in percentage of engaged agents in the *win *outcomes for Macrophage and NK Agents indicated an increase in the number of agents present that were not engaged (Figure 3E and 3F). The engaged NK Agents in Zone 1 were killing the virally infected Parenchymal Agents or their "stressed" neighbors (additional files 6 and 9). The median number of contacts with Parenchymal agents per NK Agent was significantly greater for the *loss *vs. the *win *outcome at 100 and 200 ticks (data not shown), because there were more infected Parenchymal Agents (Figure 2).

Specific interactions between agents in each zone were counted and the results from Zone 2 (the lymphoid tissue zone) are shown in Figure 5. The contact pairs in Figure 5 are listed with the agent recording the contact first. Since both agents would record a contact between them, the contacts are counted by two agent types in the figure. After arrival to present antigen in Zone 2, significantly higher frequencies of contacts per Dendritic Agent with the TCell and BCell Agents at these early time points were associated with the *win *outcome. No outcome-associated differences in the Dendritic Agents' median quantity of contacts with CTL Agents were found at 100 and 200 ticks (Figure 5).

In contrast, significantly fewer Dendritic Agent contacts were made per BCell Agent for the *win *vs. the *loss *outcomes at 100 and 200 ticks in Zone 2 (Figure 5). BCell Agents also made antibody after contact with virus [64,65], but BCell Agent contacts with free virus were not counted in these analyses. The effector TCell, CTL, and BCell Agents generated by this initial activity then migrated via Zone 3 (the blood) to Zone 1 to eliminate the virally infected Parenchymal Cells directly or via antibody production (additional files 24, 25, 26 and Figure 2).

The median number of contacts per TCell Agent with BCell and Dendritic Agents was not greater for the *win *outcome (Figure 5). However, the *win *outcomes were associated with far more TCell Agents making at least one specific contact with a BCell Agent (fourteen times more per simulation run by 100 ticks) or a Dendritic Agents (ten times more per simulation run by 100 ticks) than the *loss *outcomes in Zone 2 (data not shown). This reiterates the role for TCell Agent engagement for a *win *(Figure 3B).

The CTL Agents had a greater percentage of agents engaged in the *loss *outcome (Figure 3D), and more were present in Zone 1 (the functional tissue zone) in the *loss *outcome (additional file 26). The CTL Agents contacted Dendritic Agents in Zone 2, but there were no differences in the median number of these contacts per CTL Agent (Figure 5) in Zone 2 at 100 or 200 ticks for the *win *and *loss *outcomes.

The TCell and BCell Agents need contact with each other in Zone 2 for full activation and antibody production to occur (additional files 13 and 14; [40,52]). The median number of contacts per BCell Agent with TCell Agents was not different for the first 100 ticks of *win *vs. *loss *outcomes, but more TCell Agent contacts per BCell agent by 200 ticks was associated with the *win *outcome (Figure 5). In addition, there were seven times as many BCell Agents that made specific contact with TCell Agents in the same interval for *wins *vs. *losses *(data not shown). By 200 ticks, there were almost nine times as many BCell Agents that had made contact with a TCell Agent in Zone 2 for the *win *vs. the *loss *outcomes (data not shown).

### Interaction history for engaged agents over time

The median number of contacts and the interquartile range (± 25th percentile) were plotted to describe the overall contact history at every 100 ticks of the simulation in all zones with all agent types (Figure 6A, B, C, D, E, and 6F).

The BCell Agents and TCell Agents had a higher percentage of agents engaged in the virtual immune response when there was a *win *outcome (Figure 3A and 3B), but the median number of contacts or links per node were greater in the *loss *outcome throughout the time course (Figure 6A and 6B). Because the data presented in Figure 6 are cumulative, increases in the numbers of agents without contacts at the end of simulation runs resulted in decreases in the median links per node (Figure 6A, B, and 6F). For the TCell and BCell Agents (Figure 6A and 6B) these are memory cells (additional files 24 and 25). NK Agents (Figure 6F) are likely unable to make contacts (kill remaining infected Parenchymal Agents) because anti-inflammatory cytokines predominate (additional files 9 and 28). These are produced by Macrophage Agents of type 2, or anti-inflammatory Macrophage Agents ([62]; additional files 10 and 27).

In contrast to the BCell and TCell Agents, the CTL Agents and Natural Killer Agents had a higher percentage of agents engaged when there was a *loss *outcome (Figure 3), and CTL Agents also had more links per node for the *loss *outcome after 100 ticks (Figure 6D). NK Agents had higher median numbers of links per node at 100 and 200 ticks for the *loss *outcome (Figure 6F). The difference between cytotoxic T lymphocytes and natural killer cells is that the cytotoxic T lymphocytes require antigen presentation in the lymph node (to become activated to kill) but the natural killers cells do not [66,67], so there was less delay in engagement and killing for the NK Agents. Although there were no significant differences in the number of contacts per CTL Agent at 100 ticks between the *win *and *loss *outcomes (Figure 6D), from 200 ticks onward the CTL Agents had more contacts per agent in the *loss *outcome.

The pattern observed for the Macrophage Agents was more agents engaged (Figure 3E) and more contacts (Figure 6E) in the *loss *outcomes than the *win *outcomes. As scavengers, the Macrophage Agents (Figure 6E) interacted with all agents, in most cases when the agents had died via apoptosis (Figure 1). Engaged Macrophage Agents had the most contacts per agent with Parenchymal Agents (specifically) compared to all other agents at 100 ticks, and significantly more in the *loss *outcomes (data not shown), perhaps because they contacted more infected and apoptotic Parenchymal Agents and then killed and/or phagocytosed them ([68-70]; additional file 10).

### The frequency distributions of the immune network contacts for the BIS_2010

The frequency distributions of the meaningful contacts or links between the agents representing leukocytes (nodes) are shown in Figures 7 and 8, and additional files 31, 32, and 33. These plots are the logarithmically transformed, aggregate data from the *win *outcomes separated from the aggregate *loss *outcomes, with the cumulative numbers of links per node for data collected at 400 ticks and 1000 ticks (Figures 7 and 8, respectively). The median number of links per node is in brackets for the engaged agents in the aggregate data. These "time" points in the simulation run were chosen because at 400 ticks most of the agents' activities (contacts with other agents) had reached a level near the end-point value (Figure 6). The frequency distribution is a power law distribution in all cases except for the data from the Granulocyte Agents and the Natural Killer Agents. The functional relationship was tested on the raw data using a Spearman non-parametric test for correlation. Similar statistical results were obtained using linear regression on the log-transformed data (not shown). Remarkably similar results were obtained when other initial conditions were used, including 20, 100, and 300 Dendritic Agents, (1000 ticks shown in additional files 31, 32, and 33; statistics are summarized in additional file 34).

Thus the frequency distribution of the network interactions between immune cell-agents during the simulated immune response is scale-free, suggesting that the network interactions of the immune system are as well. This result is not surprising for the system as a whole (Figure 7H and 7P, and Figure 8H and 8P) given that the interactions between elements in complex biological systems can generally be characterized as scale-free [7,9,71-74]. The frequency distributions for most of the different agent types were also scale-free, with the exceptions mentioned above, while the Granulocyte Agents did not have enough data points to test.

The agents representing granulocytes had the fewest agents engaged (data not shown). They also had the lowest median number of links (Figure 7G and 7O and Figure 8O). This is because granulocyte behavior, killing pathogens via release of reactive oxygen species and potent protease enzymes, is dependent upon activation by signals or cytokines/chemokines (additional file 21; [75]). Granulocytes have surface receptors to make physical contact with pathogens such as bacteria, parasites and fungi [76]. The Granulocyte Agent activation did not require receptor-mediated contact with other agents, but there were conditions where a few made contact with dying agents and necrotic debris, a condition thought to mimic pathogen contact (Figure 8G and 8O; [77]).

### Immune system hubs

The Dendritic Agents had far more links than the other agents representing immune cells, making them hub agents of the virtual immune system (Table 1). Without representation of the necessity for antigen presentation to the adaptive immune cells, Figure 1 does not convey that Dendritic Agents are hubs. The analysis of the numbers of contacts between agents revealed this quality. By 200 ticks, a greater percentage of the Dendritic Agents were engaged compared to the other immune agents (Figure 3). As antigen presenting cells, dendritic cells may have more meaningful contacts than other immune cell types [78]. However, the median numbers of links for Dendritic Agents in the simulation seemed higher than expected at the end of the simulation runs and notably in the *loss *outcomes (Figure 6). This phenomenon was examined further (Table 2) to determine which agents the Dendritic Agents were interacting with at such high rates, and in which zone this was occurring.

**Table 1:** Median Number of Links for All Agents

Agent Type	Ticks									
	**100**	**200**	**300**	**400**	**500**	**600**	**700**	**800**	**900**	**1000**

**BCell Agents**	6	12	12	13	13	13	13	13	13	13

**CTL Agents**	6	7	8	8	8	7	7	7	7	8

**Dendritic Agents**	2	14	28	49	95	183	415	849	1495	2366

**Granulocyte Agents**	0	0	1	1	1	1	1	1	1	1

**Macrophage Agents**	2	2	2	2	2	2	2	2	2	2

**Natural Killer Agents**	9	9	9	9	8	8	8	8	7	7

**TCell Agents**	7	8	8	8	7	7	7	7	7	7

**All**	3	6	6	6	6	6	7	8	9	11

The median numbers of contacts for each agent type representing a leukocyte is given, including only the engaged agents. These are the contacts from all 146 simulation runs, including the *win *outcome and the *loss *outcomes combined. The results are shown for every 100 ticks of the simulation runs.

**Table 2:** Dendritic Agent Median Contacts at 1000 ticks

Outcome	WIN		LOSS	
**Agent Type**	Zone 1	Zone 2	Zone 1	Zone 2

**BCell Agents**	2	47	1	27

**CTL Agents**	2	17	1	9

**Dendritic Agents**	1	79	1	3085

**Granulocyte Agents**	1	1	1	1

**Macrophage Agents**	1	1	1	1

**Natural Killer Agents**	1	1	1	1

**TCell Agents**	1	10	1	5

The median numbers of contacts that each engaged Dendritic Agent had with each agent type representing an immune cell type is given. The contacts are separated into columns of those accumulated in Zone 1 and then Zone 2, with the contacts from Zone 1 included with those in Zone 2. The data from the simulation runs are also separated into columns by their outcome (*wins*, n = 100; *loss*, n = 46).

### Dendritic Agent contacts with each agent type in Zones 1 and 2

The striking difference in the number of contacts for Dendritic Agents between the *win *and *loss *outcomes was even more surprising when it was determined that most of the contacts were between Dendritic Agents themselves (Table 2). The Dendritic Agents interacted with other agents first in Zone 1 (tissue), and then in Zone 2 (lymph nodes; Figure 4). The data for the number of contacts made in Zone 2 includes the contacts from Zone 1 because the counting was not reset when the agents changed zones. Besides contacting themselves in Zone 2, the engaged Dendritic Agents actually had more contacts (higher medians) for their numbers of contacts with BCell, TCell and CTL Agents in the *win *outcomes than in the *loss *outcomes (Table 2). This models the antigen-presenting function of dendritic cells. The numerous contacts between Dendritic Agents at the end represented an emergent phenomenon for the simulation, with biological correlation discussed below.

## Discussion

The value of applying network theory to understand relationships embedded in biological data is becoming better appreciated [74]. Defining the interactions between elements of biological systems as networks and characterizing the topology has broad application, from the study of protein-protein interactions in small organisms [7,79-82] to evolution of the proteins of the immune system [83] to defining networks of disease genotypes [73] and phenotypes [13,84,85]. The arbitrary definition of nodes and edges distinguishes all of the biological network analyses, and determines what information can be derived from the application of network theory to biological mechanisms.

Here we chose to define the network nodes as agents representing cells of the immune system, and edges as physical contacts made between agents representing immune cell types (via implied surface receptors) during a simulated immune response. Since an immune response to a pathogen is a dynamic process that involves sequential steps and movement of cells to different locations in the body to communicate information, the network defined above requires a dynamic analysis. This type of analysis cannot be conducted quantitatively in a living organism, but it can be conducted virtually using an agent-based model of the immune system.

The frequency distribution of the interactions between the agents representing immune cells was found to be scale-free for a range of starting conditions (Figure 8 and additional files 31, 32, and 33), and the agents representing dendritic cells acted as hubs in the immune system network. This is consistent with our earlier results, using a simpler version of the simulation [9]. The new observations from this work include the analysis of the agent interactions recorded over virtual time, the individual agent types recording the types of agents they contacted, and in which zone the interactions were taking place.

One observation that arose from the assessment of the interactions between the immune cell agents was that the majority of agents present did not interact during the simulation (Figure 3). In general, the agents representing innate immune cells (Dendritic, Macrophage, and Natural Killer Agents) were more engaged at the beginning of the simulation (Figure 3C, E, and 3F; [56,66,86,87]). NK cells kill infected cells, but without the prior need for antigen presentation that adaptive immune cells require [67,88,89]. Macrophages innately recognize pathogens and infected, apoptotic or necrotic cells (additional files 10, 11, and 12), but antibody attachment to infected cells or pathogens also helps macrophages find their targets [90]. The cells of the innate immune system, by definition, are ready to fight common pathogens at the first detection of "danger signals" [91-95]. In contrast, Granulocyte Agents had the least meaningful interactions of all of the agent types (Table 1) because they were programmed, like neutrophils, to become activated by signals or pathogens in the environment, rather than by specific interactions with other agents [75,76].

The overall lack of engagement for the other agent types is consistent with the real functioning of the immune system, validating the behavioral rules for the agents of the BIS_2010. Normal immune responses do not engage all of the cells of the immune system and in fact, biological conditions that involve overactive immune system engagement are septic shock [96] or systemic inflammatory response syndrome [77]. These conditions involve a systemic inflammatory response, with or without detectable infection, and a mortality rate near 50%.

Our goal was to determine what characteristics of the immune system's communication metrics distinguished successful elimination of virus, or the *win *outcome, from the *loss *outcome. Both the numbers of agents making contacts (specific engagements) as well as the numbers of specific contacts per agent were compared. Communication between specific agents early in the simulation runs was found to be critical. Significantly more contacts between Dendritic Agents and both TCell and BCell Agents, occurring at 200 ticks or earlier, were associated with the *win *outcome (Figure 5). In Zone 2, far more TCell Agents contacted BCell Agents and Dendritic Agents in the first 100 ticks for the *win *outcomes (data not shown), but the median numbers of these specific contacts per TCell Agent did not differ (Figure 5). The same was true for the number of BCell Agents contacting TCell Agents. Additional files 24 and 25 show the effector and memory TCell and BCell Agents generated by the specific contacts that migrated to Zone 1 (via Zone 3). These results are consistent with the data in Figure 3 showing that only the BCell Agents and TCell Agents had more agents engaged during the simulation runs with the *win *outcome. The necessity for rapid communication, namely antigen presentation to lymphocyte agents, is valid simulation behavior for a *win *outcome [56].

CTL Agents also had to make specific contact with Dendritic Agents in Zone 2 (additional file 19) and migrate to Zone 1 to kill infected Parenchymal Agents (additional file 20) [97-99]. Unexpectedly, early in the simulation, the numbers of Dendritic Agent contacts with CTL Agents was not different (Figure 5). In the *loss *outcome, CTL Agents did not encounter Dendritic Agents and proliferate soon enough, so more Parenchymal Agents became infected (due to the continuous replication of virus) and significantly more CTL Agents became engaged. More effector CTL Agents made their way to Zone 1 (via Zone 3) in the *loss *outcome (additional file 26).

The most striking emergent outcome was the difference in the number of links per node in the *win *and *loss *outcomes for the Dendritic Agents (Figure 6), as well as the abundance of Dendritic Agents in Zone 2 in the *loss *outcome (Figure 4). The increase in the numbers of Dendritic Agents in Zone 2 at the end of the simulation runs with the *loss *outcomes is consistent with the high median numbers of contacts between these agents shown in Table 2, and the "lump" of points with more links per node in Figure 8K at 1000 ticks, compared to Figure 7K at 400 ticks. This outcome is validated by studies using *in vivo *using dual-laser microscopy [26]. Dendritic cells carry out tissue surveillance, phagocytose and process antigens, and present them to lymphocytes in the lymph node [56,100,101]. During inflammatory responses the lymph nodes become engorged with cells, and dendritic cells have a role in tissue remodeling of the lymph node to accommodate the influx [102]. They spread themselves along fibro-reticular networks in lymph nodes [103]. This requires "stepping" over other dendritic cells in the search for an open space on a fibroblast. As such, this migration process involves contacting other dendritic cells. The searching for an open space behavior was programmed into Dendritic Agents as normal behavior (additional file 8; [104]). The structural organization in lymph nodes enhances the ability of dendritic cells to probe incoming T-lymphocytes to find an antigen-matched lymphocyte [44,103]. T-lymphocytes travel from lymph node to lymph node [105], and must traverse the long processes of dendritic cells that are probing them [104], a process which adds to the contacts. The reason for the high numbers of contacts in the *loss *outcome for Dendritic Agents was the abundance of infected Parenchymal Agents in Zone 1, and the stimulation of more Dendritic Agents that traveled to Zone 2. This phenomenon is emergent, and only occurs in the *loss *outcome, validating the agent behavior rules in the BIS_2010.

As the previous model had shown [106], the Dendritic Agents represent the immune cells that are the hubs of the immune system. One could hypothesize that for preventing an undesired immune response such as transplant rejection, the best cellular target for therapy would be dendritic cells leaving the transplant tissue. Testing such a hypothesis would require a method for stopping the dendritic cells before they presented antigen in the lymph nodes. The dendritic cells also make signals that attract other leukocytes to the tissue, so such signals would have to be neutralized as well. This would be a difficult hypothesis to test, because both direct and indirect communication by the dendritic cells would have to be eliminated. In addition, only those dendritic cells presenting antigen from the graft should be targeted, to avoid diminishing the immune response to other pathogens.

In summary, we found that recording and analyzing direct interactions between agent types representing leukocytes using an agent-based model recapitulated what is believed to occur *in vivo *during an immune response. The results support the immuno-ecology idea that the more rapid the initial response, the better, despite the highly decentralized nature of the system's components. In addition, effectiveness is more important than efficiency for the immune system [1]. Agent-based modeling allows the analysis of the dynamic interactions between the elements of complex systems, a unique approach for biology.

## Conclusions

To gain insight into complex systems like the immune system, it is necessary to expand the types of approaches that we use to study them. An agent-based model of any complex system could be used to study its dynamic network interactions. Here we have used the agent-based modeling approach in combination with a dynamic network analysis to virtually observe what cannot be observed *in vivo*. All disease processes involve the immune system at some level. Any insight that can be gained through the application and combination of modeling and network analyses to the current body of knowledge of the immune system is valuable information. New techniques for analyzing collected scientific data in immunology are important for understanding disease processes and finding new ways to intervene.

## Availability and requirements

The new version, BIS_2010 is available as a Java archive file (jar) at: http://digitalunion.osu.edu/r2/summer06/sass/download.html and as additional file 35. The BIS_2010.jar file must be downloaded as well as the RepastJ launcher (Repast_J_3.1_Installer.exe), available at: http://sourceforge.net/projects/repast/files/.

The source code was written in Java and compiled with Eclipse [107] using Java Runtime Environment 6, so a JRE version of at least 6.0 must be installed on the computer used to run RepastJ and the BIS_2010. Once the necessary software is on the computer, the RepastJ executable jar must be run as per the instructions included with the software. The RepastJ toolbar will appear and one must click on the folder on the left. A "Load Model" panel will appear, and in the "Demo Models" list one must scroll down and choose "Other Models", then "Add". The "Open" panel allows one to locate the BIS_2010.jar and "Open" it. Then "Load" must be chosen in the "Load Model" panel to make the Graphical User Interface (GUI) appear. In the "Parameters" menu at least three choices must be made. First, one must choose to set one of the challenges to the immune system at the top of the list. "Set_ViralInfection:" and "Set_Bacti:" are the verified choices. A '1' must be typed in to replace the '0' for one of the choices. Following that are "StopSimulationAt:" and "CountIncrement". If these are unchanged, the simulation will run for 1001 ticks and collect contact data every 100 ticks. One text file will be recorded with the quantities of all elements of the simulation at every tick if it reaches the "StopSimulationAt:" tick. Two text files will be recorded every time the "countIncrement" is passed. All of these files will appear in the RepastJ folder. To prevent many files from being generated, the "CountIncrement" can be set equal to "StopSimulationAt:". Any other parameters may be altered, and the simulation is started with the triangle in the toolbar. The simulation can be stopped with the square button in the toolbar.

The source code will only be made available through collaboration agreement with the contact author.

## List of Abbreviations and Definitions

**Agent**: the interactive entities of the model with rules for behavior; **behavior**: what the agents are programmed to do, listed in additional file 2; **BIS**: Basic Immune Simulator; **degree**: the number of edges or links a node has with other nodes; **edge**: a connection between two nodes, the same as a link; **emergent behavior**: behavior that occurs as an unforeseen consequence of the combinations of rules from other agents; **engaged**: an agent that has made one or more contacts, **hub**: a node in a network that has the most edges or links; **imposed behavior**: intentionally programmed behavior (behavior necessary for a successful immune response); **leukocyte**: a white blood cell, all leukocytes are part of the immune system; **link**: a connection between two nodes, the same as an edge; **Moore neighborhood**: the eight adjacent locations to a central location in a rectangular matrix; **node connectivity**: the number of edges or links a node has with other nodes; **power-law distribution**: a frequency distribution that fits the funcion y = αx^β^; **scale-free distribution**: a frequency distribution that spans several powers of a base number; **signal**: a representation of diffusing substances produced by the agents; **state diagram**: a diagram (directed graph) of a finite state automaton; **stigmergy**: the indirect communication between agents allowed by changes to the environment such as signal production; **tick**: a discrete time step in the simulation; **zone**: a virtual environment where agents and signals interact.

## Competing interests

The authors declare that they have no competing interests.

## Authors' contributions

VAF created, updated and modified the BIS_2010 to collect data for network analysis. She also conceived of the AgentVerifier, and modified the BIS_2010 to produce output for the AgentVerifier created by CE, JD, SM, BB and MK. VAF wrote the initial draft of the manuscript. GB and CBM made substantive intellectual contributions to the manuscript and interpretation of data, as well as the drafting and revision of the manuscript. SM, JD, CE, BB and MK designed and created specialized software for data analysis including and in addition to the AgentVerifier. CE, JD and LD were involved in drafting parts of the manuscript. All authors were involved in revising the manuscript critically for intellectual content, and have given their final approval for the version to be published.

## Supplementary Material

Additional file 1 **Table of agents, cells, signals, and soluble mediators**. The BIS_2010 agents and signals and their corresponding cells and soluble mediators A table listing all of the elements in the simulation, with citations. Click here for file

Additional file 2 **Table of citations for agent behaviors**. Summary of citations for agent behaviors. A table listing the agents, their behaviors, and citations for the simulation rules regarding their behaviors. Click here for file

Additional file 3 **Zone 1: A generic tissue space**. A screen-shot showing the appearance of the simulation when it runs. Click here for file

Additional file 4 **A list of all parameters**. Initial parameter values. A list of all of the parameters in the simulation, most of which are accessible from the GUI. Click here for file

Additional file 5 **Key to state diagrams**. A key to the symbols and colors in the state diagrams. Click here for file

Additional file 6 **State diagram: Parenchymal Agents (PC), Zone 1**. A state diagram of the potential PC behavioral sequences in Zone 1. Click here for file

Additional file 7 **State diagram: Dendritic Agents (DCs) Zone 1**. A state diagram of the potential DC behavioral sequences in Zone 1. Click here for file

Additional file 8 **State diagram: Dendritic Agents (DCs), Zone 2**. A state diagram of the potential DC behavioral sequences in Zone 2. Click here for file

Additional file 9 **State diagram: Natural Killer Agents (NKs) in Zone 1**. A state diagram of the potential NK behavioral sequences in Zone 1. Click here for file

Additional file 10 **State diagram: Macrophage agents (MΦs), Zone 1**. A state diagram of the potential MΦ behavioral sequences in Zone 1. Click here for file

Additional file 11 **State diagram: Macrophage Agents (MΦs) Zone 2**. A state diagram of the potential MΦ behavioral sequences in Zone 2. Click here for file

Additional file 12 **State diagram: Macrophage Agents (MΦs), Zone 3**. A state diagram of the potential MΦ behavioral sequences in Zone 3. Click here for file

Additional file 13 **State diagram: BCell Agents (Bs) in Zone 2 (Part 1)**. A state diagram of the potential B behavioral sequences in Zone 2. Click here for file

Additional file 14 **State diagram: BCell Agents (Bs) in Zone 2 (Part 2)**. A state diagram of the potential B behavioral sequences in Zone 2. Click here for file

Additional file 15 **State diagram: BCell Agents (Bs) in Zones 3 and 1**. A state diagram of the potential B behavioral sequences in Zones 3 and 1. Click here for file

Additional file 16 **State diagram: TCell Agents (Ts) in Zone 2 (Part 1)**. A state diagram of the potential T behavioral sequences in Zone 2. Click here for file

Additional file 17 **State diagram: TCell Agents (Ts) in Zone 2 (Part 2)**. A state diagram of the potential T behavioral sequences in Zone 2 Click here for file

Additional file 18 **State diagram: T Cell agents (Ts) in Zone 1**. A state diagram of the potential T behavioral sequences in Zone 1. Click here for file

Additional file 19 **State diagram: Cytotoxic T Lymphocyte Agents (CTLs) in Zones 2 and 3**. A state diagram of the potential CTL behavioral sequences in Zones 2 and 3. Click here for file

Additional file 20 **State diagram: Cytotoxic T Lymphocyte Agents (CTLs) in Zone 1**. A state diagram of the potential CTL behavioral sequences in Zone 1. Click here for file

Additional file 21 **State diagram: Granulocyte Agents (GRAN) in Zones 3, 1**. A state diagram of the potential GRAN behavioral sequences in Zones 3 and 1. Click here for file

Additional file 22 **State diagram: Portal Agents (Portals)**. A state diagram describing the Portals. Click here for file

Additional file 23 **Percentage of *win *or *loss *outcomes and ticks to eliminate infected agents for different starting conditions**. A figure showing the outcomes when the initial number of Dendritic Agents is varied. Click here for file

Additional file 24 **The number of Effector and Memory TCell Agents in Zone 1 for the duration of the simulation for the *win *and *loss *outcomes**. A figure that shows the average numbers of TCell Agents in Zone 1 for the duration of the simulation. Click here for file

Additional file 25 **The number of Effector and Memory BCell Agents in Zone 1 for the duration of the simulation for the *win *and *loss *outcomes**. A figure that shows the average numbers of Effector and Memory BCell Agents in Zone 1 for the duration of the simulation. Click here for file

Additional file 26 **The number of Effector and Memory CTL Agents in Zone 1 for the duration of the simulation for the *win *and *loss *outcomes**. A figure that shows the average numbers of Effector and Memory CTL Agents in Zone 1 for the duration of the simulation. Click here for file

Additional file 27 **The number of Macrophage Agents in Zone 1 for the duration of the simulation for the *win *and *loss *outcomes**. A figure that shows the average numbers of Macrophage Agents in Zone 1 for the duration of the simulation. Click here for file

Additional file 28 **The number of Natural Killer Agents in Zone 1 for the duration of the simulation for the *win *and *loss *outcomes**. A figure that shows the average numbers of Natural Killer Agents in Zone 1 for the duration of the simulation. Click here for file

Additional file 29 **The number of Granulocyte Agents in Zone 1 for the duration of the simulation for the *win *and *loss *outcomes**. A figure that shows the average numbers of Granulocyte Agents in Zone 1 for the duration of the simulation. Click here for file

Additional file 30 **Additional file references**. A list of references cited in all of the additional files. Click here for file

Additional file 31 **Frequency distributions of contacts for each immune agent type at 1000 ticks with a starting condition of 20 Dendritic Agents**. A figure that shows the frequency distribution of contacts for each immune agent type at the end of the simulation (120 simulation runs combined; 20 Dendritic Agents starting condition) with the *win *and *loss *outcomes separated. Click here for file

Additional file 32 **Frequency distributions of contacts for each immune agent type at 1000 ticks with a starting condition of 100 Dendritic Agents**. A figure that shows the frequency distribution of contacts for each immune agent type at the end of the simulation (120 simulation runs combined; 100 Dendritic Agents starting condition) with the *win *and *loss *outcomes separated. Click here for file

Additional file 33 **Frequency distributions of contacts for each immune agent type at 1000 ticks with a starting condition of 300 Dendritic Agents**. A figure that shows the frequency distribution of contacts for each immune agent type at the end of the simulation (120 simulation runs combined; 300 Dendritic Agents starting condition) with the *win *and *loss *outcomes separated. Click here for file

Additional file 34 **Table of statistics for **Figure 8** and **additional files 31, 32**, and **33. A table of statistics including the number of data points, Spearman r value for correlation and the p-value for the correlation statistic for all of the frequency distribution diagrams for each agent shown in Figure 8 and additional files 31, 32, and 33. Click here for file

Additional file 35 **An executable jar file for the simulation, BIS_2010**. Click here for file
