# Supplementary material for: Using an agent-based model to analyze the dynamic communication network of the immune response
Source: Theor Biol Med Model. 2011 Jan 19;8:1. doi: 10.1186/1742-4682-8-1 (PMC3032717; doi:10.1186/1742-4682-8-1)
Supplement: Additional file 1 — Table of agents, cells, signals, and soluble mediators. The BIS_2010 agents and signals and their corresponding cells and soluble mediators A table listing all of the elements in the simulation, with citations. [file 1742-4682-8-1-S1.PDF]

Additional file 1 - The BIS_2010 agents and signals and their corresponding cells and soluble mediators

**BIS_2010 AGENT TYPES AND THE ZONES THEY OCCUPY**	**IMMUNE CELLS REPRESENTED AND THEIR FUNCTIONAL DESCRIPTION**	**BIS_2010 SIGNALS**		**CYTOKINES, CHEMOKINES[1] AND MOLECULES REPRESENTED BY EACH SIGNAL**
Parenchymal Cell Agent (PC)	Functional tissue cells	(Parenchy-malkine 1) PK1		Stress factors such as Heat Shock Proteins [2], Uric Acid [3], and Chemerin [4], HMGB1 [5, 6] chemokines such as CX3CL1, CCL3, CCL5, CCL6
		Virus		Virus particles
		Bacteria		Bacteria
		Apoptotic bodies		Apoptotic bodies or dead cells associated with programmed cell death
		Necrosis factors		Fragments of intra-cellular proteins or structures associated with death by necrosis, HMGB1 [5]
Dendritic Agent (DC1, DC2)<br><br>Zones 1, 2.	Tissue surveillance, antigen presentation	MK1 (Monokine 1)		IL-12 [7], IL-1[8, 9], IL-8 (CXCL8)[10], CCL3, CCL4, CCL5, CXCL9, CXCL10, CXCL11
		MK2		IL-10, IL-4, CCL1, CCL17, CCL22, CCL11, CCL24, CCL26
		MK6		IL-6 [8, 9, 11, 12]
Macrophage Agent (MΦ1, MΦ2)<br><br>Zones 1, 2, 3	INNATE immunity. Scavenging of dead cell debris, INNATE immunity	MK23		IL-23 [8, 9, 11-16]
		MK27		IL-27 [13, 17]
TCell Agent (T0, T1,T2, T follicular helper, T17, Treg)<br><br>Zones 2,3,1 [18]	T_{HELPER} lymphocytes (CD4+), cell-mediated, ADAPTIVE immunity	CK1 (Cytokine 1)	T1	IFN-γ [16, 19], IL-2, TNF-α
		CK2	T2	IL-4 [16, 19], IL-5, IL-13, IL-2
		CK21	T4	IL-21 [8, 9, 11, 20, 21]
		CK17	T17	IL-17 [8, 9, 11, 12, 14-16, 19-23]
		TGFβ	T3	TGF-β [8, 9, 11, 20, 22]
Cytotoxic T Lymphocyte Agent (CTL Agent)<br>Zones 2,3,1 [18]	$T_{CYTOTOXIC}$ lymphocytes (CD8+), cell-mediated, ADAPTIVE immunity	CK1		IFN-γ
Natural Killer Agent (NK)<br>Zone 1	Natural Killer Cells, cell-mediated immunity, kills stressed cells, INNATE immunity	CK1		IFN-γ
BCell Agent (B1,B2,B5)<br><br>Zones 2,3,1	B Lymphocytes, ADAPTIVE, humoral immunity, makes antibodies	Ab1 (Antibody 1)		Cytotoxic and neutralizing antibody; IgG [24, 25], IgA [26]
		Ab2		Targeting and neutralizing antibody; IgG [24, 25], IgA
		Ab5		Targeting and neutralizing antibody; IgM [24, 25]
		Complement		Bound antibody catalyzes complement product formation, C3a, C5a [27]
Granulocyte Agent (Gran)<br>Zones 3,1	Neutrophils, Eosinophils and Basophils, INNATE immunity, releases enzymes and toxins by degranulation and produces reactive oxygen species	(Degranulation product 1) G1		Degranulation products, reactive oxygen products
Portal Agent<br>Zones 1,2,3	Blood vessels, lymphatic ducts. The only agent representing a structure rather than a cell type.			

The agents and the zones that they inhabit are in the first column, with the order of the zone numbers indicating the potential direction for migration. The second column lists the cells represented by the agents. Some agents represent multiple cell types, such as the Granulocyte Agents. The third column lists the signals produced by the agent type. Note that signals may be produced by multiple agent types. For example, both Dendritic Agents and Macrophage Agents produce the signals in blue font. All agents may produce apoptotic bodies and necrosis factors. The last column lists the major cytokines and chemokines represented by the signal in the same row.
