# Supplementary material for: Using an agent-based model to analyze the dynamic communication network of the immune response
Source: Theor Biol Med Model. 2011 Jan 19;8:1. doi: 10.1186/1742-4682-8-1 (PMC3032717; doi:10.1186/1742-4682-8-1)
Supplement: Additional file 2 — Table of citations for agent behaviors. Summary of citations for agent behaviors. A table listing the agents, their behaviors, and citations for the simulation rules regarding their behaviors. [file 1742-4682-8-1-S2.PDF]

Additional file 2. - Summary of citations for agent behaviors

Agent types	Behaviors	Citations
Parenchymal agent (PC)	Signal production	[2-5]
	Neighbor detection/contact/killing	[28]
	Migration	Not applicable (NA)
	Proliferation	NA
	Death	[27, 29-33]
Dendritic Cell agent (DC)	Signal detection	[7, 34-39]
	Signal production	[7, 13, 34, 40-45]
	Neighbor detection/contact/killing	[34, 40, 41, 46-59]
	Migration	[1, 34, 52, 54, 57]
	Proliferation	[34]
	Death	[47, 55, 57, 59-62]
Macrophage agent (MΦ)	Signal detection	[2, 27, 30, 49, 63-70]
	Signal production	[45, 63, 69, 71, 72]
	Neighbor detection/contact/killing	[30, 32, 33, 49, 73]
	Migration	[1, 2, 27]
	Proliferation	NA
	Death	[74, 75]
T Cell agent (T)	Signal detection	[8, 9, 11, 12, 16, 20-22, 34, 39, 41, 44, 49, 76-85]
	Signal production	[41, 49, 80]
	Neighbor detection/contact/killing	[34, 39, 41, 47, 49, 51, 52, 56, 57, 86-90]
	Migration	[1, 52, 56, 57]
	Proliferation	[12, 18, 21, 47, 79, 80, 83, 85, 86]
	Death	[28, 59, 88, 91-94]
Cytotoxic T Lymphocyte agent (CTL)	Signal detection	[80, 95]
	Signal production	[53, 96, 97]
	Neighbor detection/contact/killing	[18, 53, 56, 57, 95, 96, 98-100]
	Migration	[1, 56, 57, 100]
	Proliferation	[18, 80, 95]
	Death	[101]
Natural Killer agent (NK)	Signal detection	[2, 17, 29, 42, 102]
	Signal production	[17, 97, 103]
	Neighbor detection/contact/killing	[29, 51, 101, 102, 104, 105]
	Migration	[1]
	Proliferation	NA
	Death	[101]
B Cell agent (B)	Signal detection	[46, 67, 106-110]
	Signal production	[30, 46, 106, 109, 111-113]
	Neighbor detection/contact	[46, 57, 87, 89, 106, 110, 114, 115]
	Migration	[1, 52, 57, 106, 111]
	Proliferation	[46, 87, 106, 116]
	Death	[46, 106]
Granulocyte agent (Gran)	Signal detection	[1, 23, 27, 117-121]
	Signal production	[31, 122]
	Neighbor detection/contact/killing	[31, 32, 122]
	Migration	[1, 120, 121]
	Proliferation	[31]
	Death	[31, 123, 124]
Portal Agent (Portal)	Signal detection	[1]
	Signal production	[1]
	Neighbor detection/contact/killing	[1]
	Migration	[1]
	Proliferation	NA
	Death	[32]
