# Supplementary material for: Using an agent-based model to analyze the dynamic communication network of the immune response
Source: Theor Biol Med Model. 2011 Jan 19;8:1. doi: 10.1186/1742-4682-8-1 (PMC3032717; doi:10.1186/1742-4682-8-1)
Supplement: Additional file 3 — Zone 1: A generic tissue space. A screen-shot showing the appearance of the simulation when it runs. [file 1742-4682-8-1-S3.PDF]

Additional file 3 – Zone 1: A generic tissue space

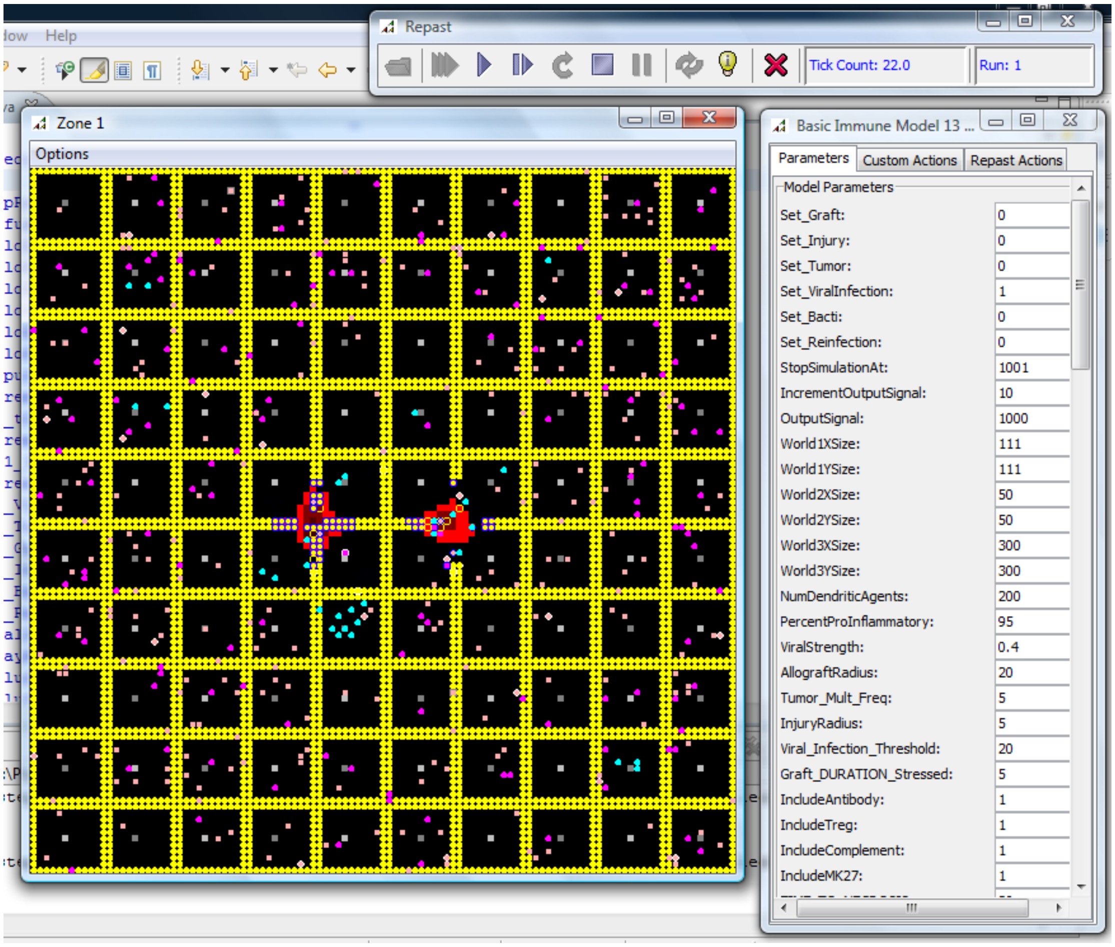

An example of Zone 1 from BIS_2010 at the beginning (tick 22) of the viral infection scenario. The RepastJ graphical user interface toolbar and Model Parameters panel are shown as well. The size of Zone 1 is 111x111 (representing approximately 1 mm^2), and the parameters World1XSize and World1YSize are in the list of model parameters. The yellow filled circles are Parenchymal Agents, the ones with blue rings are infected, Dendritic Agents are magenta, Macrophage Agents are pink, Natural Killer Agents are turquoise, Portal Agents are light and dark gray, and the virus is red.
