# Supplementary material for: Using an agent-based model to analyze the dynamic communication network of the immune response
Source: Theor Biol Med Model. 2011 Jan 19;8:1. doi: 10.1186/1742-4682-8-1 (PMC3032717; doi:10.1186/1742-4682-8-1)
Supplement: Additional file 4 — A list of all parameters. Initial parameter values. A list of all of the parameters in the simulation, most of which are accessible from the GUI. [file 1742-4682-8-1-S4.PDF]

Additional file 4 – Initial parameter values

Affects All
evapRate = 0.99
diffusionConstant = 1.0
world1XSize = 111
world1YSize = 111
world2XSize = 50*
world2YSize = 50*
world3XSize = 250
world3YSize = 250
outputSignal = 1000
incrementOutputSignal = 10
set_ViralInfection = 1
set_Tumor = 0
set_Graft = 0
set_Injury = 0
set_Bacti = 0
set_Reinfection = 0
viralStrength = 0.3
challengeXCoordinate = world1XSize/2
challengeYCoordinate = world1YSize/2
percentTAntiViral = 0.607
percentTAntiBacti = 0.607
percentTAntiTumor = 0.4
percentTAntiAllo = 2
stopSimulationAt = 1001
countIncrement = 100
pauseVal = -1
includeMK27 = 1
TIME_TO_NECROSIS = 30
PK1_Threshold = 300
IL2_threshold = 1000
incrementIL2_threshold = 100
DURATION_CK_Zone1 = 25
DURATION_CK_Zone2 = 25
DURATION_MK1_Zone1 = 25
DURATION_MK2_Zone1 = 25
DURATION_MK_Zone2 = 25

Affects BCell Agents
percentBAntiViral = 0.67
percentBAntiBacti = 0.67
percentBAntiTumor = 1
percentBAntiAllo = 2
duraEmitAbZ3 = 25
duraEmitAbZ2 = 25
duraEmitAbZ1 = 25
includeAntibody = 1
includeComplement = 1
B_CK_THRESHOLD = 150
B_MK_THRESHOLD = 150
numB_ToSend = 2
LIFE_B_Zone1 = 25
LIFE_B_Zone2 = 25
LIFE_B_Zone3 = 50
num_Memory_B = 0

Affects CTL Agents
percentCTLAntiViral = 0.883
percentCTLAntiBacti = 0
percentCTLAntiTumor = 0.4
percentCTLAntiAllo = 2
numCTLToSend = 2
LIFE_CTL_Zone1 = 25
LIFE_CTL_Zone2 = 25
LIFE_CTL_Zone3 = 50
num_Memory_CTL = 0
NUM_TICKS_NO_CK1orPK1orMK1 = 50

Affects Dendritic Agents
numDendriticAgents = 200**
numDCToSend = 1
LIFE_DC_Zone1 = 150
LIFE_DC_Zone2 = 50
LIMIT_NUM_Ts = 12
percentProInflammatory = 95

Affects Granulocyte Agents
numGranZ3denom = 100***
LIFE_GRAN_Zone1 = 15

Affects Macrophage Agents
MO_TIME_TO_NECROSIS = 60
numMOZ1 = 500
LIFE_MO_Zone1 = 200
LIFE_MO_Zone2 = 50
LIFE_MO_Zone3 = 100

Affects Natural Killer Agents
numNKToSend = 2
LIFE_NK_Zone1 = 25
NUM_TICKS_NK_NO_KILL = 15
NK_KILL_LIMIT = 15
DURATION_NK_CK1 = 25

Affects Parenchymal Agents
delayRegenerationTime = 5
Virus_THRESHOLD = 200
injuryRadius = 5
DURATION_Stressed = 25
viral_Infection_Threshold = 20

Affects Portal Agents
DURATION_Ab1_Zone1 = 100
DURATION_Ab2_Zone1 = 100
DURATION_Ab5_Zone1 = 100
zone3_Zone1_Ab_Factor = 180

Affects TCell Agents
includeTreg = 1
numT1_ToSend = 2
numT2_ToSend = 2
activT_DC_Z2_Contacts = 10
LIFE_T0_Zone1 = 15
LIFE_T_Zone1 = 25
LIFE_T_Zone2 = 25
LIFE_T_Zone3 = 50
T_MAX_KILLS = 10
num_Memory_T = 0

*The value for the number of lymphoid agents (BCell Agents, CTL Agents, and TCell Agents) is calculated from the size of Zone 2:

numLymphoidCells = (world2XSize * world2YSize) = 2500

The value for the number of Macrophages in Zone 3 is calculated from the numLymphoidCells:

numMOinBlood = numLymphoidCells/20 = 125

The value for the number of Macrophages in Zone 2 is calculated from the numMOinBlood:

numMOinZone2 = numMOinBlood/5 = 25

**This parameter value was varied in additional files 23, and 31, 32, and 33.

*** The value for the number of Granulocyte Agents is calculated from the size of Zone 3 and the numGranZ3denom:

numGranulocytes = (world3XSize * world3YSize)/ numGranZ3denom = 6
