# Supplementary material for: Using an agent-based model to analyze the dynamic communication network of the immune response
Source: Theor Biol Med Model. 2011 Jan 19;8:1. doi: 10.1186/1742-4682-8-1 (PMC3032717; doi:10.1186/1742-4682-8-1)
Supplement: Additional file 5 — Key to state diagrams. A key to the symbols and colors in the state diagrams. [file 1742-4682-8-1-S5.PDF]

Additional File 5 - Key to state diagrams

State 0	The initial state
State X	A final state, death
Blue text	A description of the state
ALL CAPS	A signal is produced by the agent in the state
GREEN	A signal produced in response to bacteria
PURPLE	A signal produced in response to virus
BLUE	A signal produced in response to many stimuli
Red text	A condition that causes a transition to another state
ALL CAPS	A signal that causes a state transition
Magenta text	A state transition that involves addition of agents
Some input value	An input parameter value
Initialization	Conditions existing at the beginning of a simulation run
·························►	A state transition, usually annotated with what causes it
Zone X	An arrow to/from a box like this indicates migration to/from another Zone

All of the state diagrams for the behavior of the agents use the same symbols. Any input parameters referred to from additional File 4 are underlined.
