# Supplementary material for: Using an agent-based model to analyze the dynamic communication network of the immune response
Source: Theor Biol Med Model. 2011 Jan 19;8:1. doi: 10.1186/1742-4682-8-1 (PMC3032717; doi:10.1186/1742-4682-8-1)
Supplement: Additional file 7 — State diagram: Dendritic Agents (DCs) Zone 1. A state diagram of the potential DC behavioral sequences in Zone 1. [file 1742-4682-8-1-S7.PDF]

Additional file 7 - State diagram: Dendritic Agents (DCs) Zone 1.

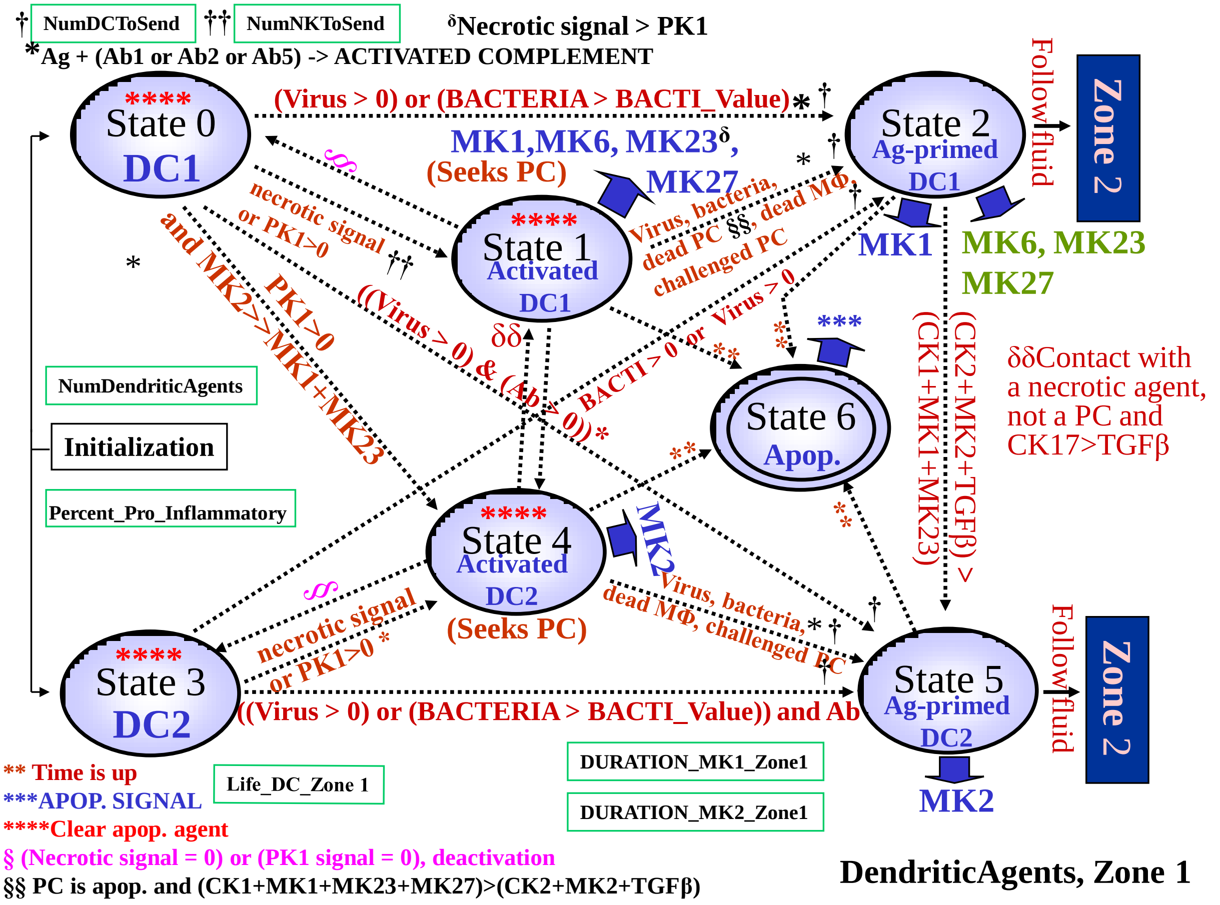

DendriticAgents, Zone 1

DCs begin in Zone 1, where they function in surveillance of the tissue for any disruption of the healthy state of the tissue [34]. The initial number of DCs in Zone 1 (200) approximates the number in 1 mm^2 of normal lung near the alveoli [125]. At initialization they may be in State 0 or State 3, depending on whether they have the potential to promote inflammation (DC1) or down-regulate it (DC2). The number of DCs and the ratio of DC1:DC2 is controlled by the input parameters numDendriticAgents and percentProInflammatory (additional File 4). These subsets are meant to represent the ability of dendritic cells to polarize the immune response [40, 41, 51]. Regardless of type, the immature DCs are able to ingest apoptotic agents [58].

When the simulation begins, the DCs migrate randomly in Zone 1, able to detect soluble stress factor (PK1), virus, antibodies (Ab1 or Ab2), and pro- and anti-inflammatory cytokines [Mono-kine 1 (MK1) and Mono-kine 2 (MK2), Cytokine 1 (CK1) and Cytokine 2 (CK2); see Table 1]. The DCs may transition to another state depending on which signal they encounter first. PK1 or necrotic signal causes the DCs to become activated [35] and transition to States 1 or 4. At this time NKs (Natural Killer Agents) enter Zone 1 as they would in response to cytokines [42], controlled by the input parameter numNKToSend. Once the DCs detect PK1 they follow its concentration gradient, seeking a challenged PC (Parenchymal Agent). In the activated state DC1s and DC2s also release signal, MK1, MK6[44], MK23[43], and MK27 [13] (DC1, depending on the stimulus) or MK2 [45] (DC2). At this point a DC1 may be induced to convert to the down-regulatory DC2 phenotype by the preponderance of MK2 signal already in the environment [7]. In addition to the transitions to the activated states, the presence of virus in the immediate environment causes the transition to the antigen-primed state (State 2 or 5) [37]. Viral antigen bound by antibody induces the transition of a DC1 to the DC2 type [39] and virus in combination with pro-inflammatory CK1 causes the transition of a DC2 to the pro-inflammatory, antigen-primed DC1 state [36]. Contact with virally infected PCs also causes activated DC1s and DC2s to transition to their respective antigen-primed states [34] and a new DC enters Zone 1 (numDCToSend).

If DC1s or DC2s reach the activated states (States 1 or 4) but do not detect soluble (signal) or PC-bound antigen within a pre-defined number of ticks they undergo apoptosis, or programmed cell death (State 6; LIFE_DC_Zone1) [60]. Once the DCs do make contact with antigen, they migrate to Zone 2 to present the antigen to the B Cell agents (Bs), Ts and Cytotoxic T Lymphocyte agents (CTLs) [34]. If the DC1 senses a preponderance of CK2 or detects apoptotic debris at the tick when the transition to Zone 2 is to be made, it will convert to a DC2 before migrating [7, 35].
