# Supplementary material for: Using an agent-based model to analyze the dynamic communication network of the immune response
Source: Theor Biol Med Model. 2011 Jan 19;8:1. doi: 10.1186/1742-4682-8-1 (PMC3032717; doi:10.1186/1742-4682-8-1)
Supplement: Additional file 8 — State diagram: Dendritic Agents (DCs), Zone 2. A state diagram of the potential DC behavioral sequences in Zone 2. [file 1742-4682-8-1-S8.PDF]

Additional file 8 - State diagram: Dendritic Agents (DCs), Zone 2.

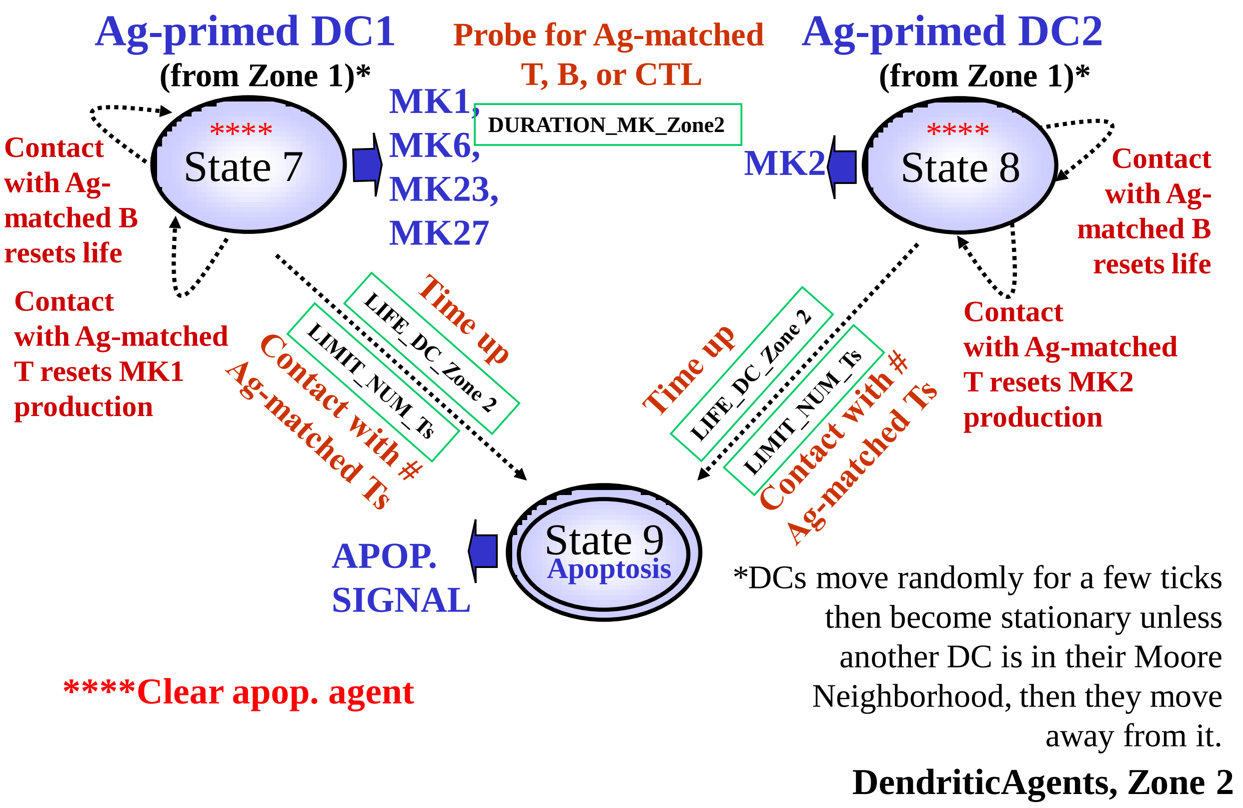

Once the DCs have migrated to Zone 2, they move randomly for a set number of ticks and then become stationary. They thereafter move only to maintain an evenly distributed web of DCs in Zone 2 [54]. While in Zone 2 they produce their respective cytokines and probe all of the B Cells Agents (Bs), T Cell Agents (Ts) and Cytotoxic T Lymphocytes (CTLs) that move randomly within their reach to look for an antigen-specificity matched agent [52]. All of the agents in Zone 2 have a pre-set specificity, and the number of ticks that will elapse before a matching agent is detected by a DC is by chance. When a match is found, the B, T or CTL becomes cognizant of the contact, affecting its state. The contact with an antigen-matched agent is noted by the DC, and it may extend the life of the DC [59], restart signal production or lead to the apoptosis of the DC if the number of T contacts reaches a threshold amount (LIMIT_NUM_Ts) [47, 55, 62].
