# Supplementary material for: Using an agent-based model to analyze the dynamic communication network of the immune response
Source: Theor Biol Med Model. 2011 Jan 19;8:1. doi: 10.1186/1742-4682-8-1 (PMC3032717; doi:10.1186/1742-4682-8-1)
Supplement: Additional file 9 — State diagram: Natural Killer Agents (NKs) in Zone 1. A state diagram of the potential NK behavioral sequences in Zone 1. [file 1742-4682-8-1-S9.PDF]

Additional file 9 - State diagram: Natural Killer Agents (NKs) in Zone 1.

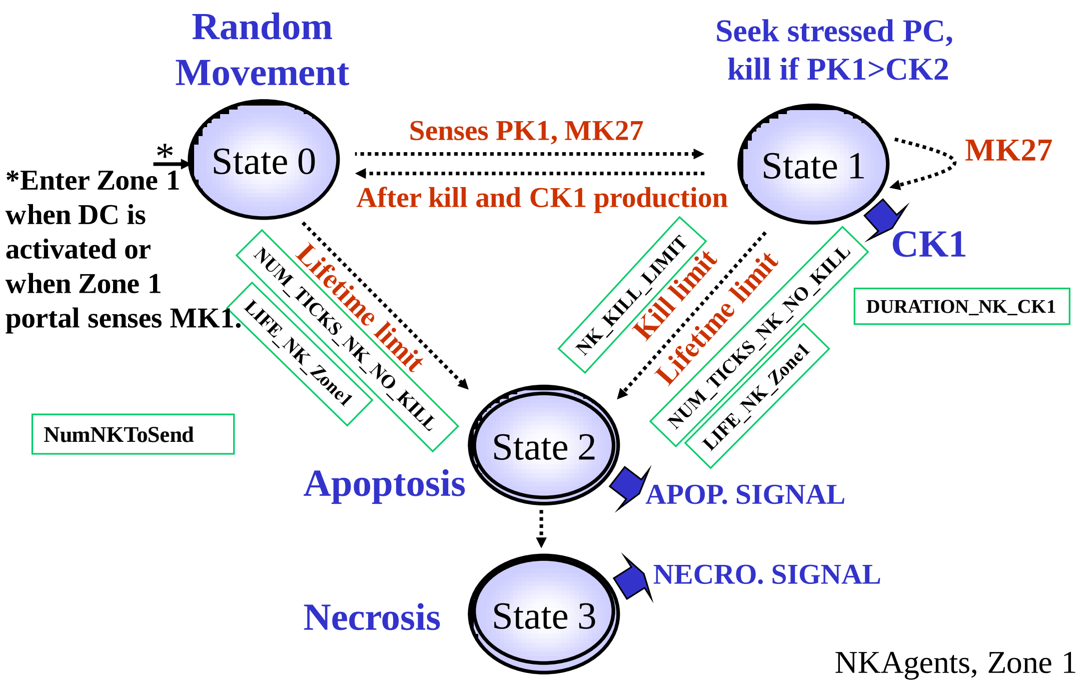

NKs enter Zone 1 in response to Dendritic Cell agent (DC) activation by parenchymalkine-1 (PK1) and production of (monokine-1) MK1. A Portal Agent in Zone 1 initially senses MK1, and sends in NKs (NumNKToSend), simulating a chemotactic response [2, 42, 102]. They move randomly until they sense PK1, then they transition to State 1 and follow the PK1 gradient to seek out any stressed Parenchymal Cell agents (PCs) that are producing it. They also produce cytokine-1 (CK1), a pro-inflammatory signal (representing IFN-γ) [97, 103]. The CK1 production is enhanced by MK27 [17]. Although NK cell recognition of self major histocompatibility complex (MHC) Class I on cells provides an inhibitory signal to prevent killing, in a pro-inflammatory environment [29] or if a cell is virally infected [105], the inhibition is overcome. If the NK finds a PC that is stressed and the (pro-inflammatory) PK1 signal present is greater than the CK2 signal, the PC is killed and the NK returns to State 0. The NKs have a limited lifetime (LIFE_NK_Zone1) and they have a limited number of kills that they may execute (NK_KILL_LIMIT) [101].
