# Supplementary material for: Using an agent-based model to analyze the dynamic communication network of the immune response
Source: Theor Biol Med Model. 2011 Jan 19;8:1. doi: 10.1186/1742-4682-8-1 (PMC3032717; doi:10.1186/1742-4682-8-1)
Supplement: Additional file 10 — State diagram: Macrophage agents (MΦs), Zone 1. A state diagram of the potential MΦ behavioral sequences in Zone 1. [file 1742-4682-8-1-S10.PDF]

Additional file 10 - State diagram: Macrophage agents (MΦs), Zone 1.

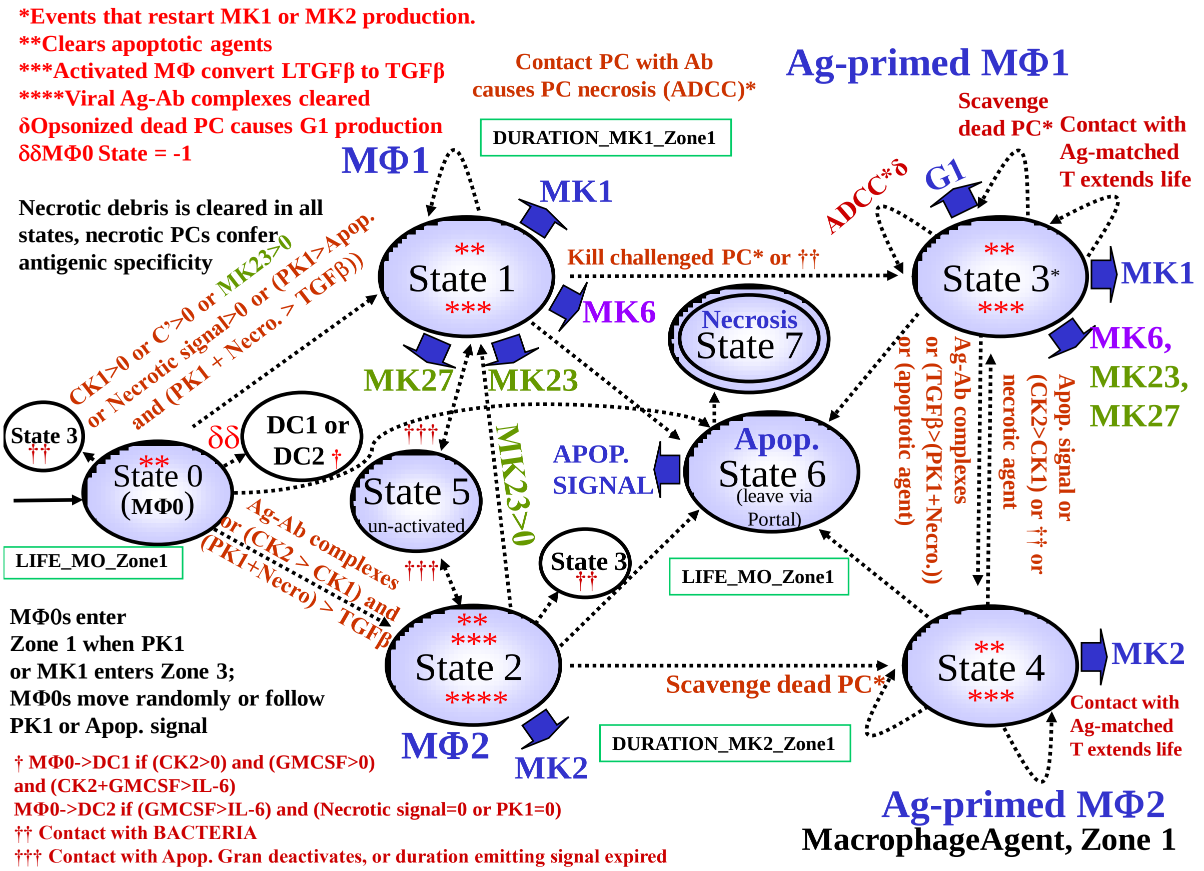

MΦs begin in Zone 1 as monocytes, or MΦ0s, and may differentiate into MΦ1s, MΦ2s, or Dendritic Agents [70]. MΦ0s also enter in response to “danger signals” [2, 64, 65], such as parenchymalkine-1 (PK1) or monokine-1 (MK1) that diffuse to Zone 3 via Portal Agents. The MΦs phagocytose apoptotic agents of all types [73]. The MΦ0s move randomly, and they follow signals in their immediate environment including PK1, cytokine-1 (CK1), complement (C’) [27], necrotic debris, apoptotic signal or antigen-antibody (Ag-Ab) complexes [30]. Sensing of any of the above signals causes the MΦs to transition to State 1 or State 2, depending on the signal [49, 63]. The transition to activated states causes the MΦ1s to produce the signals MK1, MK6 [69], MK23 and MK27 [72], and the MΦ2s to make MK2 [45]. Many cell types produce TGF-β in a latent form, and activated MΦs convert latent TGF-β (LTGFβ) to its active form [71] (TGFβ). Where TGFβ is shown to be produced in the state diagrams, this conversion is taking place where LTGFβ is available.

In activated states the MΦs have the ability to scavenge dead Parenchymal Agents (PCs), providing a necessary event for PC regeneration to occur and also allowing progression to State 3 or 4. A MΦ1 in State 1 may also kill a virally infected PC and transition to State 3, or kill an Ab2-opsonized PC, an event termed antibody-dependent cell cytotoxicity (ADCC) [30]. Progression to States 3 or 4 occurs when (implied) uptake and processing of antigen has taken place. The MΦs may make contact with T Cell agents (T1s or T2s) in States 3 and 4, extending the functional life of the antigen-specific Ts in the immune response. The pro-inflammatory MΦ1s may convert to anti-inflammatory MΦ2s if they detect a preponderance of CK2, TGFβ [66], Ag-Ab complexes [30, 49] or apoptotic signal above a threshold value [126]. The MΦs in the activated states (States 1-4) have a finite lifetime, and they undergo apoptosis when their time is up [75] (LIFE_MO_Zone1).
