# Supplementary material for: Using an agent-based model to analyze the dynamic communication network of the immune response
Source: Theor Biol Med Model. 2011 Jan 19;8:1. doi: 10.1186/1742-4682-8-1 (PMC3032717; doi:10.1186/1742-4682-8-1)
Supplement: Additional file 11 — State diagram: Macrophage Agents (MΦs) Zone 2. A state diagram of the potential MΦ behavioral sequences in Zone 2. [file 1742-4682-8-1-S11.PDF]

Additional file 11 - State diagram: Macrophage Agents (MΦs) Zone 2.

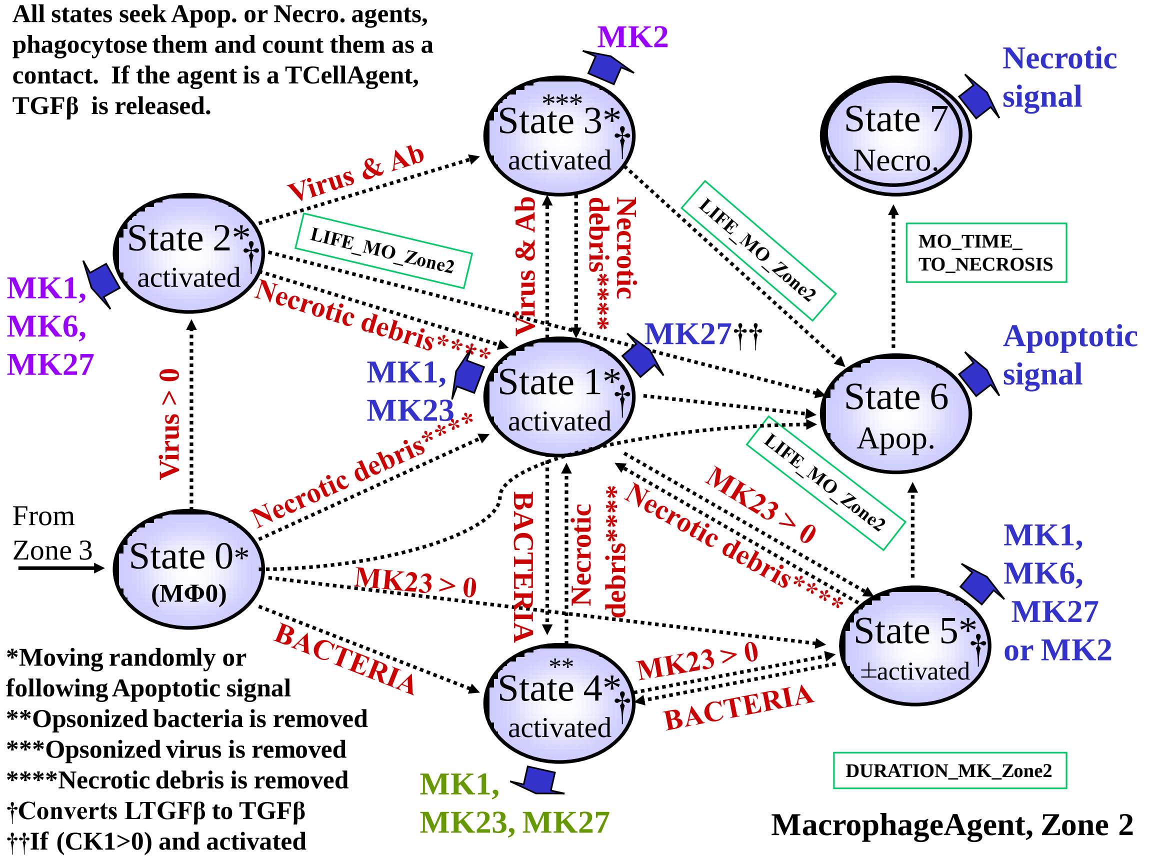

Macrophage Agents in Zone 2 begin in State 0 as monocytes, and they move randomly, seeking apoptotic or necrotic agents to phagocytose. They may change to another state (become activated) if they encounter any of the signals indicated between states. The pathogens programmed into the simulation, such as Virus and Bacteria, are able to migrate into Zone 2 (secondary lymphoid tissue) via Portal Agents, and they affect the behavior of agents in Zone 2 [67]. The conditions for changing from MΦ0 to pro-inflammatory MΦ1 or anti-inflammatory MΦ2 are essentially the same as in Zone 1.
