# Supplementary material for: Using an agent-based model to analyze the dynamic communication network of the immune response
Source: Theor Biol Med Model. 2011 Jan 19;8:1. doi: 10.1186/1742-4682-8-1 (PMC3032717; doi:10.1186/1742-4682-8-1)
Supplement: Additional file 12 — State diagram: Macrophage Agents (MΦs), Zone 3. A state diagram of the potential MΦ behavioral sequences in Zone 3. [file 1742-4682-8-1-S12.PDF]

Additional file 12 - State diagram: Macrophage Agents (MΦs), Zone 3.

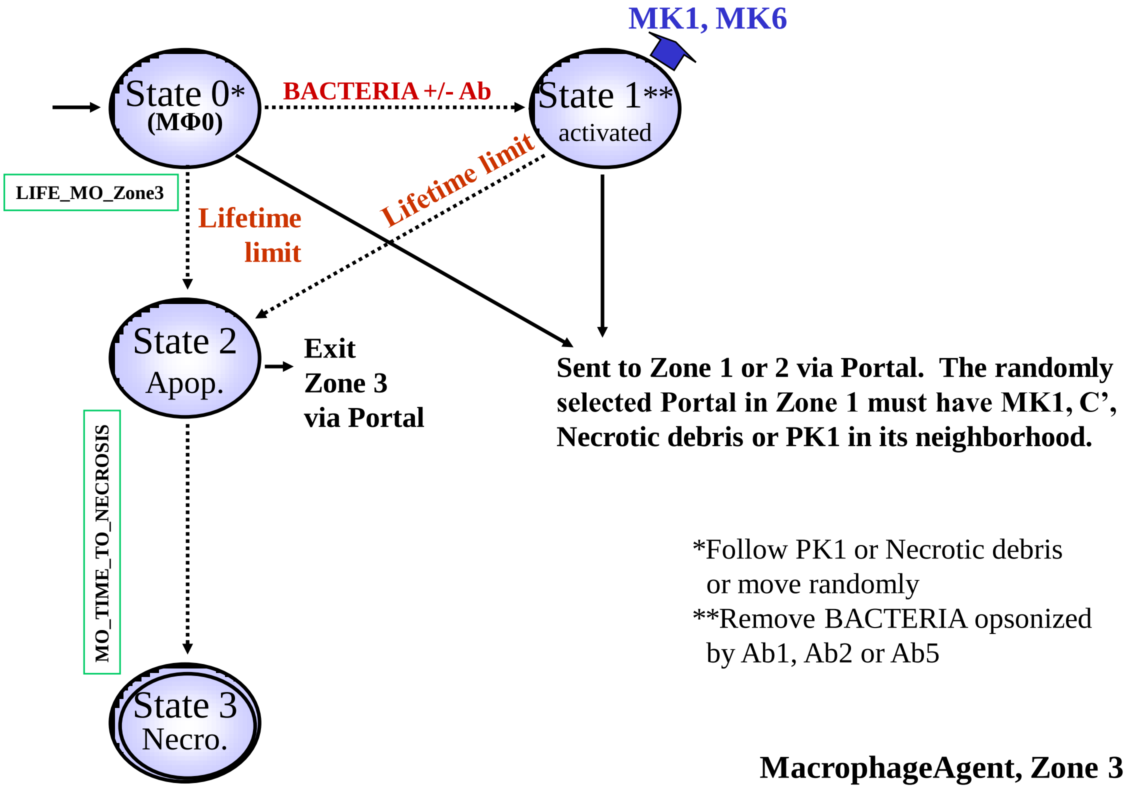

The Macrophage Agents in Zone 3 (the blood) are in the unactivated state, MΦ0 [70], unless they encounter bacteria or other pro-inflammatory signals. They may migrate to Zone 1 or Zone 2 if they encounter a Portal Agent in Zone 3, and that Portal Agent randomly selects a Portal Agent in Zones 1 or 2 that has chemotactic signals surrounding it such as MK1, PK1, activated complement[68] or necrotic debris[126]. The Macrophage Agents become activated by these signals and produce more pro-inflammatory signals [69].
