# Supplementary material for: Using an agent-based model to analyze the dynamic communication network of the immune response
Source: Theor Biol Med Model. 2011 Jan 19;8:1. doi: 10.1186/1742-4682-8-1 (PMC3032717; doi:10.1186/1742-4682-8-1)
Supplement: Additional file 13 — State diagram: BCell Agents (Bs) in Zone 2 (Part 1). A state diagram of the potential B behavioral sequences in Zone 2. [file 1742-4682-8-1-S13.PDF]

Additional file 13 - State diagram: BCell Agents (Bs) in Zone 2 (Part 1)

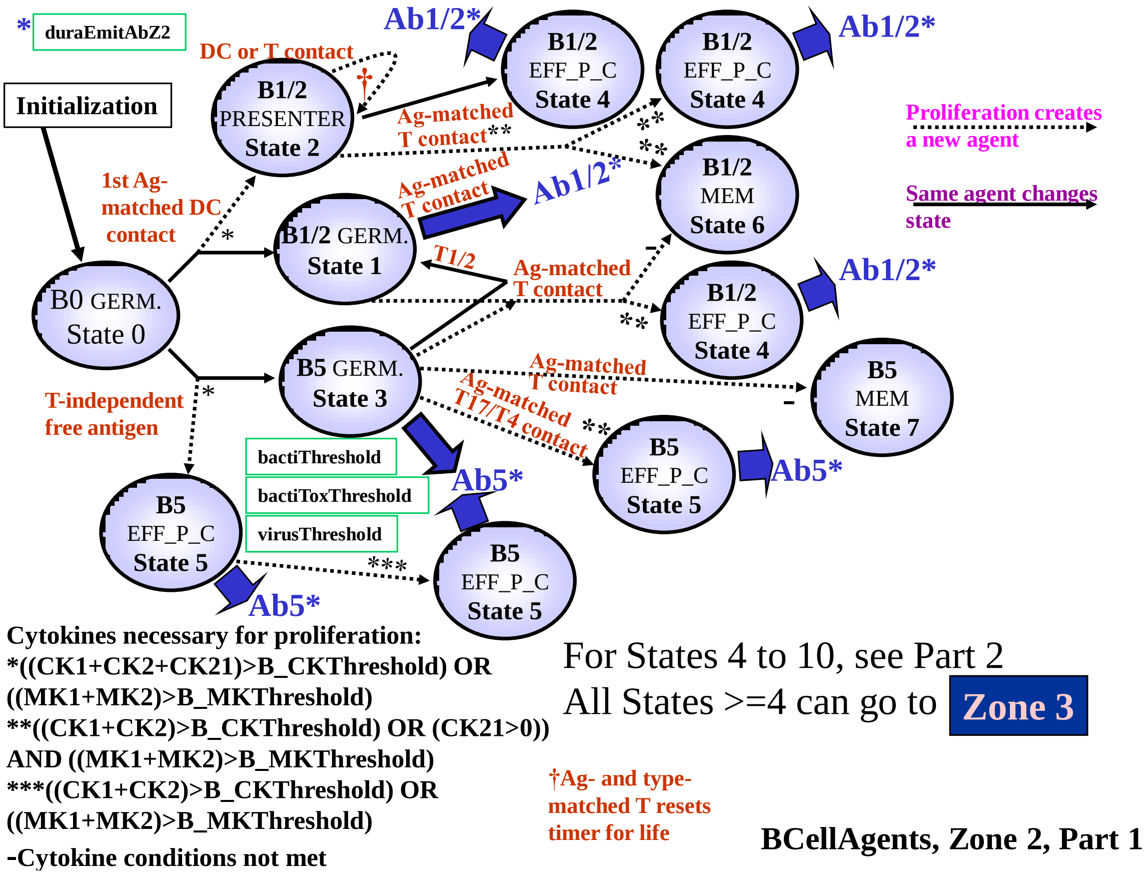

Bs begin in a germinal state in Zone 2. They require contact with antigen (Ag) [67] or a Dendritic Agent (DC) that is presenting antigen that matches their pre-set specificity [46]. The fraction of Bs that is specific for any particular antigen is an input parameter to the simulation (PercentBAntiViral). The default fraction of virally specific Bs that was used for these experiments was 0.67% (10/1500; additional file 4).

The DC contributes to the response of the B via contact and cytokines [46, 110]. If presentation of antigen is made by a DC1, the B will be type B1 and make antibody-1 (Ab1). Presentation by a DC2 will cause the B to become a B2 and make Ab2 (additional file 1). The abbreviations B1 and B2 indicate different populations present in the simulation and are not meant to correspond to such designations in the literature for B lymphocytes [116]. If the germinal B detects free antigen in Zone 2, it becomes a B5, representing the type of B-lymphocyte that makes IgM [109]. Cytokine stimulation is necessary for proliferation [46, 107], and some of the Bs remain in the germinal center of the lymph node (States 0,1 and 3).

Once the B has either seen soluble Ag (such as virus) [67] or seen Ag presented by a DC, the B requires cytokine stimulation [107, 108] and contact with an antigen matched T1 or T2 before it can produce Ab (States 1 and 3-5) [87, 89]. Once the B makes contact with an activated, Ag-matched T, it proceeds to become an effector plasma cell (EFF_P_C; States 4 or 5), produces antibody and becomes able to travel to Zone 3. Specific T contact also causes a B to proliferate, the number of progeny produced at each contact is an input parameter to the simulation (NumB_ToSend). A sufficient number of contacts in the absence of cytokine [114, 115] results in the formation of a long-lived memory B (States 6,7). A memory B may be re-activated to produce Ab by the contact of an Ag-specific T or DC [110] and the presence of cytokines in Zone 2.
