# Supplementary material for: Using an agent-based model to analyze the dynamic communication network of the immune response
Source: Theor Biol Med Model. 2011 Jan 19;8:1. doi: 10.1186/1742-4682-8-1 (PMC3032717; doi:10.1186/1742-4682-8-1)
Supplement: Additional file 14 — State diagram: BCell Agents (Bs) in Zone 2 (Part 2). A state diagram of the potential B behavioral sequences in Zone 2. [file 1742-4682-8-1-S14.PDF]

Additional file 14 - State diagram: BCell Agents (Bs) in Zone 2 (Part 2)

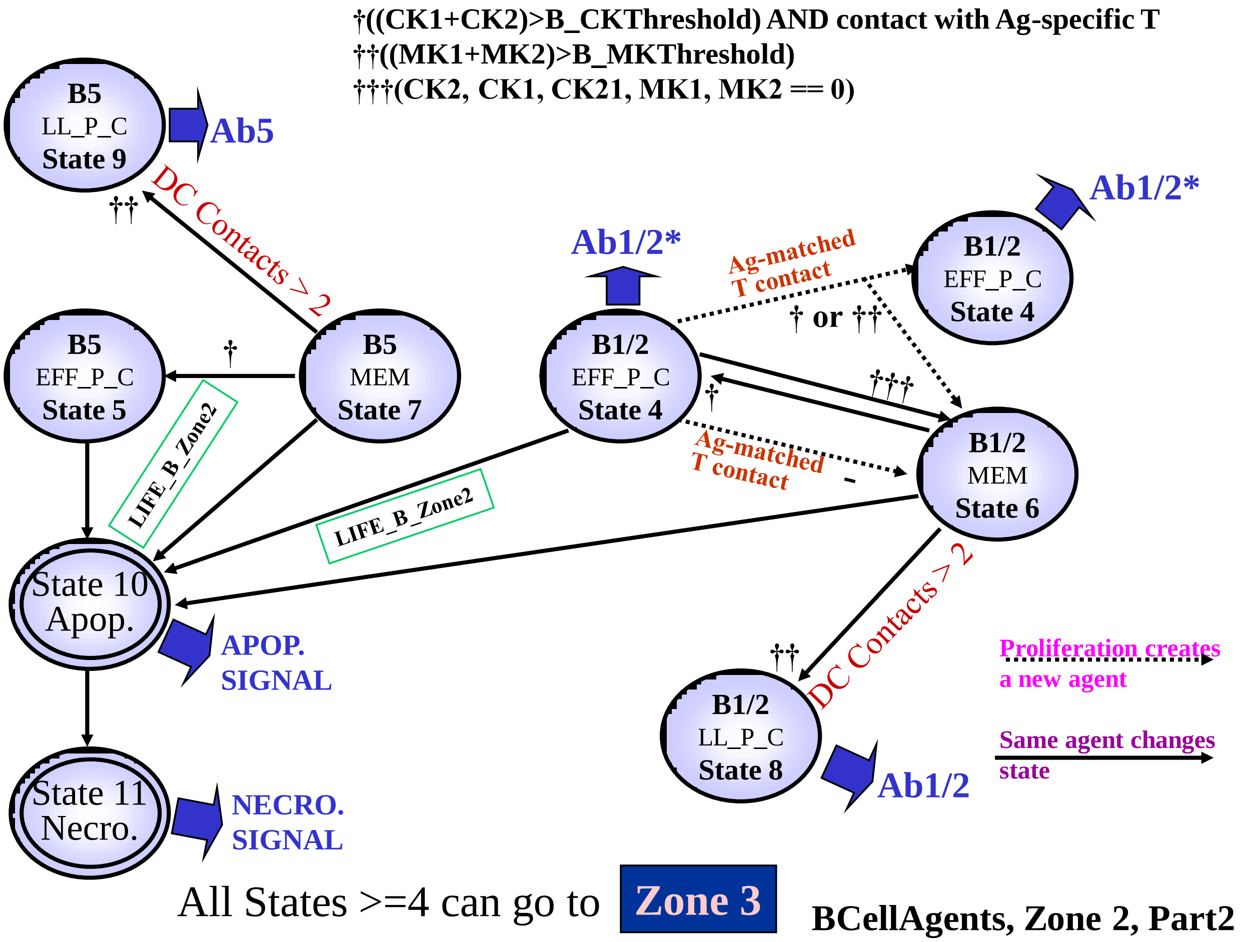

B-lymphocytes have many stages in their development, because there must be a rapid initial antibody response to pathogens (EFF_P_C), and a long lived memory response with low levels of antibody in the blood (long-lived plasma cells, LL_P_C) [112, 113].
