# Supplementary material for: Using an agent-based model to analyze the dynamic communication network of the immune response
Source: Theor Biol Med Model. 2011 Jan 19;8:1. doi: 10.1186/1742-4682-8-1 (PMC3032717; doi:10.1186/1742-4682-8-1)
Supplement: Additional file 15 — State diagram: BCell Agents (Bs) in Zones 3 and 1. A state diagram of the potential B behavioral sequences in Zones 3 and 1. [file 1742-4682-8-1-S15.PDF]

Additional file 15 - State diagram: BCell Agents (Bs) in Zones 3 and 1

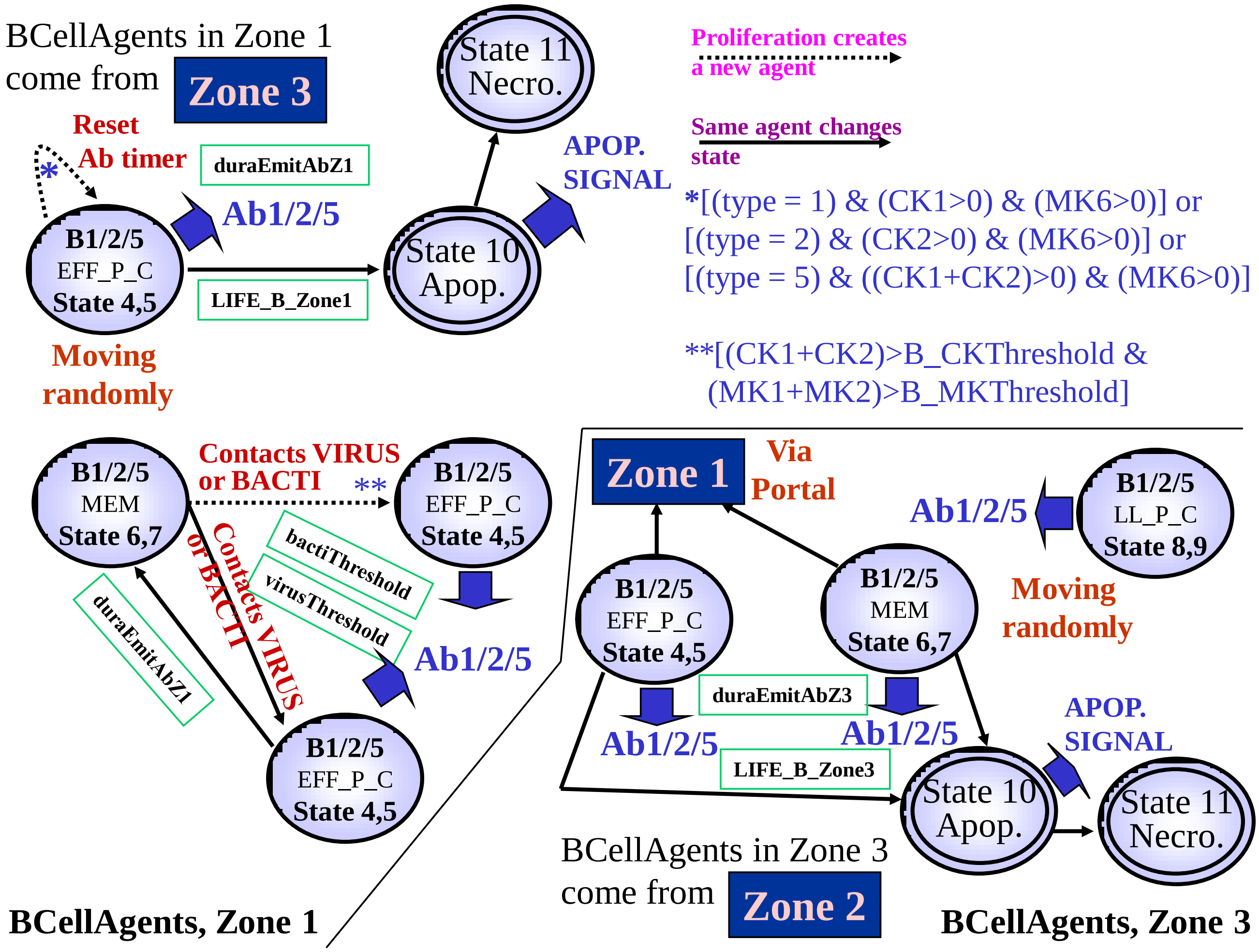

Activated Bs in States 4 or 5 represent plasma cells and migrate to Zone 3 (the blood) where they produce antibody (Ab) that diffuses into Zone 1, and they may migrate into the actual site of inflammation, Zone 1, from there [111]. As long as they remain in Zone 3 they move randomly. The EFF_P_C (States 4, 5) Bs have a finite lifetime (LIFE_B_Zone3) and a finite period of time to produce Ab in Zone 3 (duraEmitAbZ3) controlled by an input parameter.

In Zone 1 the Bs continue to move randomly and produce antibody (Ab) if cytokine-1 (CK1) or CK2 is present (States 4,5; duraEmitAbZ1) [106]. Once the signal is no longer present they cease to produce Ab within a period of time (State 9). This represents the behavior of B lymphocytes in immune responses where they may be found in sites of tissue inflammation [111]. Bs have a finite lifetime in Zone 1 determined by an input parameter (additional file 4; LIFE_B_Zone1).
