# Supplementary material for: Using an agent-based model to analyze the dynamic communication network of the immune response
Source: Theor Biol Med Model. 2011 Jan 19;8:1. doi: 10.1186/1742-4682-8-1 (PMC3032717; doi:10.1186/1742-4682-8-1)
Supplement: Additional file 16 — State diagram: TCell Agents (Ts) in Zone 2 (Part 1). A state diagram of the potential T behavioral sequences in Zone 2. [file 1742-4682-8-1-S16.PDF]

Additional File 16 - State diagram: TCell Agents (Ts) in Zone 2 (Part 1)

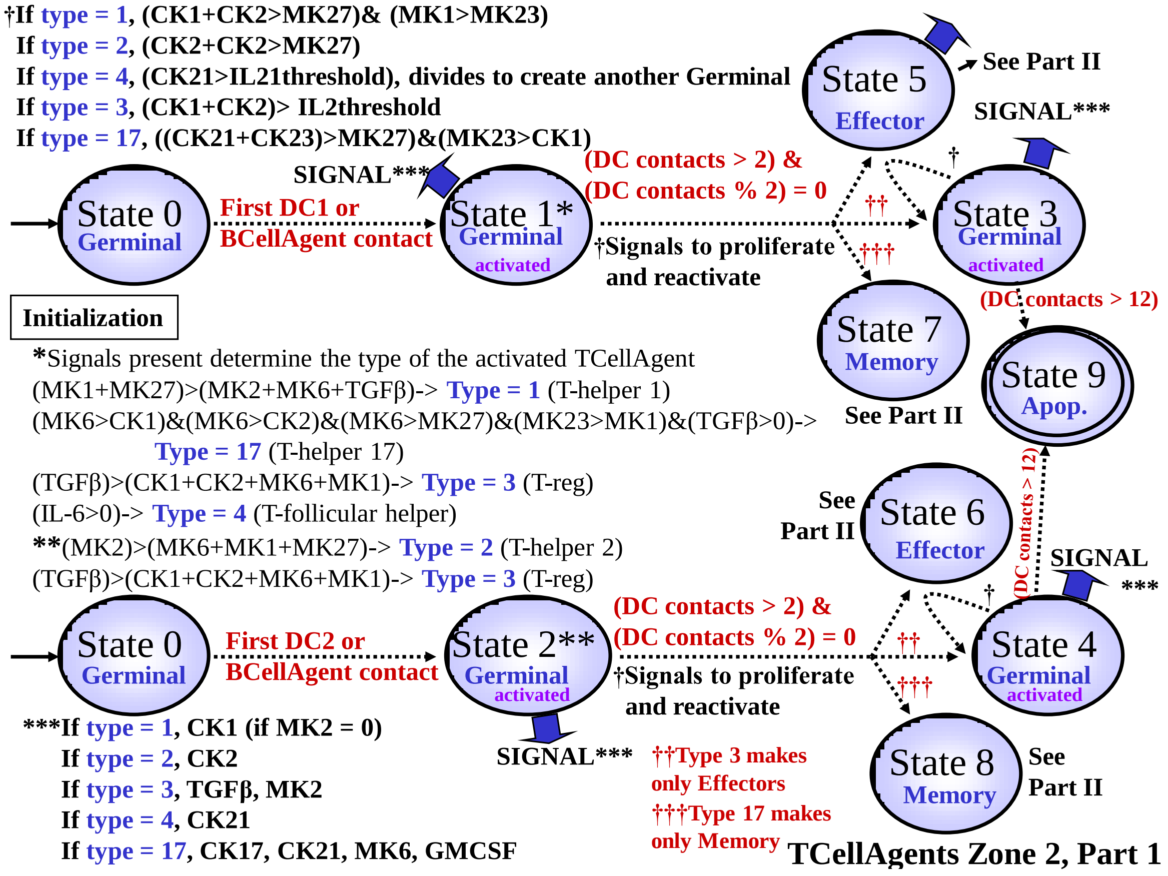

The Ts in the simulation represent lymphocytes of the CD4+, T-helper type. The initial fraction of Ts that are specific for any given antigen is an input parameter to the simulation, and the default value is 0.4% (additional file 4; PercentTAntiViral). The Ts begin in Zone 2 in an inactive, germinal state, moving randomly [52] and waiting to make contact with an antigen-matched Dendritic Agents (DCs) [34, 41, 47, 51] or BCell Agents [89] (Bs). The pro-inflammatory (DC1) or alternatively activated (DC2) type of the DC that makes first contact with the T and the predominant signals (cytokines) present determines the type of response that the T will engage in for the remainder of the simulation. Five types of T-helper lymphocytes are now represented in the BIS_2010. Types 1 and 2 have been described [127], and T-helper-17s, T-regs and T-follicular helpers have been added. The predominant presence of MK1 [76, 77] (IL-12) and MK27 [79] (IL-27) allows differentiation to T1; MK2 (IL-10, IL-4) promotes differentaition to T2 [39, 49, 78]; MK23 [12, 16] (IL-23), MK6 [8, 9, 11] (IL-6), and TGFβ [8, 9, 20, 22] (TGF-β) together promote differentiation to T17; TGFβ is necessary for differentiation to T3 [128] (T-reg); and the presence of MK6 along with none of the conditions for any of the other types fulfilled allows differentiation to the T4 [44, 129] (T-follicular helper) type.

Each of the different TCell Agent types has a particular cytokine(s) that it produces that distinguishes it from the other types. The cytokines produced by each T type are listed in Table 1 next to the type in red font, with references.

The initial contacts between T-lymphocytes (both T-helper and cytotoxic T lymphocytes) and dendritic cells have been observed and recorded with various multi-colored label, tissue penetrating, and time lapse photography methods [57]. Multiple contacts have been observed between T lymphocytes and dendritic cells, with short initial encounters and a final long encounter that results in T lymphocyte proliferation [90]. For this reason, multiple TCell Agent-Dendritic Agent contacts were programmed into the BIS_2010. The antigen-specific, prolonged contact between a naive T lymphocyte and a dendritic cell that results in cell division has been found to have a special property called "asymmetric division" [18]. For both types of T lymphocytes (T-helper and cytotoxic T lymphocytes), the first cell division occurs while the T cell is attached to the dendritic cell. The daughter T cell proximal to the dendritic cell becomes an "effector" cell, and the distal daughter cell becomes a "memory" cell. The division of TCell Agents after multiple contacts with Dendritic Agents is programmed to produce "daughter" agents in these two different states.
