# Supplementary material for: Using an agent-based model to analyze the dynamic communication network of the immune response
Source: Theor Biol Med Model. 2011 Jan 19;8:1. doi: 10.1186/1742-4682-8-1 (PMC3032717; doi:10.1186/1742-4682-8-1)
Supplement: Additional file 17 — State diagram: TCell Agents (Ts) in Zone 2 (Part 2). A state diagram of the potential T behavioral sequences in Zone 2 [file 1742-4682-8-1-S17.PDF]

Additional file 17 - State diagram: TCell Agents (Ts) in Zone 2 (Part 2)

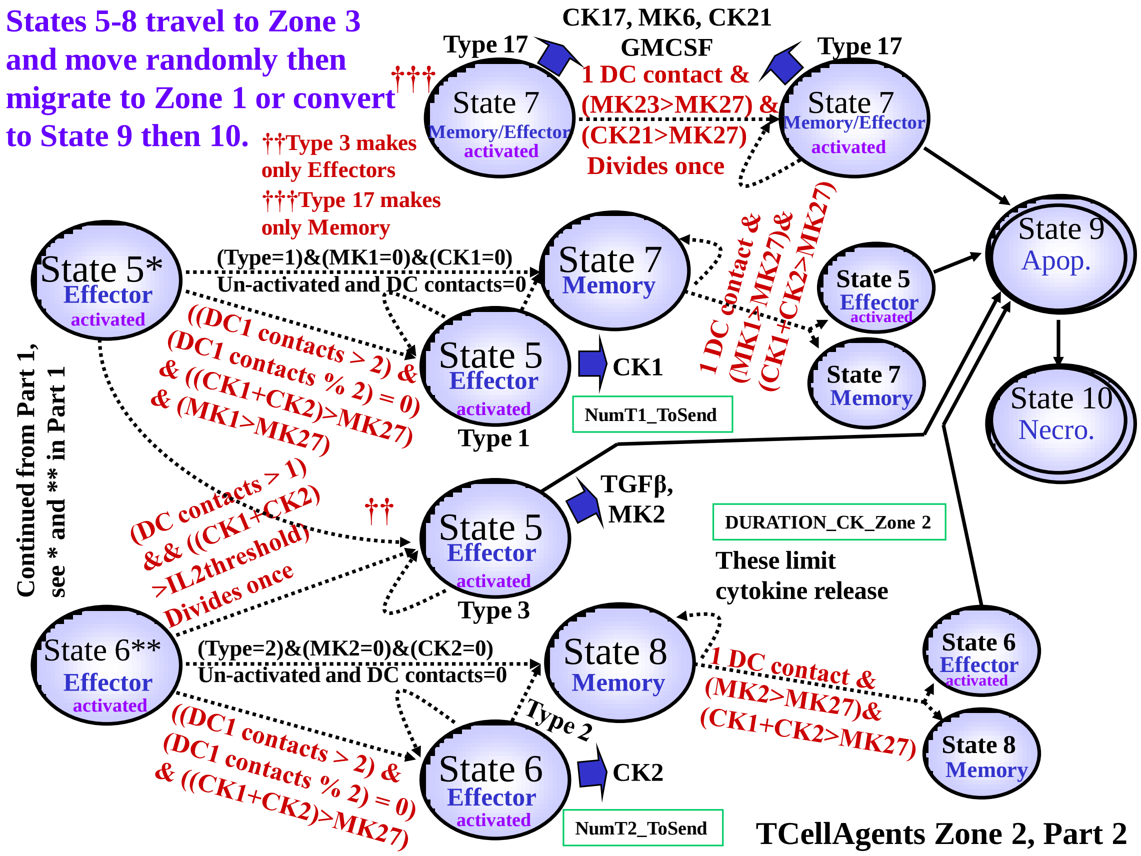

Once contact and differentiation has occurred, the Ts require signals to proliferate. The cytokine required for T lymphocytes (T1, T2, and T-reg) to proliferate is IL-2 [80], and CK1 together with CK2 is used to represent IL-2 in the BIS_2010. T follicular helper cells need IL-21 [85], and T17s need IL-21 and IL-23 [12, 21, 83]. The proliferation of all of the T helper subtypes is suppressed by IL-27 after they are activated or in the memory state [79]. Memory Ts monitor the presence of cytokines in their immediate environment. Absence of cytokines in the environment allows them to transition to long-lived memory Ts [82]. Contact with an antigen- and response type- matched DC brings memory Ts back to the activated state [88]. In the absence of DC contact in Zone 2 activated Ts may undergo apoptosis [59]. T lymphocytes have a finite lifetime [92], except for those in the memory state. Ts migrate to Zone 3, where they move randomly until they migrate into Zone 1.
