# Supplementary material for: Using an agent-based model to analyze the dynamic communication network of the immune response
Source: Theor Biol Med Model. 2011 Jan 19;8:1. doi: 10.1186/1742-4682-8-1 (PMC3032717; doi:10.1186/1742-4682-8-1)
Supplement: Additional file 18 — State diagram: T Cell agents (Ts) in Zone 1. A state diagram of the potential T behavioral sequences in Zone 1. [file 1742-4682-8-1-S18.PDF]

Additional file 18 - State diagram: T Cell agents (Ts) in Zone 1

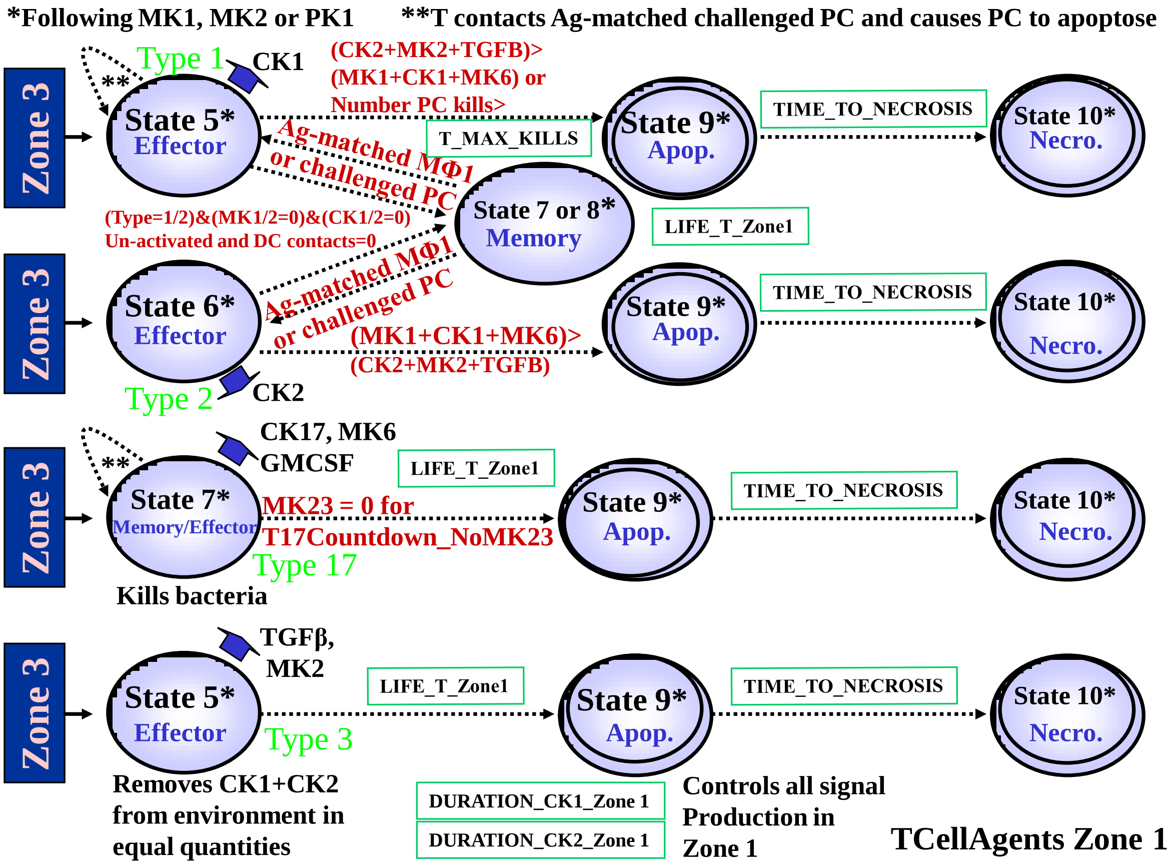

When Ts arrive in Zone 1 they may follow monokine-1 (MK1), MK2 or parenchymalkine-1 (PK1), whichever signal they find to be the strongest in their immediate vicinity. If none are present they move randomly until they encounter a signal. Detection of an antigen-matched Macrophage Agent (MΦ1) by a T causes it to emit CK1 or CK2 [49]. They also probe their immediate environment for a virally infected Parenchymal Agent (PC), which they kill upon encounter. The T1s count the number of PCs that they kill, because they may only kill a finite number of times before undergoing apoptosis themselves (T_MAX_KILLS) [28]. The relative quantities of cytokines control when the Ts undergo apoptosis [91, 93, 94]. T-regs have the unique ability to remove IL-2 (CK1+CK2) from the environment [84]. Otherwise Ts remain in Zone 1 until they run out of time and undergo apoptosis (LIFE_T_ZONE1) [28].
