# Supplementary material for: Using an agent-based model to analyze the dynamic communication network of the immune response
Source: Theor Biol Med Model. 2011 Jan 19;8:1. doi: 10.1186/1742-4682-8-1 (PMC3032717; doi:10.1186/1742-4682-8-1)
Supplement: Additional file 19 — State diagram: Cytotoxic T Lymphocyte Agents (CTLs) in Zones 2 and 3. A state diagram of the potential CTL behavioral sequences in Zones 2 and 3. [file 1742-4682-8-1-S19.PDF]

Additional file 19 - State diagram: Cytotoxic T Lymphocyte Agents (CTLs) in Zones 2 and 3

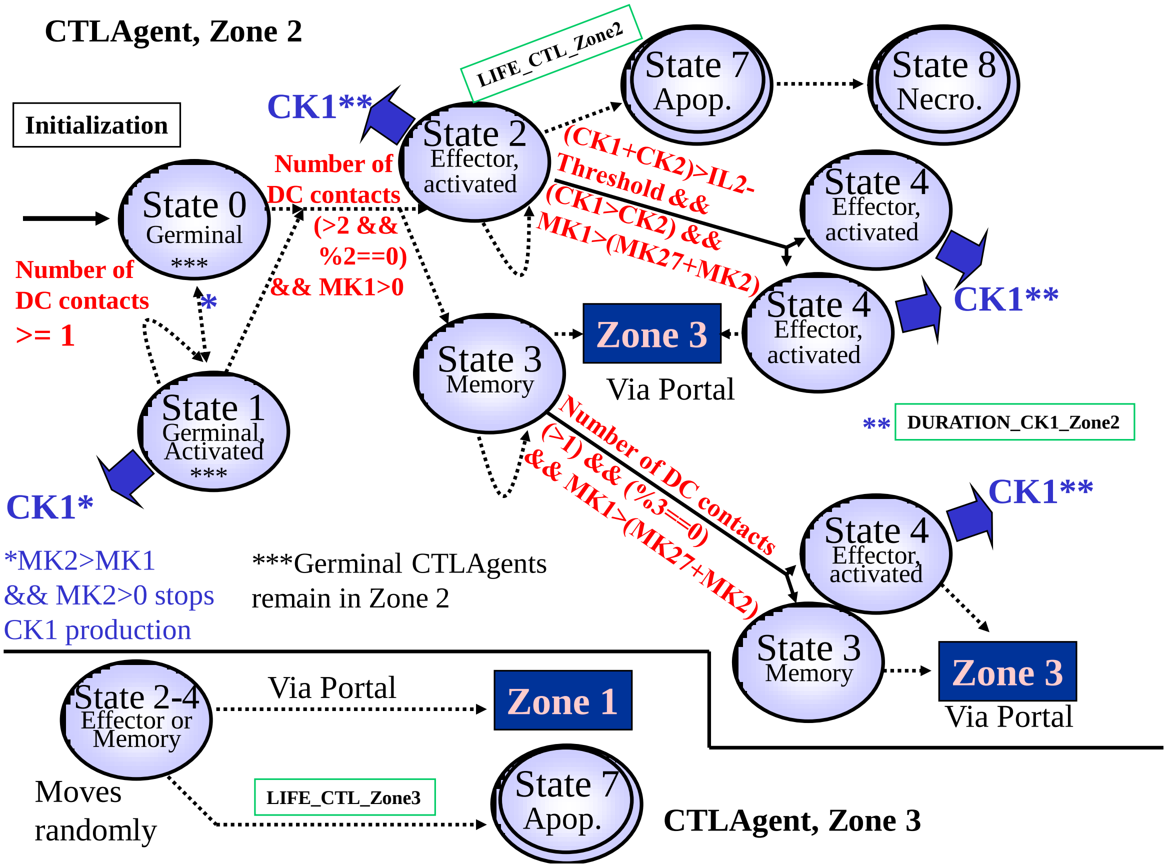

The CTLs begin in a resting state in Zone 2. The initial percentage of virus-specific CTLs is 0.4% (additional file 4; PercentCTLAntiViral). They move randomly and wait for contact with an antigen-specific Dendritic Cell agent type 1 (DC1) in order to become activated [99]. This process is referred to as “cross-priming” or “cross-presentation” [53]. CTLs undergo asymmetric division after sufficient contacts with a DC [18]. Effector CTLs produce CK1 (IFN-γ), the presence of cytokine (IL-2) leads to proliferation of the CTLs [80], and the progeny migrate from the blood to the tissue to seek and kill infected cells [100].
