# Supplementary material for: Using an agent-based model to analyze the dynamic communication network of the immune response
Source: Theor Biol Med Model. 2011 Jan 19;8:1. doi: 10.1186/1742-4682-8-1 (PMC3032717; doi:10.1186/1742-4682-8-1)
Supplement: Additional file 20 — State diagram: Cytotoxic T Lymphocyte Agents (CTLs) in Zone 1. A state diagram of the potential CTL behavioral sequences in Zone 1. [file 1742-4682-8-1-S20.PDF]

Additional file 20 - State diagram: Cytotoxic T Lymphocyte Agents (CTLs) in Zone 1

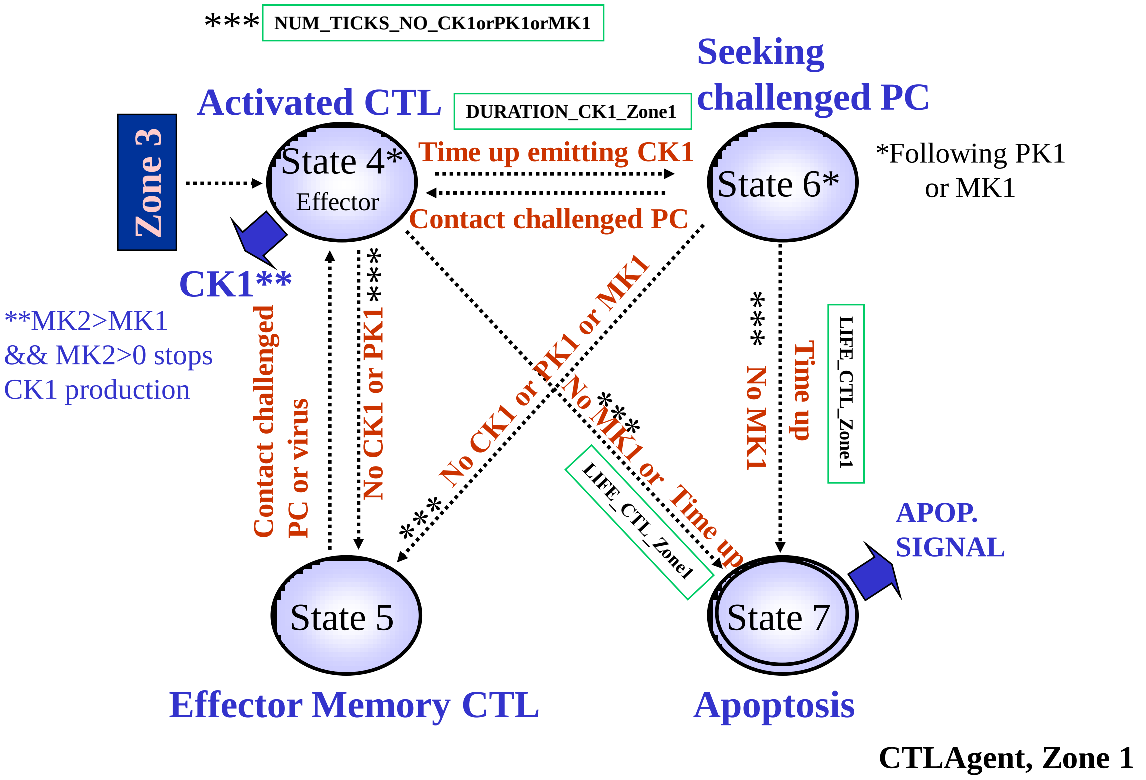

CTLs function to protect the body from intracellular pathogens, and they carry out this function by killing the infected, self, cells while in contact with them [98]. The CTLs migrate from Zone 3 into Zone 1 where they enter as activated, cytokine-1 (CK1)-producing agents. They produce CK1 for a finite period of time (DURATION_CK1_Zone1) that may be extended by contact with a virally infected Parenchymal Agent (PC). They continually sense CK1 and parenchymalkine-1 (PK1) in their immediate environment and follow PK1 to seek infected PCs, that they kill upon contact. In the absence of any cytokine for a defined period of time they may become effector memory CTLs (State 6), a state that allows them to persist for a long period of time and from which they may become activated (back to State 4) by an encounter with a virally infected PC [95, 96].
