# Supplementary material for: Using an agent-based model to analyze the dynamic communication network of the immune response
Source: Theor Biol Med Model. 2011 Jan 19;8:1. doi: 10.1186/1742-4682-8-1 (PMC3032717; doi:10.1186/1742-4682-8-1)
Supplement: Additional file 21 — State diagram: Granulocyte Agents (GRAN) in Zones 3, 1. A state diagram of the potential GRAN behavioral sequences in Zones 3 and 1. [file 1742-4682-8-1-S21.PDF]

Additional file 21 - State diagram: Granulocyte Agents (GRAN) in Zones 3, 1

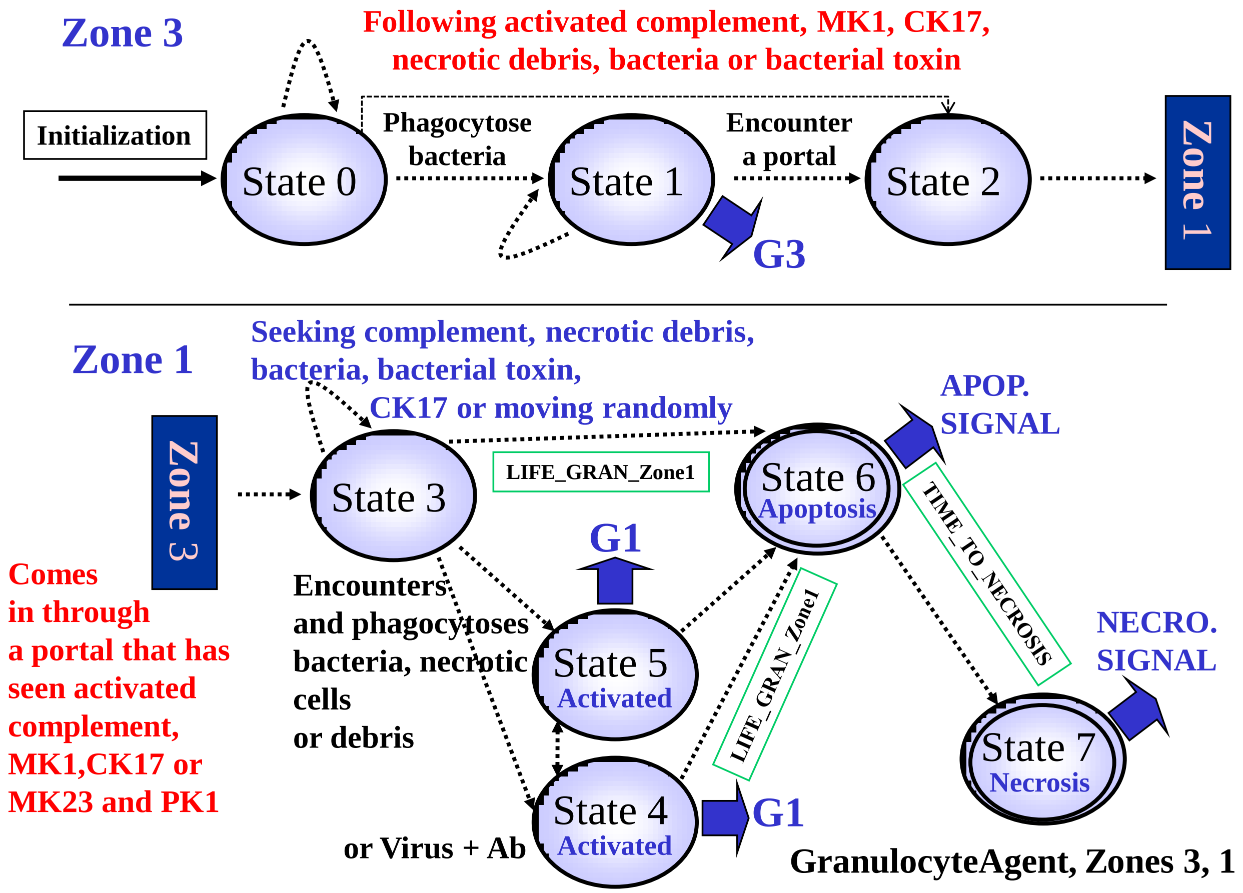

Granulocyte agents begin in Zone 3, moving randomly until they detect a portal emitting complement products [27], monokine-1 (MK1) [118, 119], CK17 (IL-17) [23, 117] or PK1 [121]. At the detection of this signal they migrate into Zone 1 where they may follow the same signals including complement products, necrotic debris [121], bacteria or bacterial toxin [120], or CK17, or move randomly. They have a short lifetime in Zone 1 (LIFE_Gran_Zone1), during which they emit a degranulation signal that is lethal for any stressed Parenchymal Agent (PC) that encounters it [31, 122]. When their lifetime is expended, neutrophils undergo apoptosis [123] then necrosis [124].
