# Supplementary material for: Using an agent-based model to analyze the dynamic communication network of the immune response
Source: Theor Biol Med Model. 2011 Jan 19;8:1. doi: 10.1186/1742-4682-8-1 (PMC3032717; doi:10.1186/1742-4682-8-1)
Supplement: Additional file 23 — Percentage of win or loss outcomes and ticks to eliminate infected agents for different starting conditions. A figure showing the outcomes when the initial number of Dendritic Agents is varied. [file 1742-4682-8-1-S23.PDF]

Additional file 23 – Percentage of *win* or *loss* outcomes and ticks to eliminate infected agents for different starting conditions

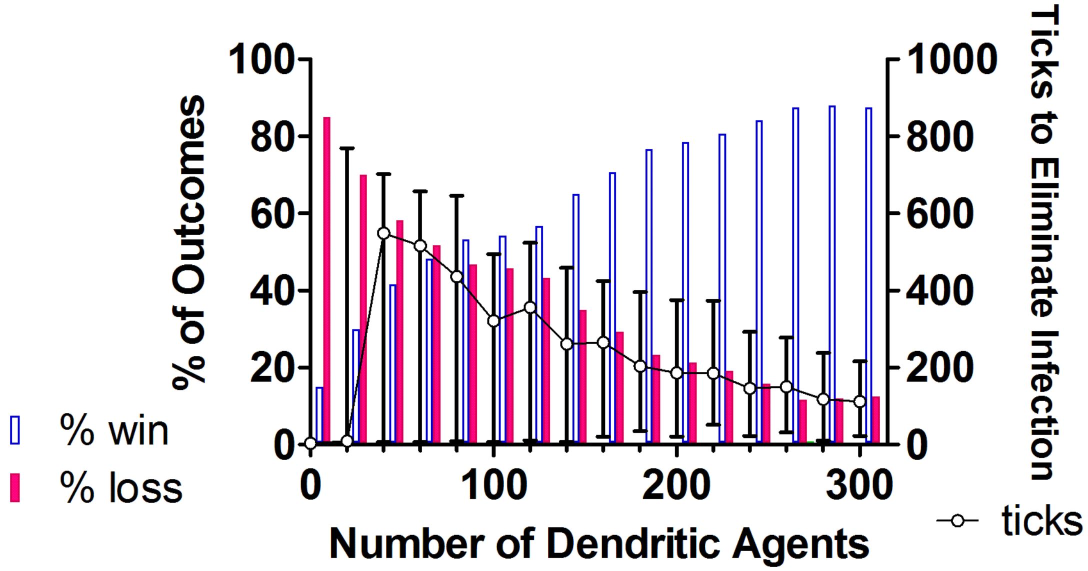

The initial parameter value for the numbers of Dendritic Agents in Zone 1 at initialization was subjected to a parameter sweep and the percentage of *win* vs. *loss* outcomes were determined and plotted using the left Y-axis (*win*, empty blue bars; *loss*, pink solid bars). For the win outcomes, the median number of ticks that had passed when the infected Parenchymal Agents were eliminated is shown on the right Y-axis, with an empty circle with error bars that indicate the 25th and 75th percentile. The number of simulation runs for each starting condition was n = 120 except for 160 Dendritic Agents (n = 116); 260 Dendritic Agents (n = 116); and 280 Dendritic Agents (n = 108). The data shown above were collected separately from the data shown in Figures 2, 3, 4, 5, 6, 7, 8 and additional files 24, 25, 26, 27, 28, 29, 31, 32, and 33.
