# Supplementary material for: Using an agent-based model to analyze the dynamic communication network of the immune response
Source: Theor Biol Med Model. 2011 Jan 19;8:1. doi: 10.1186/1742-4682-8-1 (PMC3032717; doi:10.1186/1742-4682-8-1)
Supplement: Additional file 24 — The number of Effector and Memory TCell Agents in Zone 1 for the duration of the simulation for the win and loss outcomes. A figure that shows the average numbers of TCell Agents in Zone 1 for the duration of the simulation. [file 1742-4682-8-1-S24.PDF]

Additional file 24 - The number of Effector and Memory TCell Agents in Zone 1 for the duration of the simulation for the *win* and *loss* outcomes.

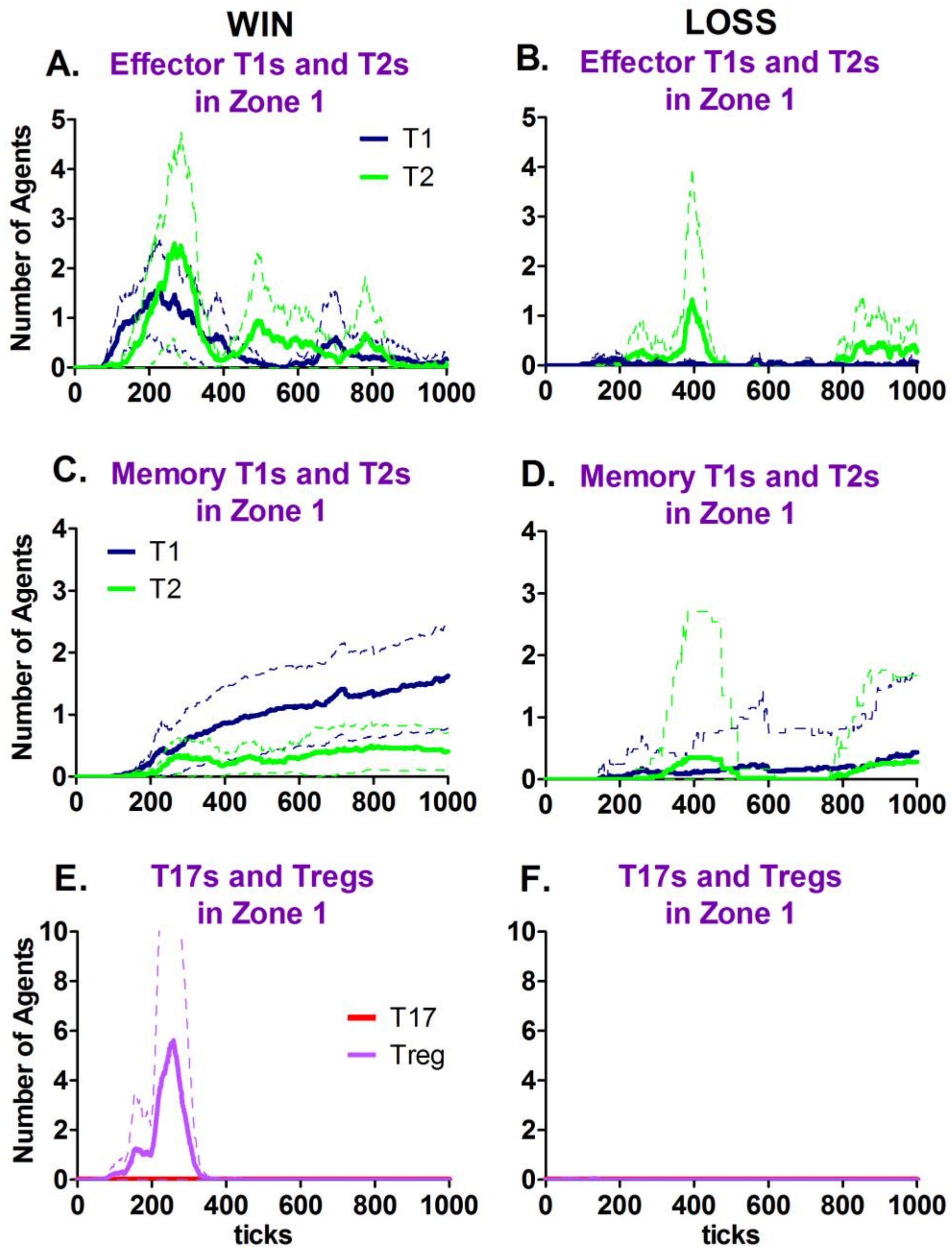

A. and B. The average number of Effector TCell Agents of types T1 (T-helper 1, blue) and T2 (T-helper 2, green) ± the 95% confidence interval (solid line and dashed lines, respectively) for the *win* (A, n = 100) and *loss* (B, n = 46) outcomes is shown.
C. and D. The average number of Memory TCell Agents of types T1 (blue) and T2 (green) ± the 95% confidence interval (solid line and dashed lines, respectively) for the *win* (C, n = 100) and *loss* (D, n = 46) outcomes is shown.
E. and F. The average number of TCell Agents of types T17 (T-helper 17, red) and Treg (T-regulatory, violet) ± the 95% confidence interval (solid line and dashed lines, respectively) for the *win* (E, n = 100) and *loss* (F, n = 46) outcomes is shown.

The migration of dendritic cells to the lymph node is necessary for activation of the various types of lymphocytes by antigen presentation [130-133]. The result is activation and proliferation of the lymphocytes, which must then travel back to the infected tissue via the blood (additional files 13, 14, 15, 16, 17, 18, 19, and 20). Corroborating the contact data in Table 2, the numbers of agents representing T-helper subtypes that migrated back to Zone 1 are greater in the *win* outcomes than in the *loss* outcomes (A, C, and E vs. B, D, and F). T-helper 17s are lymphocytes thought to be necessary for anti-bacterial responses and are associated with auto-immune reactions [117, 134, 135]. Their minimal participation in the virtual anti-viral response is valid.
