# Supplementary material for: Using an agent-based model to analyze the dynamic communication network of the immune response
Source: Theor Biol Med Model. 2011 Jan 19;8:1. doi: 10.1186/1742-4682-8-1 (PMC3032717; doi:10.1186/1742-4682-8-1)
Supplement: Additional file 25 — The number of Effector and Memory BCell Agents in Zone 1 for the duration of the simulation for the win and loss outcomes. A figure that shows the average numbers of Effector and Memory BCell Agents in Zone 1 for the duration of the simulation. [file 1742-4682-8-1-S25.PDF]

Additional file 25 - The number of Effector and Memory BCell Agents in Zone 1 for the duration of the simulation for the *win* and *loss* outcomes.

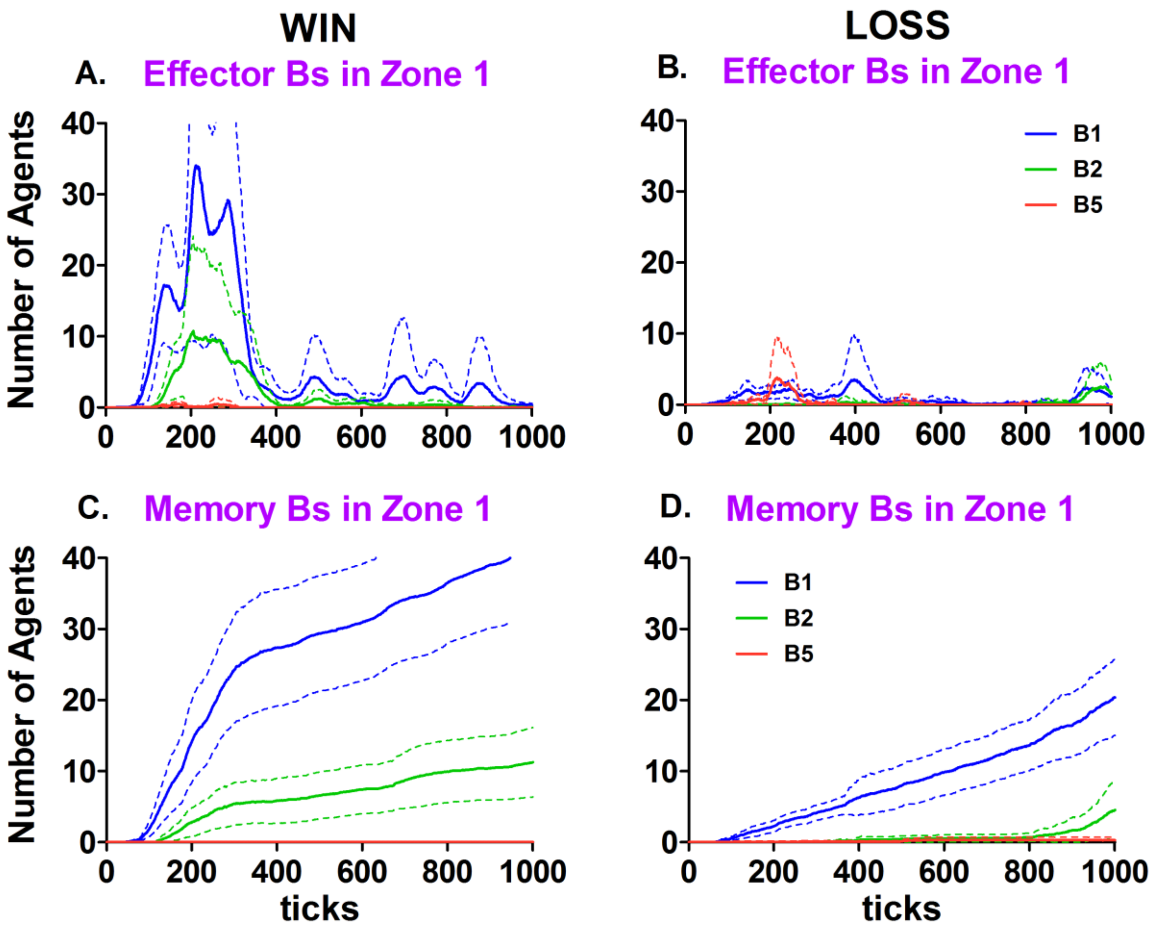

A. and B. The average number of Effector BCell Agents of types B1 (IgG_2-producing, blue), B2 (IgG_1-producing, green) and B5 (IgM-producing, red) ± the 95% confidence interval (solid line and dashed lines, respectively) for the *win* (A, n = 100) and *loss* (B, n = 46) outcomes is shown.
C. and D. The average number of Memory BCell Agents of types B1 (blue), B2 (green) and B5 (red) ± the 95% confidence interval (solid line and dashed lines, respectively) for the *win* (A, n = 100) and *loss* (B, n = 46) outcomes is shown.

The BCell Agents had similar contact patterns to the TCell Agents, and the same is true for the migration of effector and memory BCell Agents to Zone 1 after stimulation and proliferation in Zone 2 (additional files 13, 14, and 15). The average numbers of agents that migrated were greater in the *win* outcomes than the *loss* outcomes (A and C vs. B and D). More antibody was present in Zone 1 in the *win* outcome than the *loss* outcome (data not shown).
