# Supplementary material for: Using an agent-based model to analyze the dynamic communication network of the immune response
Source: Theor Biol Med Model. 2011 Jan 19;8:1. doi: 10.1186/1742-4682-8-1 (PMC3032717; doi:10.1186/1742-4682-8-1)
Supplement: Additional file 26 — The number of Effector and Memory CTL Agents in Zone 1 for the duration of the simulation for the win and loss outcomes. A figure that shows the average numbers of Effector and Memory CTL Agents in Zone 1 for the duration of the simulation. [file 1742-4682-8-1-S26.PDF]

Additional File 26. - The number of Effector and Memory CTL Agents in Zone 1 for the duration of the simulation for the *win* and *loss* outcomes.

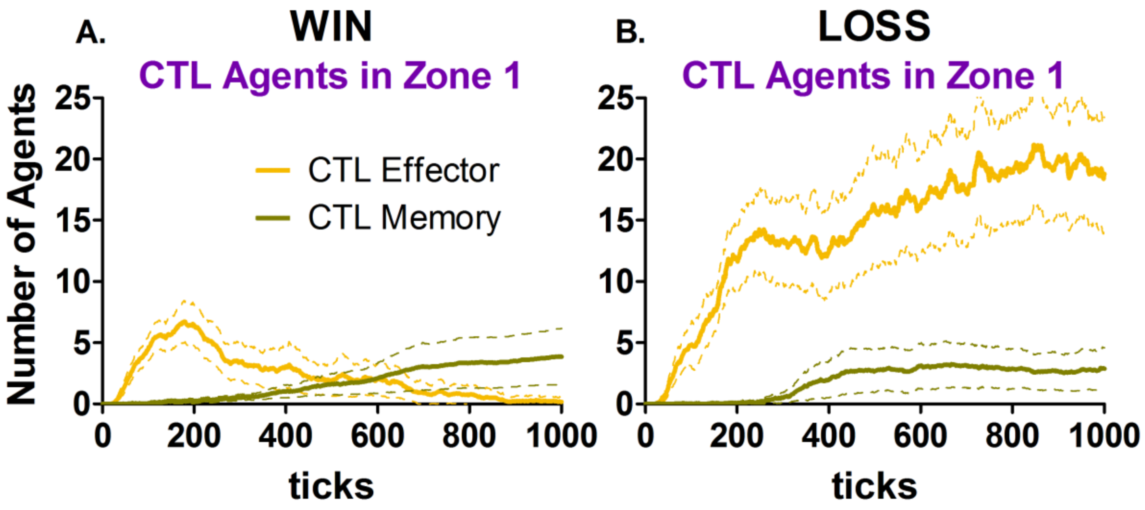

A. The average number of Effector CTL Agents (cytotoxic T-lymphocyte, gold) and Memory CTL Agents (olive green) ± the 95% confidence interval (solid line and dashed lines, respectively) for the *win* outcome (n = 100) is shown.
B. The average number of Effector CTL Agents (gold) and Memory CTL Agents (olive green) ± the 95% confidence interval (solid line and dashed lines, respectively) for the *loss* outcome (n = 46) is shown.

When the quantities of CTL Agents that made their way to Zone 1 to kill infected Parenchymal Agents were compared for the *win* and *loss* outcomes, significantly more effector CTL Agents were present for the *loss* outcome. This appears to be due to the continued stimulus of Dendritic Agents by infected Parenchymal Agents, which leads to their migration to Zone 2 where they activate more CTL Agents. The CTL Agents also made more contacts in the loss outcome (Figure 6D). Without the added requirements for interaction with B lymphocytes to promote antibody production that T-helper lymphocytes have [89, 136], the CTL Agents are activated and ready to travel to Zone 1 and kill infected Parenchymal Agents sooner than the TCell Agents ([97]; Additional file 24).
