# Supplementary material for: Using an agent-based model to analyze the dynamic communication network of the immune response
Source: Theor Biol Med Model. 2011 Jan 19;8:1. doi: 10.1186/1742-4682-8-1 (PMC3032717; doi:10.1186/1742-4682-8-1)
Supplement: Additional file 27 — The number of Macrophage Agents in Zone 1 for the duration of the simulation for the win and loss outcomes. A figure that shows the average numbers of Macrophage Agents in Zone 1 for the duration of the simulation. [file 1742-4682-8-1-S27.PDF]

Additional file 27 - The number of Macrophage Agents in Zone 1 for the duration of the simulation for the *win* and *loss* outcomes.

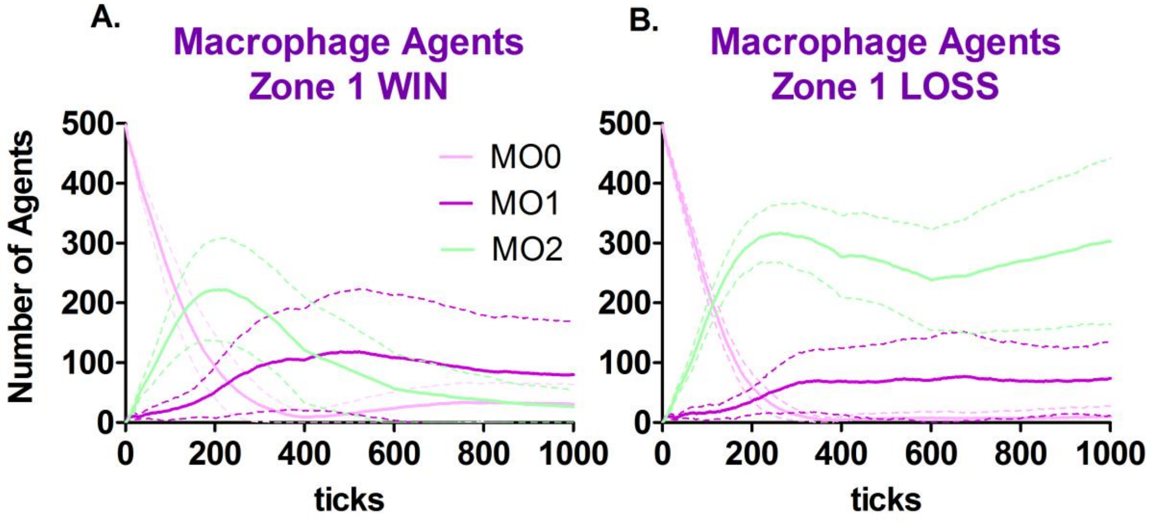

A. The average number of MO0 (monocytes, pink), MO1 (pro-inflammatory macrophages, purple), and MO2 (anti-inflammatory macrophages, light green) ± the 95% confidence interval (solid line and dashed lines, respectively) for the *win* outcome (n = 100) is shown.
B. The average number of MO0 (monocytes, pink), MO1 (pro-inflammatory macrophages, purple), and MO2 (anti-inflammatory macrophages, light green) ± the 95% confidence interval (solid line and dashed lines, respectively) for the *loss* outcome (n = 46) is shown.

The Macrophage Agents present in Zone 1 begin each simulation run uncommitted to pro- or anti-inflammatory states (MO0). The environmental conditions determine their fate (additional files 10, 11, and 12). A preponderance of apoptotic agents (mainly Parenchymal Agents, initially) and anti-inflammatory cytokines or lack of cytokines causes the Macrophage Agents to respond by becoming anti-inflammatory (MO2) [137]. Pro-inflammatory cytokines or contact with dead agents and necrotic debris cause the Macrophage Agents to become pro-inflammatory MO1s. There does not appear to be a difference in the quantities of MO1s between the *win* and *loss* outcomes, but more MO2s are present in the *loss* outcome.
