# Supplementary material for: Using an agent-based model to analyze the dynamic communication network of the immune response
Source: Theor Biol Med Model. 2011 Jan 19;8:1. doi: 10.1186/1742-4682-8-1 (PMC3032717; doi:10.1186/1742-4682-8-1)
Supplement: Additional file 28 — The number of Natural Killer Agents in Zone 1 for the duration of the simulation for the win and loss outcomes. A figure that shows the average numbers of Natural Killer Agents in Zone 1 for the duration of the simulation. [file 1742-4682-8-1-S28.PDF]

Additional file 28 - The number of Natural Killer Agents in Zone 1 for the duration of the simulation for the *win* and *loss* outcomes.

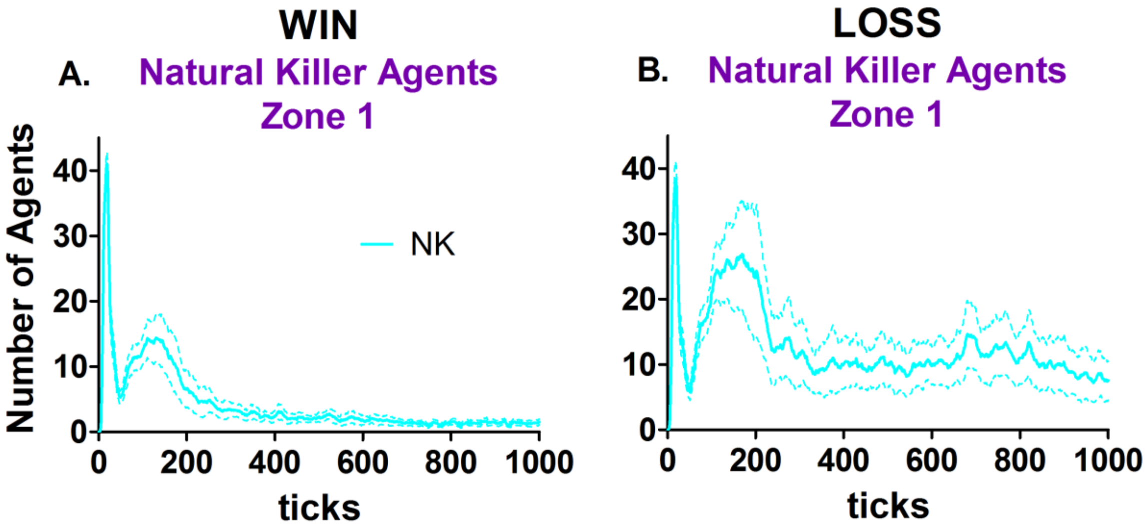

A. The average number of NK (natural killers, turquoise) ± the 95% confidence interval (solid line and dashed lines, respectively) for the *win* outcome (n = 100) is shown.
B. The average number of NK ± the 95% confidence interval (solid line and dashed lines, respectively) for the *loss* outcome (n = 46) is shown.

There were more Natural Killer Agents in Zone 1 for the *loss* outcome after about 50 ticks. This follows the pattern of more infected Parenchymal Agents in the *loss* outcome (Figure 2) as well as more activated Dendritic Agents (Figure 4). Both of these agents make signals that will call Natural Killer Agents into the site of infection in Zone 1.
