# Supplementary material for: Using an agent-based model to analyze the dynamic communication network of the immune response
Source: Theor Biol Med Model. 2011 Jan 19;8:1. doi: 10.1186/1742-4682-8-1 (PMC3032717; doi:10.1186/1742-4682-8-1)
Supplement: Additional file 29 — The number of Granulocyte Agents in Zone 1 for the duration of the simulation for the win and loss outcomes. A figure that shows the average numbers of Granulocyte Agents in Zone 1 for the duration of the simulation. [file 1742-4682-8-1-S29.PDF]

Additional file 29 - The number of Granulocyte Agents in Zone 1 for the duration of the simulation for the *win* and *loss* outcomes.

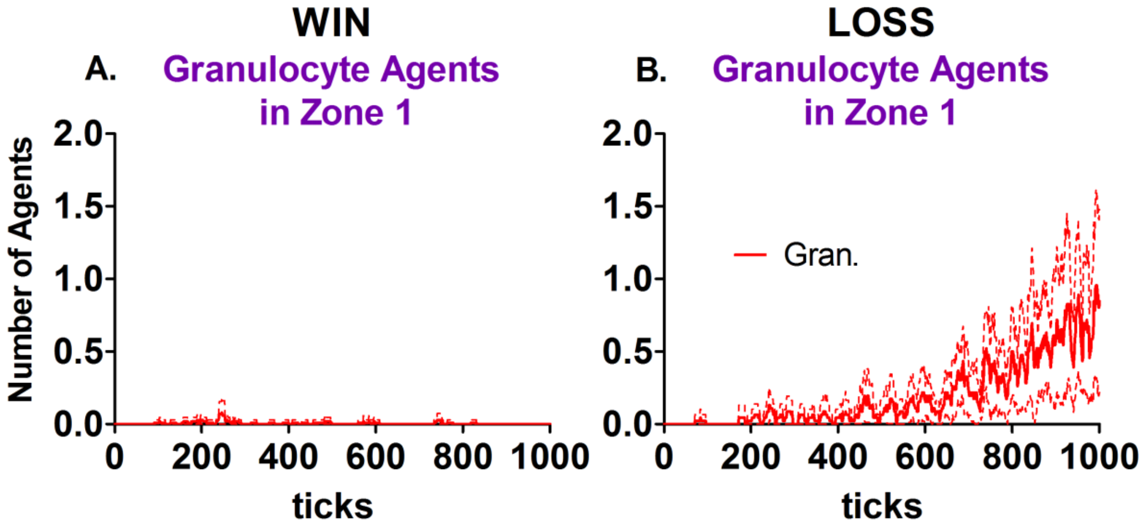

A. The average number of Granulocyte Agents (red) ± the 95% confidence interval (solid line and dashed lines, respectively) for the *win* outcome (n = 100) is shown.
B. The average number of Granulocyte Agents (red) ± the 95% confidence interval (solid line and dashed lines, respectively) for the *loss* outcome (n = 46) is shown.

Of all of the agents representing cells of innate immunity, the Granulocyte Agents participated the least in the simulations (Figures 7 and 8). Granulocytes are responsible for killing and phagocytosing bacteria, parasites and fungi [31]. They are also attracted to tissue environments where inflammation is occurring [118, 119], and they often cause damage to tissue in their efforts to kill [32]. In a viral infection their participation tends to be minimal unless other immune cells are causing inflammation and damage [120]. The minimal response observed in the *loss* outcome is consistent with the role that granulocytes play in a viral response
