# Supplementary material for: Using an agent-based model to analyze the dynamic communication network of the immune response
Source: Theor Biol Med Model. 2011 Jan 19;8:1. doi: 10.1186/1742-4682-8-1 (PMC3032717; doi:10.1186/1742-4682-8-1)
Supplement: Additional file 30 — Additional file references. A list of references cited in all of the additional files. [file 1742-4682-8-1-S30.PDF]

Additional file 30 - Additional file references.

References

1. Beilhack A, Rockson SG: **Immune traffic: A functional overview.** *Lymphatic Research and Biology* 2003, **1:**219-234.
2. Srivastava P: **Roles of heat shock protein in innate and adaptive immunity.** *Nature Reviews Immunology* 2002, **2:**185-194.
3. Shi Y, J.E. E, Rock KL: **Molecular identification of a danger signal that alerts the immune system to dying cells.** *Nature* 2003, **425:**516-521.
4. Wittamer V, Franssen J-D, Vulcano M, Mirjolet J-F, Poul EL, Migeotte I, Brezillon S, Tyldesley R, Blanpain C, Detheux M, Montovani A, Sozzani S, Vassart G, Parmentier M, Communi D: **Specific recruitment of antigen-presenting cells by chemerin, a novel processed ligand from human inflammatory fluids.** *Journal of Experimental Medicine* 2003, **198:**977-985.
5. Lotze MT, Tracey KJ: **High-mobility group box 1 (HMGB1): Nuclear weapon in the immune arsenal.** *Nature Reviews Immunology* 2005, **5:**331-342.
6. Yanai H, Ban T, Wang Z, Choi MK, Kawamura T, Negishi H, Nakasato M, Lu Y, Hangai S, Koshiba R, Savitsky D, Rofani L, Akira S, Bianchi ME, Honda K, Tamura T, Kodama T, Taniguchi T: **HMGB proteins function as universal sentinels for nucleic-acid-mediated innate immune responses.** *Nature* 2009, **462**.
7. Koch F, Stanzl U, Jennewein P, Janke K, Heufler C, Kampgen E, Romani N, Schuler G: **High level IL-12 production by murine dendritic cells: Upregulation via MHC Class II and CD40 molecules and downregulation by IL-4 and IL-10.** *Journal of Experimental Medicine* 1996, **184:**741-746.
8. Manel N, Unutmaz D, Littman DR: **The differentiation of human Th-17 cells requires transforming growth factor-B and induction of the nuclear receptor RORyt.** *Nature Immunology* 2008, **9:**641-649.
9. Volpe E, Servant N, Zollinger R, Bogiatzi SI, Hupe P, Barillot E, Soumelis V: **A critical function for transforming growth factor-B, interleukin 23 and proinflammatory cytokines in driving and modulating human Th-17 responses.** *Nature Immunology* 2008, **9:**650-657.
10. Yoshimura T, Matsushima K, Tanaka S, Robinson EA, Appella E, Oppenheim JJ, Leonard EJ: **Purification of a human monocyte-derived neutrophil chemotactic factor that has peptide sequence similarity to other host defense cytokines.** *The Proceedings of the National Academy of Sciences, USA* 1987, **84:**9233-9237.
11. Zhou L, Ivanov II, Spolski R, Min R, Shenderov K, Egawa T, Levy DE, Leonard WJ, Littman DR: **IL-6 programs Th-17 cell differentiation by promoting sequential engagement of the IL-21 and IL-23 pathways.** *Nature Immunology* 2007, **8:**967-974.
12. Langrish CL, Chen Y, Blumenschein WM, Mattson J, Basham B, Sedgwick JD, McClanahan T, Kastelein RA, Cua DJ: **IL-23 drives a pathogenic T cell population that induces autoimmune inflammation.** *The Journal of Experimental Medicine* 2005, **201:**233-240.
13. Hunter CA: **New IL-12-family members: IL-23 and IL-27, cytokines with divergent functions.** *Nature Reviews Immunology* 2005, **5:**521-531.
14. Ivanov S, Bozinovski S, Bossios A, Valadi H, Vlahos R, Malmhall C, Sjostrand M, Kolls JK, Anderson GP, Linden A: **Functional relevance of the IL-23-IL-17 axis in lungs in vivo.** *American Journal of Respiratory Cell and Molecular Biology* 2007, **36:**442-451.
15. Wilson NJ, Boniface K, Chan JR, McKenzie BS, Blumenschein WM, Mattson JD, Basham B, Smith K, Chen T, Morel F, Lecron J-C, Kastelein RA, Cua DJ, McClanahan TK, Bowman EP, de Waal Malefyt R: **Development, cytokine profile and function of human interleukin 17- producing helper T cells.** *Nature Immunology* 2007, **8:**950-957.

16. Harrington LE, Hatton RD, Mangan PR, Turner H, Murphy TL, Murphy KM, Weaver CT: **Interleukin 17-producing CD4+ effector T cells develop via a lineage distinct from the T helper type 1 and 2 lineages.** *Nature Immunology* 2005, **6:**1123-1132.
17. Villarino AV, Huang E, Hunter CA: **Understanding the pro- and anti-inflammatory properties of IL-27.** *The Journal of Immunology* 2003, **173:**715-720.
18. Chang JT, Palanivel VR, Kinjyo I, Schambach F, Intlekofer AM, Banerjee A, Longworth SA, Vinup KE, Mrass P, Oliaro J, Killeen N, Orange JS, Russell SM, Weninger W, Reiner SL: **Asymmetric T lymphocyte division in the initiation of adaptive immune responses.** *Science* 2007, **315:**1687-1691.
19. Park H, Li Z, Yang XO, Chang SH, Nurieva R, Wang Y-H, Wang Y, Hood L, Zhu Z, Tian Q, Dong C: **A distinct lineage of CD4 T cells regulates tissue inflammation by producing interleukin 17.** *Nature Immunology* 2005, **6:**1133-1141.
20. Yang L, Anderson DE, Baecher-Allan C, Hastings WD, Bettelli E, Oukka M, Kuchroo VK, Hafler DA: **IL-21 and TGF-β are required for differentiation of human Th17 cells.** *Nature* 2008:1-4.
21. Korn T, Bettelli E, Gao W, Awasthi A, Jager A, Strom TB, Oukka M, Kuchroo VK: **IL-21 initiates an alternative pathway to induce proinflammatory Th17 cells.** *Nature* 2007, **448:**484-488.
22. Mangan PR, Harrington LE, O'Quinn DB, Helms WS, Bullard DC, Elson CO, Hatton RD, Wahl SM, Schoeb TR, Weaver CT: **Transforming growth factor-β induces development of the T_H17 lineage.** *Nature* 2006, **441:**231-234.
23. O'Connor W, Kamanaka M, Booth CJ, Town T, Nakae S, Iwakura Y, Kolls JK, Flavell RA: **A protective function for interleukin 17A in T cell-mediated intestinal inflammation.** *Nature Immunology* 2009, **10:**603-610.
24. Roitt I, Brostoff J, Male D: *Immunology.* 6 edn. London: Mosby, Harcourt Publishers Ltd.; 2001.
25. Delves PJ, Martin SJ, Burton DR, Roitt IM: *Roitt's Essential Immunology.* Eleventh edn. Malden, MA: Blackwell Publishing Ltd.; 2006.
26. Mestecky J, Ogra P, McGhee J, Lambrecht BN, Strober W (Eds.): **Mucosal Immunology**, Third edition. Boston: Elsevier Academic Press; 2005.
27. Guo R-F, Ward PA: **Role of C5a in inflammatory responses.** *Annu Rev Immunol* 2005, **23:**821-852.
28. Green DR, Droin N, Pinkoski M: **Activation-induced cell death in T cells.** *Immunological Reviews* 2003, **193:**70-81.
29. Michaelsson J, de Matos CT, Achour A, Lanier LL, Karre K, Soderstrom K: **A signal peptide derived from hsp60 binds HLA-E and interferes with CD94/NKG2A recognition.** *J Exp Med* 2002, **196:**1403-1414.
30. Casadevall A, Pirofski L: **Antibody-mediated regulation of cellular immunity and the inflammatory response.** *TRENDS in Immunology* 2003, **24:**474-478.
31. Segal AW: **How neutrophils kill microbes.** *Annu Rev Immunol* 2005, **23:**197-223.
32. Ricevuti G: **Host tissue damage by phagocytes.** *Annals of the New York Academy of Sciences* 1997, **832:**426-448.
33. Huynh M-LN, Fadok VA, Henson PM: **Phosphatidylserine-dependent ingestion of apoptotic cells promotes TGF-β1 secretion and the resolution of inflammation.** *The Journal of Clinical Investigation* 2002, **109:**41-50.
34. Hart DNJ: **Dendritic Cells: Unique leukocyte populations which control the primary immune response. .** *Blood* 1997, **90:**3245-3287.
35. Gallicci S, Lolkema M, Matzinger P: **Natural Adjuvants: Endogenous activators of dendritic cells.** *Nature Medicine* 1999, **5:**1249-1255.
36. Vieira PL, de Jong EC, Wierenga EA, Kapsenberg ML, Kalinski P: **Development of Th1-inducing capacity in myeloid dendritic cells requires environmental instruction.** *J Immunol* 2000, **164:**4507-4512.
37. Zuniga EI, McGavern DB, Pruneda-Paz JL, Teng C, Oldstone MBA: **Bone marrow plasmacytoid dendritic cells can differentiate into myeloid dendritic cells upon virus infection.** *Nature Immunology* 2004, **5:**1227-1234.

38. Takeguchi O, Akira S: **Innate immunity to virus infection.** *Immunological Reviews* 2009, **227:**75-86.
39. Anderson CF, Lucas M, Gutierrez-Kobeh L, Field AE, Mosser DM: **T cell biasing by activated dendritic cells.** *J Immunol* 2004, **173:**955-961.
40. Moser M, Murphy KM: **Dendritic cell regulation of T_H1-T_H2 development.** *Nature Immunology* 2000, **1:**199-205.
41. Tanaka H, Demeure CE, Rubio M, Delespesse G, Sarfati M: **Human monocyte-derived dendritic cells induce naïve T cell differentiation into T helper cell type 2 (Th2) or Th1/Th2 effectors: Role of stimulator/responder ratio.** *J Exp Med* 2000, **192:**405-411.
42. Lodoen MB, Lanier LL: **Natural killer cells as an initial defense against pathogens.** *Current Opinion in Immunology* 2006, **18:**391-398.
43. Oppmann B, Lesley R, Blom B, Timans JC, Xu Y, Hunte B, Vega F, Yu N, Wang J, Singh K, Zonin F, Vaisberg E, Churakova T, Liu M, Gorman D, Wagner J, Zurawski S, Liu Y-J, Abrams JS, Moore KW, Rennick D, de Waal-Malefyt R, Hannum C, Bazan JF, Kastelein RA: **Novel p19 protein engages IL-12p40 to form a cytokine, IL-23, with biological activities similar as well as distinct from IL-12** *Immunity* 2000, **13:**715-725.
44. Jones SA: **Directing transition from innate to acquired immunity: Defining a role for IL-6.** *The Journal of Immunology* 2005, **175:**3463-3468.
45. Couper KN, Blount DG, Riley EM: **IL-10: The master regulator of immunity to infection.** *The Journal of Immunology* 2008, **180:**5771-5777.
46. Dubois B, Bridon J-M, Fayette J, Barthelemy C, Banchereau J, Caux C, Briere F: **Dendritic cells directly modulate B cell growth and differentiation.** *Journal of Leukocyte Biology* 1999, **66:**224-230.
47. Ingulli E, Mondino A, Khoruts A, Jenkins MK: **In vivo detection of dendritic cell antigen presentation to CD4+ T cells.** *Journal of Experimental Medicine* 1997, **185:**2133-2141.
48. Wu L, D'Amico A, Winkel KD, Suter M, Lo D, Shortman K: **RelB is essential for the development of myeloid-related CD8α- dendritic cells but not of lymphoid-related CD8α+ dendritic cells. .** *Immunity* 1998, **9:**839-847.
49. Anderson CF, Mosser DM: **Cutting edge: Biasing immune responses by directing antigen to macrophage Fcg receptors.** *Journal of Immunology* 2002, **168:**3697-3701.
50. Bajénoff M, Granjeaud S, Guerder S: **The strategy of T cell antigen-presenting cell encounter in antigen-draining lymph nodes revealed by imaging of initial T cell activation.** *J Exp Med* 2003, **198:**715-724.
51. Amsen D, Blander JM, Lee GR, Tanigaki K, Hongo T, Flavell RA: **Instruction of distinct CD4 T helper cell fates by different notch ligands on antigen-presenting cells.** *Cell* 2004, **117:**515-526.
52. Miller MJ, Hejazi AS, Wei SH, Cahalan MD, Parker I: **T cell repertoire scanning is promoted by dynamic dendritic cell behavior and random T cell motility in the lymph node.** *Proc Natl Acad Sci* 2004, **101:**998-1003.
53. Heath WR, Belz GT, Behrens GMN, Smith CM, Forehan SP, Parish IA, Davey GM, Wilson NS, Carbone FR, Villadangos JA: **Cross-presentation, dendritic cell subsets, and the generation of immunity to cellular antigens.** *Immunological Reviews* 2004, **199:**9-26.
54. Lindquist RL, Shakhar G, Dudziak D, Wardemann H, Eisenreich T, Dustin ML, Nussenzweig MC: **Visualizing dendritic cell networks in vivo.** *Nature Immunology* 2004, **5:**1243-1250.
55. Kriehuber E, Bauer W, Charbonnier AS, Winter D, Amatschek S, Tamandl D, Schweifer N, Stingl G, Maurer D: **Balance between NF-κB and JNK/AP-1 activity controls dendritic cell life and death.** *Blood* 2005, **106:**175-183.
56. Beltman JB, Maree AFM, Lynch JN, Miller MJ, deBoer RJ: **Lymph node topology dictates T cell migration behavior.** *The Journal of Experimental Medicine* 2007, **204:**771-780.
57. Germain RN, Bajenoff M, Castellino F, Chieppa M, Egen JG, Huang AYC, Ishii M, Koo LY, Qi H: **Making friends in out-of-the-way places: how cells of the immune system get together and how they conduct their business as revealed by intravital imaging.** *Immunological Reviews* 2008, **221:**163-181.

58. Henson PM, Bratton DL, Fadok VA: **Apoptotic cell removal.** *Current Biology* 2001, **11:**R795-R805.
59. Miga AJ, Masters SR, Durell BG, Gonzales M, Jenkins MK, Maliszewski C, Kikutani H, Wade WF, Noelle RJ: **Dendritic Cell longevity and T cell persistence is controlled by CD154-CD40 interactions.** *Eur J Immunol* 2001, **31:**959-965.
60. Hou W-S, Van Parijs L: **A Bcl-2-dependent molecular timer regulates the lifespan and immunogenicity of dendritic cells.** *Nature Immunology* 2004, **5:**583-589.
61. Matsue H, Edelbaum D, Hartmann AC, Morita A, Bergstresser PR, Yagita H, Okumura K, Takashima A: **Dendritic cells undergo rapid apoptosis in vitro during antigen-specific interaction with CD4+ T cells.** *The Journal of Immunology* 1999, **162:**5287-5298.
62. Guarda G, Hons M, Soriano SF, Huang AY, Polley R, Martin-Fontecha A, Stein JV, Germain RN, Lanzavecchia A, Sallusto F: **L-selectin-negative CCR7- effector and memory CD8+ T cells enter reactive lymph nodes and kill dendritic cells.** *Nature Immunology* 2007, **8:**743-752.
63. Mosser DM: **The many faces of macrophage activation.** *Journal of Leukocyte Biology* 2003, **73:**209-212.
64. Matzinger P: **Friendly and dangerous signals: is the tissue in control?** *Nature Immunology* 2007, **8:**11-13.
65. Sansonetti PJ: **The innate signaling of dangers and the dangers of innate signaling.** *Nature Immunology* 2006, **7:**1237-1242.
66. Fadok VA, Bratton DL, Konowal A, Freed PW, Westcott JY, Henson PM: **Macrophages that have ingested apoptotic cells in vitro inhibit proinflammatory cytokine production through autocrine/paracrine mechanisms involving TGF-β, PGE2, and PAF** *Journal of Clinical Investigation* 1998, **101:**890-898.
67. Sixt M, Kanazawa N, Selg M, Samson T, Roos G, Reinhardt DP, Pabst R, Lutz MB, Sorokin L: **The conduit system transports soluble antigens from the afferent lymph to resident dendritic cells in the T cell area of the lymph node.** *Immunity* 2005, **22:**19-29.
68. Xu W, Berger SP, Trouw LA, Boer HCd, Schlagwein N, Mutsaers C, Daha MR, vanKooten C: **Properdin binds to late apoptotic and necrotic cells independently of C3b and regulates alternative pathway complement activation.** *The Journal of Immunology* 2008, **180:**7613-7621.
69. Yamasaki S, Ishikawa E, Sakuma M, Hara H, Ogata K, Saito T: **Mincle is an ITAM-coupled activating receptor that senses damaged cells.** *Nature Immunology* 2008, **9:**1179-1188.
70. Geissmann F, Manz MG, Jung S, Sieweke MH, Merad M, Ley K: **Development of monocytes, macrophages and dendritic cells.** *Science* 2010, **327:**656-661.
71. Khalil N: **TGF-β: from latent to active.** *Microbes and Infection* 1999, **1:**1255-1263.
72. Kastelein RA, Hunter CA, Cua DJ: **Discovery and biology of IL-23 and IL-27: Related but functionally distinct regulators of inflammation.** *Annual Review of Immunology* 2007, **25:**221-242.
73. Hoffmann PR, deCathelineau AM, Ogden CA, Leverrier Y, Bratton DL, Daleke DL, Ridley AJ, Fadok VA, Henson PM: **Phosphatidylserine (PS) induces PS receptors-mediated macrophinocytosis and promotes clearance of apoptotic cells.** *The Journal of Cell Biology* 2001, **155:**649-659.
74. Kim HS, Lee M-S: **Essential role of STAT1 in caspase-independent cell death of activated macrophages through the p38 mitogen-activated protein kinase/STAT1/reactive oxygen species pathway.** *Molecular and Cellular Biology* 2005, **25:**6821-6833.
75. Xu Y, Kim SO, Han J: **Autophagy contributes to caspase-indepedent macrophage cell death.** *The Journal of Biological Chemistry* 2006, **281:**19179-19187.
76. Macatonia SE, Hosken NA, Litton M, Vieira P, Hsieh C-S, Culpepper JA, Wysocka M, Trinchieri G, Murphy KM, O'Garra A: **Dendritic cells produce IL-12 and direct the development of Th1 cells from naive CD4+ T cells.** *The Journal of Immunology* 1995, **154:**5071-5079.
77. Heufler C, Koch F, Stanzl U, Topar G, Wysocka M, Trinchieri G, Enk A, Steinman RM, Romani N, Schuler G: **Interleukin-12 is produced by dendritic cells and mediates T helper 1 development as well as interferon-γ production by T helper 1 cells.** *European Journal of Immunology* 1996, **26:**659-668.

78. Stumbles PA, Thomas JA, Pimm CL, Lee PT, Venaille TJ, Proksch S, Holt PG: **Resting respiratory tract dendritic cells preferentially stimulate T helper cell 2 (Th2) responses and require obligatory cytokine signals for induction of Th1 immunity.** *Journal of Experimental Medicine* 1998, **188:**2019-2031.
79. Pflanz S, Timans JC, Cheung J, Rosales R, Kanzler H, Gilbert J, Hibbert L, Churakova T, Travis M, Vaisberg E, Blumenschein WM, Mattson JD, Wagner JL, To W, Zurawski S, McClanahan TK, Gorman DM, Bazan JF, de Waal Malefyt R, Rennick D, Kastelein RA: **IL-27, a heterodimeric cytokine composed of EBI3 and p28 protein, induces proliferation of naive CD4+ T cells.** *Immunity* 2002, **16:**779-790.
80. Malek TR: **The biology of interleukin-2.** *Annual Review of Immunology* 2008, **26:**453-479.
81. Liu J, Lin F, Strainic MG, An F, Miller RH, Altuntas CZ, Heeger PS, Tuohy VK, Medof ME: **IFN-y and IL-17 production in experimental autoimmune encephalomyelitis depends on local APC-T cell complement production.** *The Journal of Immunology* 2008**:**5882-5889.
82. McKinstry KK, Golech S, Lee W-H, Huston G, Weng N-P, Swain SL: **Rapid default transition of CD4+ T cell effectors to functional memory cells.** *Journal of Experimental Medicine* 2007, **204:**2199-2211.
83. Nurieva R, Yang XO, Martinez G, Zhang Y, Panopoulos AD, Ma L, Schluns K, Tian Q, Watowich SS, Jetten AM, Dong C: **Essential autocrine regulation in the generation of inflammatory T cells.** *Nature* 2007, **448:**480-484.
84. Pandiyan P, Zheng L, Ishihara S, Reed J, Lenardo MJ: **CD4+CD25+Foxp3+ regulatory T cells induce cytokine deprivation-mediated apoptosis of effector CD4+ T cells.** *Nature Immunology* 2007, **8:**1353-1362.
85. Vogelzang A, McGuire HM, Yu D, Sprent J, Mackay CR, King C: **A fundamental role for interleukin-21 in the generation of T follicular helper cells.** *Immunity* 2008, **29:**127-137.
86. Wu C-Y, Kirman JR, Rotte MJ, Davey DF, Perfetto SP, Rhee EG, Freidag BL, Hill BJ, Douek DC, Seder RA: **Distinct lineages of T_H1 cells have differential capacities for memory cell generation in vivo.** *Nature Immunology* 2002, **3:**852-858.
87. McHeyzer-Williams LJ, McHeyzer-Williams MG: **Antigen specific memory B cell development.** *Annu Rev Immunol* 2005, **23:**487-513.
88. Brocker T: **Survival of mature CD4 T lymphocytes is dependent on major histocompatibility complex class II-expressing dendritic cells.** *J Exp Med* 1997, **186:**1223-1232.
89. Garside P, Ingulli E, Merica RR, Johnson JG, Noelle RJ, Jenkins MK: **Visualization of specific B and T lymphocyte interactions in the lymph node.** *Science* 1998, **281:**96-99.
90. Mempel TR, Henrickson SE, vonAndrian UH: **T-cell priming by dendritic cells in lymph nodes occurs in three distinct phases.** *Nature* 2004, **427:**154-159.
91. Chen W, Jin W, Tian H, Sicurello P, Frank M, Orenstein JM, Wahl SM: **Requirement for transforming growth factor β1 in controlling T cell apoptosis.** *The Journal of Experimental Medicine* 2001, **194:**439-453.
92. Devadas S, Das J, Liu C, Zhang L, Roberts AI, Pan Z, Moore PA, Das G, Shi Y: **Granzyme B is critical for T cell receptor-induced cell death of type 2 helper T cells.** *Immunity* 2006, **25:**237-247.
93. Foulds KE, Rotte MJ, Paley MA, Singh B, Douek DC, Hill BJ, O'Shea JJ, Watford WT, Seder RA, Wu C-Y: **IFN-gamma mediates the death of Th1 cells in a paracrine manner.** *The Journal of Immunology* 2007, **180:**842-849.
94. Refaeli Y, Parijs LV, Alexander SI, Abbas AK: **Interferon γ is required for activation-induced death of T lymphocytes** *Journal of Experimental Medicine* 2002, **196:**999-1005.
95. Badinovac VP, Messingham KAN, Jabbari A, Haring JS, Harty JT: **Accelerated CD8+ T-cell memory and prime-boost response after dendritic-cell vaccination.** *Nature Medicine* 2005, **11:**748-756.
96. Drake DR, Braciale TJ: **Not all effector CD8+ T cells are alike.** *Microbes and Infection* 2003, **5:**199-204.

97. Stetson DB, Mohrs M, Reinhardt RL, Baron JL, Wang Z-E, Gapin L, Kronenberg M, Locksley RM: **Constitutive cytokine mRNAs mark Natural Killer (NK) and NK T Cells poised for rapid effector function.** *J Exp Med* 2003, **198:**1069-1076.
98. Harty JT, Tvinnereim AR, White DW: **CD8+ T cell effector mechanisms in resistance to infection.** *Annual Review of Immunology* 2000, **18:**275-308.
99. Bousso P, Robey E: **Dynamics of CD8+ T cell priming by dendritic cells in intact lymph nodes.** *Nature Immunology* 2003, **4:**579-585.
100. Wherry EJ, Ahmed R: **Memory CD8 T-cell differentiantion during viral infection.** *Journal of Virology* 2004, **78:**5535-5545.
101. Regner M, Mullbacher A: **Granzymes in cytolytic lymphocytes - to kill a killer?** *Immunology and Cell Biology* 2004, **82:**161-169.
102. Salazar-Mather TP, Orange JS, Biron CA: **Early murine cytomegalovirus (MCMV) infection induces liver Natural Killer (NK) cell inflammation and protection through Macrophage Inflammatory Protein 1α (MIP-1α)-dependent pathways.** *J Exp Med* 1998, **187:**1-14.
103. Yu J, Wei M, Becknell B, Trotta R, Liu S, Boyd Z, Jaung MS, Blaser BW, Sun J, Don M. Benson J, Mao H, Yokohama A, Bhatt D, Shen L, Davuluri R, Weinstein M, Marcucci G, Caligiuri MA: **Pro- and antiinflammatory cytokine signaling: Reciprocal antagonism regulates interferon-gamma production by human natural killer cells.** *Immunity* 2006, **24:**575-590.
104. Smyth MJ, Cretney E, Kelly JM, Westwood JA, Street SEA, Yagita H, Takeda K, van Dommelen SLH, Degli-Esposti MA, Hayakawa Y: **Activation of NK cell cytotoxicity.** *Molecular Immunology* 2005, **42:**501-510.
105. Sun JC, Lanier LL: **Cutting edge: Viral infection breaks NK cell tolerance to "missing self"** *The Journal of Immunology* 2008, **181:**7453-7457.
106. Cassese G, Arce S, Hauser AE, Lehnert K, Moewes B, Mostarac M, Muehlinghaus G, Szyska M, Radbruch A, Manz RA: **Plasma cell survival is mediated by synergistic effects of cytokines and adhesion-dependent signals.** *Journal of Immunology* 2003, **171:**1684-1690.
107. Zotos D, Coquet JM, Zhang Y, Light A, D'Costa K, Kallies A, Corcoran LM, Godfrey DI, Toellner K-M, Smyth MJ, Nutt SL, Tarlington DM: **IL-21 regulates germinal center B cell differentiation and proliferation through a B cell-intrinsic mechanism.** *Journal of Experimental Medicine* 2010, **207:**365-378.
108. Ozaki K, Spolski R, Feng CG, Qi C-F, Cheng J, Sher A, Herbert C. Morse I, Liu C, Schwartzberg PL, Leonard WJ: **A critical role for IL-21 in regulating immunoglobulin production.** *Science* 2002, **298:**1630-1634.
109. Lopes-Carvalho T, Foote J, Kearney JF: **Marginal zone B cells in lymphocyte activation and regulation.** *Current Opinion in Immunology* 2005, **17:**244-250.
110. Dubois B, Massacrier C, Vanbervliet B, Fayette J, Briere F, Banchereau J, Caux C: **Critical role of IL-12 in dendritic cell-induced differentiation of naive B lymphocytes.** *The Journal of Immunology* 1998, **161:**2223-2231.
111. Cassese G, Lindenau S, DeBoer B, Arce S, Hauser A, Riemekasten G, Berek C, Hiepe F, Krenn V, Radbruch A, Manz RA: **Inflamed kidneys of NZB/W mice are a major site for the homeostasis of plasma cells.** *Eur J Immunol* 2001, **31:**2726-2732.
112. Crotty S, Felgner P, Davies H, Glidewell J, Villarreal L, Ahmed R: **Cutting edge: Long-term B cell memory in humans after smallpox vaccination.** *The Journal of Immunology* 2003, **171:**4969-4973.
113. McHeyzer-Williams LJ, Malherbe LP, McHeyzer-Williams MG: **Checkpoints in memory B-cell evolution.** *Immunological Reviews* 2006, **211:**255-268.
114. Benson MJ, Dillon SR, Castigli E, Geha RS, Xu S, Lam K-P, Noelle RJ: **Cutting edge: The dependence of plasma cells and independence of memory B cells on BAFF and APRIL.** *The Journal of Immunology* 2008, **180:**3655-3659.
115. Linterman MA, Beaton L, Yu D, Ramiscal RR, Srivastava M, Hogan JJ, Verma NK, Smyth MJ, Rigby RJ, Vinuesa CG: **IL-21 acts directly on B cells to regulate Bcl-6 expression and germinal center responses.** *Journal of Experimental Medicine* 2010, **207:**353-363.

116. Kantor AB, Herzenberg LA: **Origin of Murine B cell lineages.** *Annual Review of Immunology* 1993, **11:**501-538.
117. Ye P, Rodriguez FH, Kanaly S, Stocking KL, Schurr J, Schwarzenberger P, Oliver P, Huang W, Zhang P, Zhang J, Shellito JE, Bagby GJ, Nelson S, Charrier K, Peschon JJ, Kolls JK: **Requirement of interleukin 17 receptor signaling for lung CXC chemokine and granulocyte colony-stimulating factor expression, neutrophil recruitment, and host defense** *Journal of Experimental Medicine* 2001, **194:**519-527.
118. Struyf S, Gouwy M, Dillen C, Proost P, Opdenakker G, Damme JV: **Chemokines synergize in the recruitment of circulating neutrophils into inflammed tissue.** *European Journal of Immunology* 2005, **35:**1583-1591.
119. Ramos CDL, Canetti C, Souto JT, Silva JS, Hogaboam CM, Ferreira SH, Cunha FQ: **MIP-1α [CCL3] acting on the CCR1 receptor mediates neutrophil migration in immune inflammation via sequential release of TNF-α and LTB_4.** *Journal of Leukocyte Biology* 2005, **78:**167-177.
120. Parker LC, Whyte MKB, Dower SK, Sabroe I: **The expression and roles of Toll-like receptors in the biology of the human neutrophil.** *Journal of Leukocyte Biology* 2005, **77:**886-892.
121. Zhang Q, Raoof M, Chen Y, Sumi Y, Sursal T, Junger W, Brohi K, Itagaki K, Hauser CJ: **Circulating mitochondrial DAMPs cause inflammatory responses to injury.** *Nature* 2010, **464:**104-107.
122. Pham CTN: **Neutrophil serine proteases: specific regulators of inflammation.** *Nature Reviews Immunology* 2006, **6:**541-550.
123. Haslett C: **Granulocyte apoptosis and its role in the resolution and control of lung inflammation.** *American Journal of Respiratory and Critical Care Medicine* 1999, **160:**S5-S11.
124. Rydell-Tormanen K, Uller L, Erjefalt JS: **Direct evidence of secondary necrosis of neutrophils during intense lung inflammation.** *European Respiratory Journal* 2006, **28:**268-274.
125. Vermaelen K, Pauwels R: **Pulmonary dendritic cells.** *American Journal of Respiratory and Critical Care Medicine* 2005, **172:**530-551.
126. Krysko D, Denecker G, Festjens N, Gabriels S, Parthoens E, D'Herde K, Vandenabeele P: **Macrophages use different internalization mechanisms to clear apoptotic and necrotic cells.** *Nature: Cell Death and Differentiation* 2006, **13:**2011-2022.
127. Folcik VA, An GC, Orosz CG: **The Basic Immune Simulator: An Agent-Based Model to study the interactions between innate and adaptive immunity.** *Theoretical Biology and Medical Modelling* 2007, **4:**39.
128. Liu Y, Zhang P, Li J, Kulkarni AB, Perruche S, Chen W: **A critical function for TGF-b signaling in the development of natural CD4+CD25+Foxp3+ regulatory T cells** *Nature Immunology* 2008, **9:**632-640.
129. Nurieva RI, Chung Y, Hwang D, Yang XO, Kang HS, Ma L, Wang Y-h, Watowich SS, Jetten AM, Tian Q, Dong C: **Generation of T follicular helper cells is mediated by interleukin-21 but independent of T helper 1, 2, or 17 cell lineages.** *Immunity* 2008, **29:**138-149.
130. Wykes M, Pombo A, Jenkins C, MacPherson GG: **Dendritic cells interact directly with naive B lymphocytes to transfer antigen and initiate class switching in a primary T-dependent response.** *The Journal of Immunology* 1998, **161:**1313-1319.
131. Ridge JP, Rosa FD, Matzinger P: **A conditioned dendritic cell can be a temporal bridge between a CD4+ T-helper and a T-killer cell.** *Nature* 1998, **393:**474-478.
132. Bennett SRM, Carbone FR, Karamalis F, Flavell RA, Miller JFAP, Heath WR: **Help for cytotoxic-T-cell responses is mediated by CD40 signalling.** *Nature* 1998, **393:**478-480.
133. Schoenberger SP, Toes REM, vanderVoort EIH, Offringa R, Melief CJM: **T-cell help for cytotoxic T lymphocytes is mediated by CD40-CD40L interactions.** *Nature* 1998, **393:**480-483.
134. Weaver CT, Hatton RD, Mangan PR, Harrington LE: **IL-17 family cytokines and the expanding diversity of effector T cell lineages.** *Annual Review of Immunology* 2007, **25:**821-852.
135. Ishigame H, Kakuta S, Nagai T, Kadoki M, Nambu A, Komiyama Y, Fujikado N, Tanahashi Y, Akitsu A, Kotaki H, Sudo K, Nakae S, Sasakawa C, Iwakura Y: **Differential roles of interleukin-17A and 17F in host defense against mucoepithelial bacterial infection and allergic responses.** *Immunity* 2009, **30:**108-119.

136. Okada T, Miller MJ, Parker I, Krummel MF, Neighbors M, Hartley SB, O'Garra A, Cahalan MD, Cyster JG: **Antigen-engaged B cells undergo chemotaxis toward the T zone and form motile conjugates with helper T cells.** *PLoS Biology* 2005, **3:**e150.
137. Huynh M-LN, Fadok VA, Henson PM: **Phosphatidylserine-dependent ingestion of apoptotic cells promotes TGF-β1 secretion and the resolution of inflammation.** *The Journal of Clinical Investigation* 2002, **109:**41-50.
