# Supplementary material for: Using an agent-based model to analyze the dynamic communication network of the immune response
Source: Theor Biol Med Model. 2011 Jan 19;8:1. doi: 10.1186/1742-4682-8-1 (PMC3032717; doi:10.1186/1742-4682-8-1)
Supplement: Additional file 32 — Frequency distributions of contacts for each immune agent type at 1000 ticks with a starting condition of 100 Dendritic Agents. A figure that shows the frequency distribution of contacts for each immune agent type at the end of the simulation (120 simulation runs combined; 100 Dendritic Agents starting condition) with the win and loss outcomes separated. [file 1742-4682-8-1-S32.PDF]

Additional file 32 - Frequency distributions of contacts for each immune agent type at 1000 ticks with a starting condition of 100 Dendritic Agents.

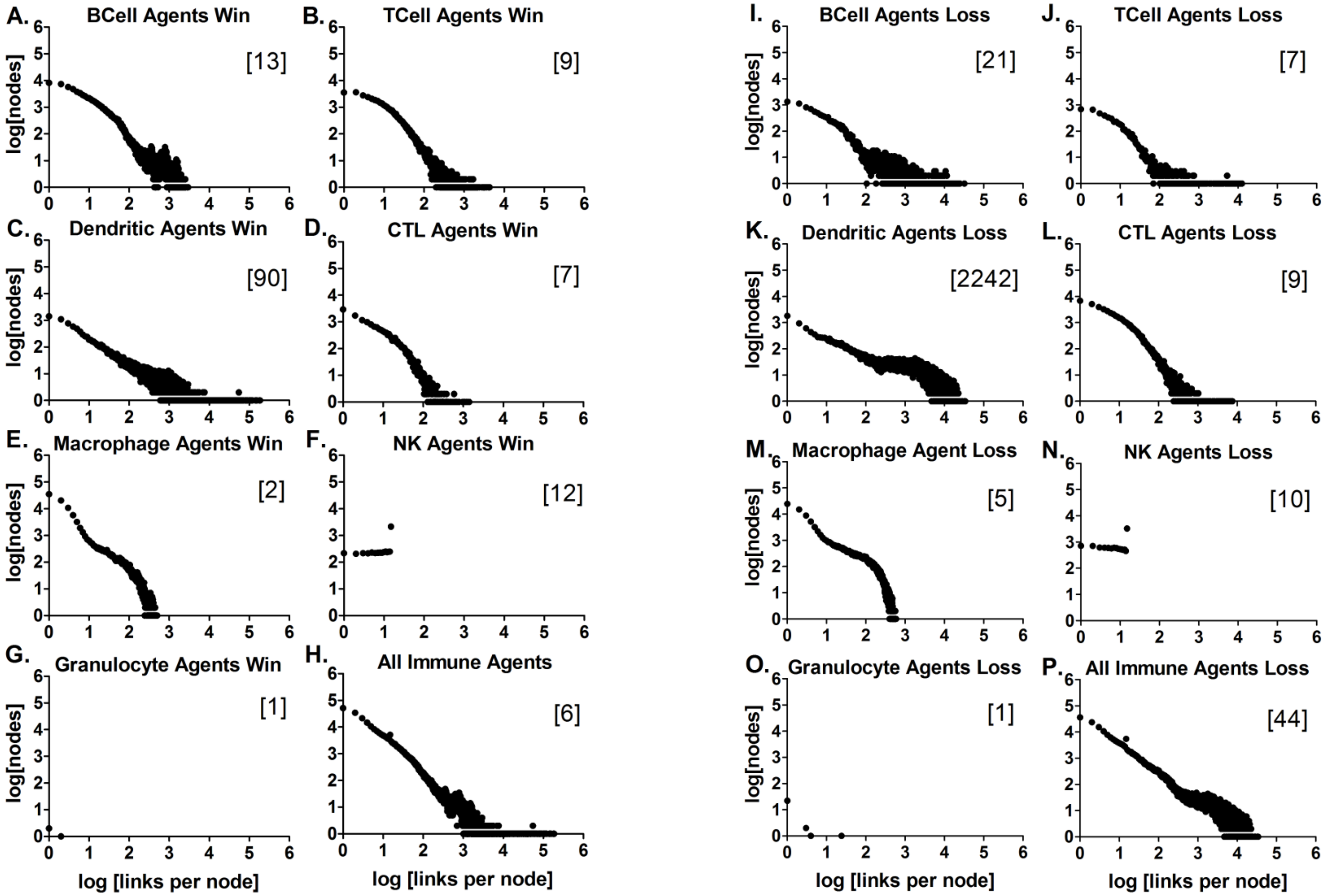

The frequency distribution of links per node on a log-log scale for each immune agent type is shown, with the combined runs ending in *win* and *loss* outcomes separated. The median number of links per node for the distribution is in brackets. The statistics for each distribution is in a Table in additional file 34.
