# Supplementary material for: Using an agent-based model to analyze the dynamic communication network of the immune response
Source: Theor Biol Med Model. 2011 Jan 19;8:1. doi: 10.1186/1742-4682-8-1 (PMC3032717; doi:10.1186/1742-4682-8-1)
Supplement: Additional file 34 — Table of statistics for Figure 8 and additional files 31, 32, and 33. A table of statistics including the number of data points, Spearman r value for correlation and the p-value for the correlation statistic for all of the frequency distribution diagrams for each agent shown in Figure 8 and additional files 31, 32, and 33. [file 1742-4682-8-1-S34.PDF]

Additional file 34 - Table of statistics for Figure 8 and additional files 31, 32, and 33.

Initial Conditions:	20 Dendritic Agents	100 Dendritic Agents	200 Dendritic Agents	300 Dendritic Agents
WIN:LOSS	39:81	63:57	100:46	92:28
All WIN	2119 points<br>r = -0.8745<br>$p < 0.0001$	3004 points<br>r = -0.9195<br>$p < 0.0001$	3603 points<br>r = -0.8657<br>$p < 0.0001$	4028 points<br>r = -0.8409<br>$p < 0.0001$
All LOSS	9732 points<br>r = -0.9288<br>$p < 0.0001$	15148 points<br>r = -0.9047<br>$p < 0.0001$	17250 points<br>r = -0.9097<br>$p < 0.0001$	21310 points<br>r = -0.8936<br>$p < 0.0001$
BCell Agents WIN	1409 points<br>r = -0.7596<br>$p < 0.0001$	1758 points<br>r = -0.8273<br>$p < 0.0001$	1732 points<br>r = -0.7197<br>$p < 0.0001$	1972 points<br>r = -0.7290<br>$p < 0.0001$
BCell Agents LOSS	3047 points<br>r = -0.8027<br>$p < 0.0001$	2353 points<br>r = -0.7932<br>$p < 0.0001$	2353 points<br>r = -0.6487<br>$p < 0.0001$	1529 points<br>r = -0.6170<br>$p < 0.0001$
TCell Agents WIN	493 points<br>r = -0.8712<br>$p < 0.0001$	692 points<br>r = -0.8686<br>$p < 0.0001$	756 points<br>r = -0.8636<br>$p < 0.0001$	777 points<br>r = -0.8621<br>$p < 0.0001$
TCell Agents LOSS	704 points<br>r = -0.8028<br>$p < 0.0001$	404 points<br>r = -0.7754<br>$p < 0.0001$	368 points<br>r = -0.8192<br>$p < 0.0001$	268 points<br>r = -0.7857<br>$p < 0.0001$
Dendritic Agents WIN	1805 points<br>r = -0.7668<br>$p < 0.0001$	2839 points<br>r = -0.8579<br>$p < 0.0001$	3444 points<br>r = -0.7669<br>$p < 0.0001$	3653 points<br>r = -0.7112<br>$p < 0.0001$
Dendritic Agents LOSS	9271 points<br>r = -0.9271<br>$p < 0.0001$	15081 points<br>r = -0.9016<br>$p < 0.0001$	17218 points<br>r = -0.9074<br>$p < 0.0001$	21267 points<br>r = -0.8908<br>$p < 0.0001$
CTL Agents WIN	219 points<br>r = -0.9249<br>$p < 0.0001$	282 points<br>r = -0.9414<br>$p < 0.0001$	216 points<br>r = -0.9488<br>$p < 0.0001$	301 points<br>r = -0.9176<br>$p < 0.0001$
CTL Agents LOSS	677 points<br>r = -0.9321<br>$p < 0.0001$	632 points<br>r = -0.9297<br>$p < 0.0001$	580 points<br>r = -0.9257<br>$p < 0.0001$	470 points<br>r = -0.9316<br>$p < 0.0001$
Macrophage Agents WIN	412 points<br>r = -0.9773<br>$p < 0.0001$	418 points<br>r = -0.9659<br>$p < 0.0001$	310 points<br>r = -0.9630<br>$p < 0.0001$	232 points<br>r = -0.9719<br>$p < 0.0001$
Macrophage Agents LOSS	578 points<br>r = -0.9935<br>$p < 0.0001$	517 points<br>r = -0.9894<br>$p < 0.0001$	470 points<br>r = -0.9896<br>$p < 0.0001$	438 points<br>r = -0.9729<br>$p < 0.0001$
NK Agents WIN	16 points<br>r = 0.1882<br>p = 0.4851	16 points<br>r = 0.5765<br>p = 0.0194	16 points<br>r = -0.6853<br>p = 0.0034	16 points<br>r = -0.6853<br>p = 0.0034
NK Agents LOSS	16 points<br>r = 0.5118<br>p = 0.0427	16 points<br>r = -0.6647<br>p = 0.0050	16 points<br>r = -0.6912<br>p = 0.0030	16 points<br>r = -0.7706<br>p = 0.0005
Granulocyte Agents WIN	3 points<br>ns	3 points<br>ns	2 points<br>na	2 points<br>na
Granulocyte Agents LOSS	4 points<br>ns	5 points<br>r = -0.9747<br>p = 0.0167	2 points<br>na	3 points<br>ns

r = Spearman correlation coefficient, ns = not significant, na = not applicable
